# A Study of the Bisphosphonic Derivatives from the Pudovik Reaction of Dialkyl α-Oxophosphonates and >P(O)H Reagents: X-ray Structure and Bioactivity

**DOI:** 10.3390/molecules28166037

**Published:** 2023-08-12

**Authors:** Zsuzsanna Szalai, Boldizsár Tóth, Rita Oláhné Szabó, Szilvia Bősze, Konstantin Karaghiosoff, Mátyás Czugler, László Drahos, György Keglevich

**Affiliations:** 1 Department of Organic Chemistry and Technology, Faculty of Chemical Technology and Biotechnology, Budapest University of Technology and Economics, 1521 Budapest, Hungary; sz.zsuzsi97@gmail.com (Z.S.); toth.boldi@hotmail.hu (B.T.); amatyasczx@protonmail.com (M.C.); 2 Eötvös Loránd Research Network (ELKH), Research Group of Peptide Chemistry, Eötvös Loránd University, 1117 Budapest, Hungary; rita.szabo@ttk.elte.hu (R.O.S.); szilvia.bosze@gmail.com (S.B.); 3 Department of Genetics, Cell and Immunobiology, Semmelweis University, Nagyvárad tér 4, 1089 Budapest, Hungary; 4 Department Chemie, Ludwig-Maximilians-Universitat München, Butenandtstr. 5-13, D-81377 München, Germany; klk@cup.uni-muenchen.de; 5 MS Proteomics Research Group, Research Centre for Natural Sciences, 1117 Budapest, Hungary; drahos.laszlo@ttk.hu

**Keywords:** hydroxy-methylenebisphosphonic derivatives, α-oxophosphonates, dialkyl phosphites, secondary phosphine oxides, Pudovik reaction, rearrangement, X-ray structures, cytotoxic effect

## Abstract

New hydroxy-methylenebisphosphonic derivatives were prepared with different P-functions. The outcome of the reaction of α-oxophosphonates (YC(O)P(O)(OR)_2_) and dialkyl phosphites or diarylphosphine oxides depended on the Y substituent of the oxo-compound, the nature of the P-reagent and the amount of the diethylamine catalyst. Starting from dimethyl α-oxoethylphosphonate, in the presence of 5% of diethylamine, the corresponding Pudovik adduct was the single product. While using 40% of the catalyst, the rearranged species with the >P(O)–O–CH–P(O)< skeleton was the exclusive component. A similar reaction of α-oxobenzylphosphonate followed the rearrangement protocol. X-ray crystallography revealed not only the spatial structures of the three products, but also an intricate pattern evolving from the interplay of slight chemical differences, solvent inclusion and disorder as well as *H*-bridge patterns, which invite further investigation. In vitro activity of the compounds was assessed on different tumor cell cultures using end-point-type cell tetrazolium-based measurements. These structure–activity studies revealed a cytostatic effect for four rearranged derivatives containing aromatic units. One of them had a pronounced effect on MDA-MB 231 and Ebc-1 cells, showing IC_50_ = 37.8 and 25.9 µM, respectively.

## 1. Introduction

Tetraalkyl methylenebisphosphonates and related derivatives are important intermediates, e.g., they may be modified by substitution on the central carbon atom. A number of methods were elaborated for alkylation [1,2,3,4,5,6,7,8] and acylation [9]. On the other hand, substituted hydroxy-methylenebisphosphonic derivatives form a prominent group called dronates that are used for bone diseases such as osteoporosis and cancer [10,11,12]. Dronic acid derivatives may be synthesized by the reaction of substituted acetic acids with phosphorus trichloride or phosphorous acid in solvents like methanesulfonic acid or sulfolane [13,14,15]. The senior author of this paper with colleagues was the one who elaborated the optimized synthesis of alendronate [16,17], ibandronate [16,17], risedronate [16,18] and zoledronate [16,18]. According to this, if methanesulfonic acid is the solvent, 3.2 equivalents of phosphorus trichloride should be used as the reagent; if sulfolane serves as the medium, phosphorus trichloride and phosphorous acid should be applied in a ratio of 2:2. It was a noteworthy observation that ionic liquid additives promoted the efficiency of the reactions [19,20,21,22]. The other possibility for the preparation of dronic acid derivatives involved the addition of dialkyl phosphites to α-oxophosphonates [23,24,25,26,27,28,29], which is called the Pudovik reaction. In this article, we aimed at the synthesis of methylenebisphosphonic derivatives with mixed P-functions involving a phosphine oxide moiety. We also explored the rearrangement side-reaction, which afforded products with a >P(O)–O–CH–P(O)< moiety.

## 2. Results and Discussion

### 2.1. Synthesis

In the first series of experiments, dimethyl α-oxoethylphosphonate (**1**) was reacted with dimethyl phosphite at 0 °C in diethyl ether for 8 h. The outcome depended on the quantity of the diethylamine (DEA) catalyst applied: using 5%, the Pudovik reaction took place selectively to afford α-hydroxy-methylenebisphosphonate **2a** (Table 1/Entry 1); however, in the presence of 40% of the catalyst, tetramethyl phosphonate-phosphate **3a**, formed by a rearrangement of the primary hydroxy-methylenebisphosphonate **2a**, was the exclusive product (Table 1/Entry 2). The addition of diethyl phosphite and dibutyl phosphite to oxophosphonate **1** applying 5% DEA also selectively provided the adducts **2b** and **2c**, respectively (Table 1/Entries 3 and 6). At the same time, after stirring the mixture in the presence of 40% of the catalyst at 0 °C for 8 h, the reaction mixtures comprised comparable portions of the adduct (**2**) and the rearranged product **3** (Table 1/Entries 4 and 7). In these cases, stirring at 26 °C for 3 days was necessary to achieve complete rearrangement (Table 1/Entries 5 and 8). It is noteworthy that the rearrangement of the phosphonate–phosphate compounds led to both possible isomers **3b-1**/**3b-2** and **3c-1**/**3c-2**.

In the next round, the secondary phosphine oxides diphenylphosphine oxide, bis(4-methylphenyl)phosphine oxide and bis(3,5-dimethylphenyl)phosphine oxide were added to the carbonyl group of dimethyl α-oxoethylphosphonate (**1**). After adding 40% of the catalyst, the reactions were completed after stirring at 0 °C for 8 h. In these cases, no rearranged products were formed (Table 1/Entries 9–11).

In summary, adducts **2a-c**, **2d-f**, along with rearranged products **3a**, **3b** and **3c** were obtained in 62–87% yields after column chromatography or recrystallization. All compounds were characterized by ^31^P, ^13^C and ^1^H NMR, as well as HRMS. Tetramethyl bisphosphonate **2a** and the rearranged version **3a** were described earlier [24,30].

Finally, diethyl α-oxobenzylphosphonate (**4**) was reacted with the three diarylphosphine oxides also used above. The results are summarized in Table 2. It was not possible to stop at the adduct stage as there was an increased inclination for the rearrangement. Carrying out the reaction in diethyl ether in the presence of 40% DEA at 0 °C for 8 h, only isomers **5-1** and **5-2** of the rearranged products **5(d-f)-1** and **5(d-f)-2** were formed. Compound **5d**, comprising isomers **5d-1** and **5d-2** in a comparable 6:4 proportion, was obtained as a mixture of isomers. However, the major isomers **5-1** of products **5e** and **5f** were prepared in a pure form by column chromatography. Compounds **5e-f** were fully characterized new species. Previously, it was also found that the adducts derived from α-oxobenzylphosphonates are less stable than those obtained from α-oxoethylphosphonates [29].

### 2.2. X-ray Structure of the Three Adducts

**Figure 1:**
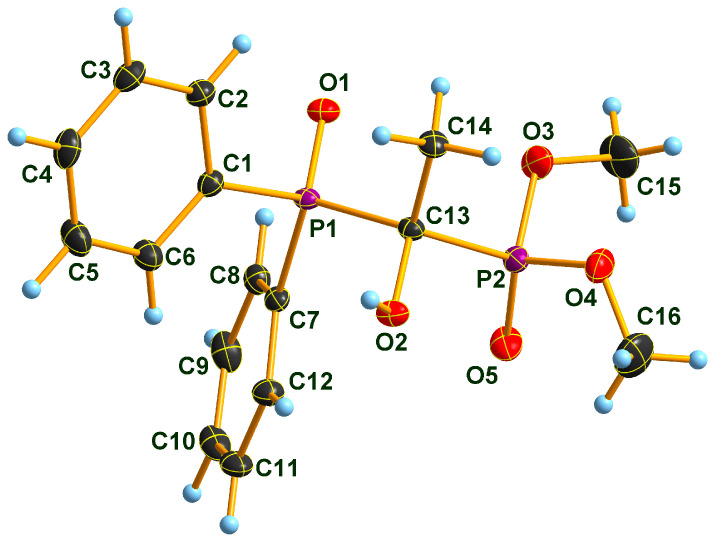
Molecular structure of hydroxymethylenephosphonate–phosphine oxide **2d** in the crystal. DIAMOND [31] representation; thermal ellipsoids are drawn at 50% probability level.

**Figure 2:**
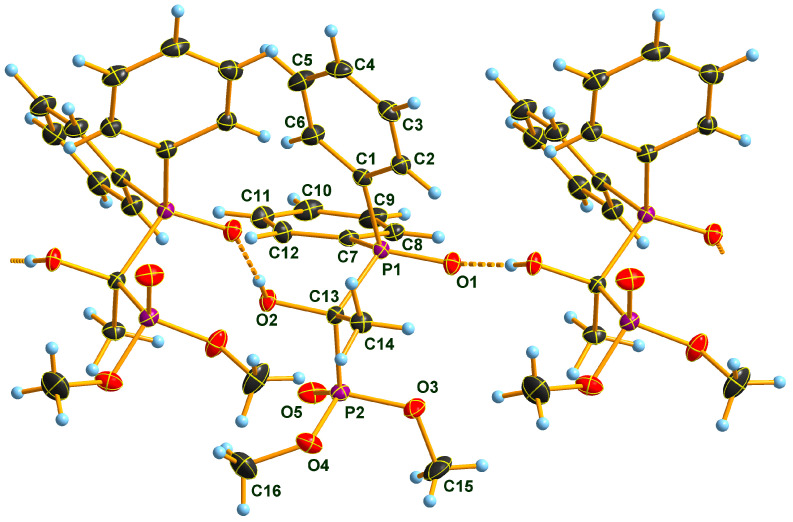
View of the hydrogen-bonded chain of molecules of **2d** in the crystal. DIAMOND [31] representation; thermal ellipsoids are drawn at 50% probability level. Symmetry code for the unlabeled molecules: *x*, 0.5 − *y*, 0.5 + *z* (left) and *x*, 0.5 − *y*, −0.5 + *z* (right).

**Figure 3:**
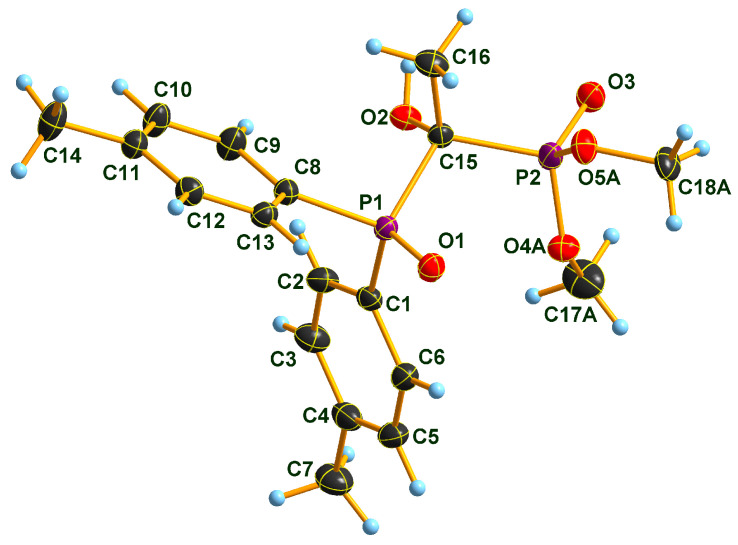
Molecular structure of phosphonate-phosphine oxide **2e.0.5C_3_H_6_O** in the crystal. The methoxy groups at P2 are disordered each over two positions. Only the major position is shown. The crystal structure contains one acetone molecule for every two molecules of **2e** (in a special position). The solvent molecule was omitted for clarity. DIAMOND [31] representation; thermal ellipsoids are drawn at 50% probability level.

**Figure 4:**
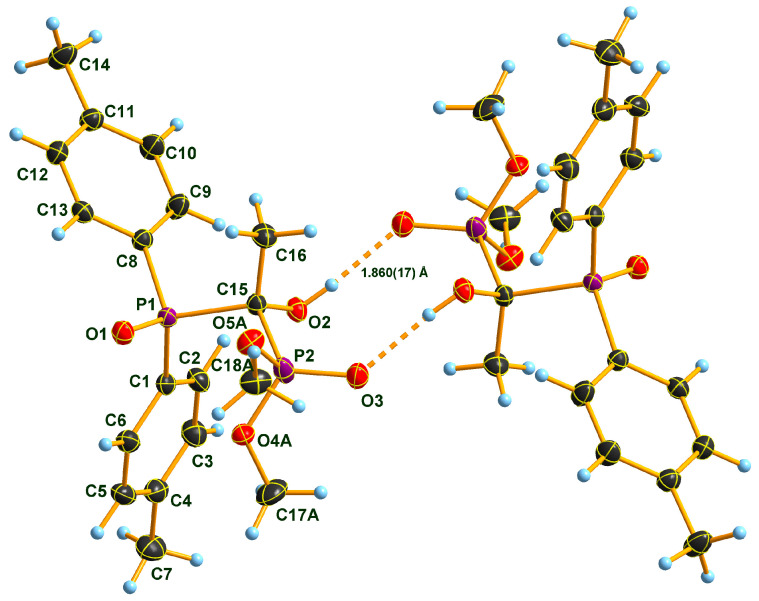
View of the hydrogen-bonded dimers of **2e.0.5C_3_H_6_O** in the crystal. Only major disorder methyl positions are shown while the acetone solvate is omitted from the drawing. DIAMOND [31] representation; thermal ellipsoids are drawn at 50% probability level. Symmetry code for the non-labeled molecule: 1.5 − *x*, 0.5 − *y*, −*z*.

**Figure 5:**
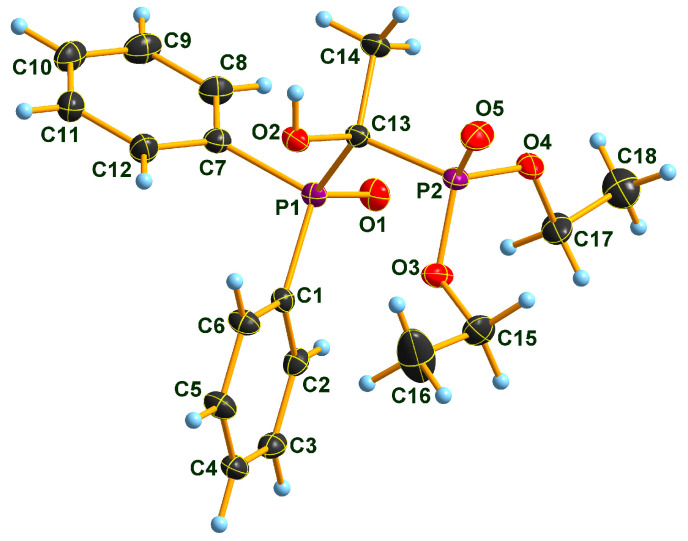
Molecular structure of hydroxymethylenephosphonate–phosphine oxide **6** in the crystal. DIAMOND [31] representation; thermal ellipsoids are drawn at 50% probability level.

**Figure 6:**
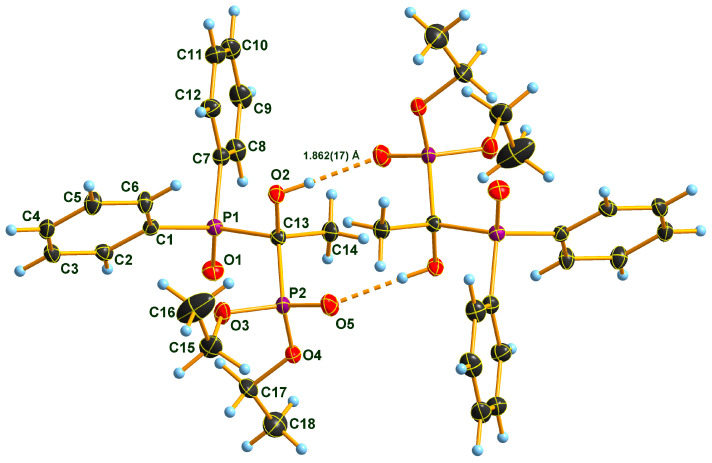
View of the hydrogen-bonded dimers around a symmetry center of **6** in the crystal. DIAMOND [31] representation; thermal ellipsoids are drawn at 50% probability level.

Two Pudovik adducts, dimethyl phosphonate–phosphine oxide derivatives **2d** and **2e** together the earlier described diethyl phosphonate–phosphine oxide **6** [29], were subjected to single crystal X-ray analysis. The results are presented in Figure 1, Figure 2, Figure 3, Figure 4, Figure 5 and Figure 6. The stereostructures of **2d**, **2e** and **6** are shown in Figure 1, Figure 3 and Figure 5, respectively, while selected geometries were included in Table 3. Connection of the molecules in the crystal structure can be seen in Figure 2, Figure 4 and Figure 6. It is clear, that **2d** is present as an *H*-bonded chain, while **2e** and **6** are *H*-bonded dimers.


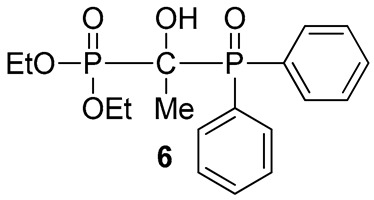


Bond distances in the P1 atom vicinity show nearly identical dimensions (Table 3). At first glance, this statement does not seem to apply to the P2 atom environment, inclining one to suppose that an eventual charge imbalance may be visible through slightly alternating bond lengths of phosphonates **2d**, **2e** and **6**. Such an assumption may eventually lead to the disparity of the *H*-bonding pattern difference between **2d** (continuous chain) and **2e** (discrete dimers). Nevertheless, this supposition may not really be supportable as the disorder in the methoxy groups of species **2e** obviates interpretations of bond differences in the immediate region of the P2 atom. One can only speculate that the disorder is a reason or a consequence of the dimer formation in the case of compound **2e**. It seems probable that a dimer-type of *H*-bridge buildup may be the consequence of the effect of the *p*-methyl substitution in the benzene ring, the solvent inclusion, and the disorder of the methoxy groups at P2. Hydroxy-methylenebisphosphonate **6** also forms *H*-bonded dimers in its crystal. As a very simple tool of assessing packing tightness in respect of **2d**, **2e.0.5 C_3_H_6_O** and **6**, it is instructive to compare their calculated densities in their crystals (See Experimental). The chain-forming **2d** had the highest value in this series, while **2e.0.5 C_3_H_6_O** had the lowest, even with the aid of a solvent molecule. It is also worth noting that the real assembly in the crystal had a perfect twofold symmetry rotor image with acetone solvent sitting on the symmetry axis. Thus, the real 2:1 stoichiometry is 2 × **2e.C_3_H_6_O**. The density of derivative **6** took on an intermediate value between the two others, thus suggesting that dimer formation tends to yield to looser packing.

### 2.3. Bioactivity of the Compounds Prepared

**Figure 7:**
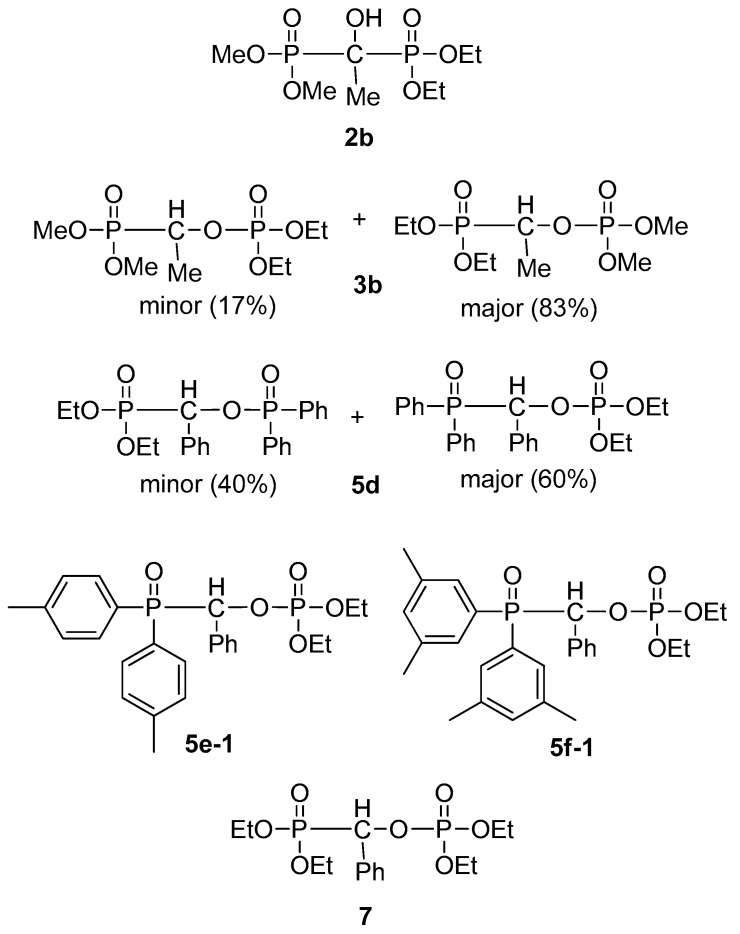
Members of the compound library showing significant cytotoxic activity.

Hydroxy-methylenebisphosphonic derivatives **2a–f** and **6** as well as related rearranged species **3a–c**, **5d**, **5e-1**, **5f-1** and **7** were subjected to bioactivity study. Phosphonate–phosphate **7** was described by us earlier [29]. From among the compounds investigated, the ones listed in Figure 7 (**2b**, **3b**, **5d**, **5e-1**, **5f-1** and **7**) showed significant activity.

In vitro cytostatic activity of the compounds was determined after an overnight treatment using 3-(4,5-dimethylthiazol-2-yl)-2,5-diphenyltetrazolium bromide (MTT) assay. (For the details see Experimental.) Cytostasis (%) and 50% inhibitory concentration (IC_50_) were determined for each compound on MDA-MB 231 human breast adenocarcinoma, A431 human epidermoid carcinoma, PC-3 human prostate adenocarcinoma, and Ebc-1 human lung squamous cell carcinoma cell lines.

Results indicated that several compounds elicited a cytostatic effect on the human tumor cell lines. Considering the percentage of cytostasis values at c = 50 µM, we concluded that compounds **3b**, **5f-1** and **7** had a moderate cytostatic effect on the cells. However, three compounds were more effective. Adduct **2b** was efficient on A431 human epidermoid carcinoma cells and showed a cytostasis = 49.9%. Phosphine oxide–phosphate **5d** induced a higher cytostasis on MDA-MB 231 human breast adenocarcinoma and Ebc-1 human lung carcinoma cells, showing a cytostasis of 48.9 and 45.3%, respectively, whereas **5e-1** elicited an outstanding effect on these cell lines: cytostasis = 69.9 and 72.4%, respectively). Overall, the MDA-MB 231 human breast adenocarcinoma cell line proved to be the most sensitive for all effective compounds. Cytostasis values at c = 50 µM are shown in Table 4. 

The above tendency was also confirmed when the effect was determined on the basis of the calculated IC_50_ values. While the effect of compounds **2a–f** and **3a–c** were not very efficient, a higher cytostatic effect was observed for compounds **5d**, **5e-1**, **5f-1**, and **7**. Compounds **5f-1** and **7** had a lower cytostatic effect (IC_50_ = 99.5–115.8 µM), whereas **5e-1** induced a more pronounced cytostasis on A431 cells (IC_50_ = 40.4 µM). The most effective on MDA–MB 231 and Ebc-1 cells (IC_50_ = 37.8 and 25.9 µM, respectively) proved to be **5e-1.** The IC_50_ values of the compounds are summarized in Table 5. Daunomycin, an often-used reference compound, was also tested on the cell lines we applied. Its efficiency was much better [32,33] than that of our compounds. For details, see footnote “a” in Table 5.

## 3. Experimental

### 3.1. General

The ^31^P, ^13^C, ^1^H-NMR spectra were taken on a Bruker DRX-500 or Bruker Avance-300 spectrometer operating at 202.4, 125.7, and 500 MHz or 121.5, 75.4 and 300 MHz respectively. The couplings were given in Hz. LC–MS measurements were performed with an Agilent 1200 liquid chromatography system, coupled with a 6130 quadrupole mass spectrometer equipped with an ESI ion source (Agilent Technologies, Palo Alto, CA, USA). High-resolution mass spectrometric measurements were performed using a Thermo Velos Pro Orbitrap Elite hybrid mass spectrometer in positive electrospray mode.

### 3.2. General Procedure for the Synthesis of Tetraalkyl α-Hydroxy-ethylidenebisphosphonates

2.2 mmol (0.33 g) of dimethyl α-oxoethylphosphonate was added dropwise to a mixture of 2.2 mmol dialkyl phosphite (dimethyl phosphite: 0.20 mL, diethyl phosphite: 0.30 mL, dibutyl phosphite: 0.43 mL) and 0.11 mmol (0.010 mL) of diethylamine in diethyl ether (13 mL) at 0 °C on stirring. After an 8 h reaction time, the solvent was evaporated, and the crude product obtained was purified by column chromatography (using DCM–MeOH 97:3 as the eluent on silica gel).

#### 3.2.1. Tetramethyl α-Hydroxy-ethylidenebisphosphonates (**2a**)

Yield: 0.40 g (68%), ^31^P NMR (CDCl_3_) δ 22.3 Ref [24] δ_P_ 22.0; ^13^C NMR (CDCl_3_) δ 20.0 (t, *J* = 2.2 Hz, C*C*H_3_), 54.2 and 54.3 (t, *J* = 3.5 Hz, 4 OCH_3_), 71.7 (t, *J* = 156.8 Hz, *C*CH_3_); ^1^H NMR (CDCl_3_) δ 1.70 (t, *J* = 16.2 Hz, 3H, CCH_3_), 3.88–3.93 (m, 12H, OCH_3_); [M + H]^+^ = 263; [M + Na]^+^_found_ = 285.0272; C_6_H_16_O_7_P_2_Na required 285.0269.

#### 3.2.2. Diethyl–Dimethyl α-Hydroxy-ethylidenebisphosphonate (**2b**)

Yield: 0.51 g (80%), ^31^P NMR (CDCl_3_) δ_P1_ 19.9 and δ_P2_ 22.7 (d, ^2^*J*_PP_ = 40.1 Hz), Ref [25] δ_P1_ 20.6 and δ_P2_ 23.4 (^2^*J*_PP_ = 39.3 Hz); ^13^C NMR (CDCl_3_) δ 16.4 (d, *J* = 5.6 Hz, 2 CH_2_*C*H_3_), 20.4 (s, C*C*H_3_), 54.2 and 54.3 (d, *J* = 7.1 Hz, 2 OCH_3_), 63.7 and 63.8 (d, *J* = 4.9 Hz, 2 OCH_2_), 71.6 (t, *J* = 156.4 Hz, *C*CH_3_); ^1^H NMR (CDCl_3_) δ 1.37 (t, *J* = 7.0 Hz, 6H, CH_2_C*H_3_*), 1.68 (t, *J* = 16.1 Hz, 3H, CCH_3_), 3.88 (d, *J* = 10.5 Hz, 6H, OCH_3_), 4.21–4.32 (m, 4H, OCH_2_); [M + H]^+^ = 291; [M + Na]^+^_found_ = 313.0573; C_8_H_20_O_7_P_2_Na required 313.0582. 

#### 3.2.3. Dibutyl-Dimethyl α-Hydroxy-ethylidenebisphosphonate (**2c**)

Yield: 0.50 g (66%) ^31^P NMR (CDCl_3_) δ_P1_ 19.8 and δ_P2_ 22.8 (d, ^2^*J*_PP_ = 39.3 Hz), Ref [30] δ_P1_ 20.4 and δ_P2_ 23.3 (^2^*J*_PP_ = 40.1 Hz); ^13^C NMR (CDCl_3_) δ 13.6 (s, 2 CH_2_*C*H_3_), 18.6 (s, 2 *C*H_2_CH_3_), 20.1 (s, C*C*H_3_), 32.5 (d, *J* = 5.5 Hz, 2 OCH_2_*C*H_2_), 54.0–54.1 and 54.2–54.3 (m, 2 OCH_3_), 67.4 (m, 2 OCH_2_), 71.7 (t, *J* = 156.1 Hz, *C*CH_3_); ^1^H NMR (CDCl_3_) δ 0.96 (t, *J* = 7.4 Hz, 6H, 2 CH_2_C*H_3_*), 1.38–1.50 (m, 4H, C*H_2_*CH_3_), 1.63–1.78 (m, 3 + 4H, CCH_3_ + OCH_2_C*H_2_*), 3.89 (dd, *J*_1_ = 10.5 Hz, *J*_2_ = 1.6 Hz, 6H, OCH_3_), 4.16–4.23 (m, 4H, OCH_2_); [M + H]^+^ = 347; [M + Na]^+^_found_ = 369.1209; C_12_H_28_O_7_P_2_Na required 369.1208.

### 3.3. General Procedure for the Synthesis of Dimethyl 1-Diarylphosphinoyl-1-hydroxy-ethylphosphonate

2.2 mmol (0.33 g) of dimethyl α-oxoethylphosphonate was added dropwise to a mixture of 2.2 mmol diarylphosphine oxide (diphenylphosphine oxide: 0.44 g, bis(4-methylphenyl)phosphine oxide: 0.50 g, bis(3,5-dimethylphenyl)phosphine oxide: 0.56 g) and 0.88 mmol (0.090 mL) of diethylamine in diethyl ether (13 mL) at 0 °C on stirring. After an 8 h reaction time, the precipitated material was removed by filtration, washed with diethyl ether, and the residue recrystallized from acetone. The products were white crystalline compounds.

#### 3.3.1. Dimethyl 1-Diphenylphosphinoyl-1-hydroxy-ethylphosphonate (**2d**)

Yield: 0.50 g (64%), mp: 131–132 °C; ^31^P NMR (CDCl_3_) δ_P1_ 23.9 and δ_P2_ 29.0 (d, ^2^*J*_PP_ = 25.4 Hz); ^13^C NMR (CDCl_3_) δ 20.3 (s, C*C*H_3_), 53.9 and 54.0 (d, *J* = 7.4 Hz, 2 OCH_3_), 74.8 (dd, *J*_1_ = 154.6 Hz, *J*_2_ = 79.0 Hz, *C*CH_3_), 127.8 and 128.2 (d, *J* = 11.7 Hz, 2 Cγ), 130.4 (dd, *J*_1_ = 96.9 Hz, *J*_2_ = 5.5 Hz, C_α_), 130.8 (d, *J* = 98.1 Hz, C_α_), 131.6 and 131.8 (d, *J* = 2.8 Hz, 2 C_δ_), 132.4 and 132.7 (d, *J* = 8.6 Hz, 2 C_β_); ^1^H NMR (CDCl_3_) δ 1.65 (t, *J* = 15.6 Hz, 3H, CCH_3_), 3.44 and 3.70 (d, *J* = 10.6 Hz, 6H, OCH_3_), 7.40–7.61 (m, 6H, ArH), 8.09 and 8.18 (dd, *J*_1_ = 11.1 Hz, *J*_2_ = 6.9 Hz, 4H, ArH_β_); [M + H]^+^ = 355; [M + Na]^+^_found_ = 377.0681; C_16_H_20_O_5_P_2_Na required 377.0684.

#### 3.3.2. Dimethyl 1-Bis(4-methylphenyl)phosphinoyl-1-hydroxy-ethylphosphonate (**2e**)

Yield: 0.52 g (62%), mp: 153–154 °C; ^31^P NMR (CDCl_3_) δ_P1_ 24.0 and δ_P2_ 30.2 (d, ^2^*J*_PP_ = 29.0 Hz); ^13^C NMR (CDCl_3_) δ 20.6 (s, C*C*H_3_), 21.5 (s, 2 ArCH_3_), 53.9 and 54.0 (d, *J* = 7.4 Hz, 2 OCH_3_), 74.6 (dd, *J*_1_ = 153.7 Hz, *J*_2_ = 76.3 Hz, *C*CH_3_), 127.1 (dd, *J*_1_ = 99.4 Hz, *J*_2_ = 4.9 Hz, C_α_), 127.2 (d, *J* = 100.9 Hz, C_α_), 128.7 and 128.9 (d, *J* = 12.1 Hz, 2 Cγ), 132.4 and 132.7 (d, *J* = 9.1 Hz, 2 C_β_), 142.1 and 142.3 (d, *J* = 2.9 Hz, 2 C_δ_); ^1^H NMR (CDCl_3_) δ 1.61 (t, *J* = 15.4 Hz, 3H, CCH_3_), 2.39 (s, 6H, ArCH_3_), 3.55 and 3.67 (d, *J* = 10.6 Hz, 6H, OCH_3_), 7.27–7.29 (m, 4H, ArH), 7.92 and 8.03 (dd, *J*_1_ = 11.0 Hz, *J*_2_ = 8.0 Hz, 4H, ArH_β_); [M + H]^+^ = 383; [M + Na]^+^_found_ = 405.1003; C_18_H_24_O_5_P_2_Na required 405.0997.

#### 3.3.3. Dimethyl 1-Bis(3,5-dimethylphenyl)phosphinoyl-1-hydroxy-ethylphosphonate (**2f**)

Yield: 0.62 g (69%), mp: 161–162 °C; ^31^P NMR (CDCl_3_) δ_P1_ 24.3 and δ_P2_ 30.0 (d, ^2^*J*_PP_ = 29.0 Hz); ^13^C NMR (CDCl_3_) δ 20.7 (s, C*C*H_3_), 21.3 (s, 4 ArCH_3_), 53.8 and 54.0 (d, *J* = 7.4 Hz, 2 OCH_3_), 74.7 (dd, *J*_1_ = 153.6 Hz, *J*_2_ = 76.3 Hz, *C*CH_3_), 129.9 and 130.1 (d, *J* = 8.7 Hz, 2 C_β_) 130.2 (dd, *J*_1_ = 95.7 Hz, *J*_2_ = 5.1 Hz, C_α_), 130.5 (d, *J* = 96.0 Hz, C_α_), 133.4 and 133.5 (d, *J* = 3.0 Hz, 2 C_δ_), 137.4 and 137.7 (d, *J* = 12.4 Hz, 2 C_γ_); ^1^H NMR (CDCl_3_) δ 1.65 (t, *J* = 14.7 Hz, 3H, CCH_3_), 2.36 (d, *J* = 5.5 Hz, 12H, ArCH_3_), 3.52 and 3.70 (d, *J* = 10.6 Hz, 6H, OCH_3_), 7.14 (s, 2H, ArH_δ_), 7.67 and 7.77 (d, *J* = 11.3 Hz, 4H, ArH_β_); [M + H]^+^ = 411; [M + Na]^+^_found_ = 433.1312; C_20_H_28_O_5_P_2_Na required 433.1310.

### 3.4. General Procedure for the Synthesis of Dialkyl 1-(Dialkylphosphonoylethyl)phosphate

2.2 mmol (0.33 g) of dimethyl α-oxoethylphosphonate was added dropwise to a mixture of 2.2 mmol dialkyl phosphite (dimethyl phosphite: 0.20 mL, diethyl phosphite: 0.30 mL, dibutyl phosphite: 0.43 mL) and 0.88 mmol (0.090 mL) of diethylamine in diethyl ether (13 mL) at 0 °C on stirring. After 8–72 h reaction time, the solvent was evaporated and the crude product obtained was purified by column chromatography (using DCM–MeOH 97:3 as the eluent on silica gel).

#### 3.4.1. Dimethyl 1-(Dimethylphosphonoylethyl)phosphate (**3a**)

Yield: 0.43 g (75%), ^31^P NMR (CDCl_3_) δ_P1_ 1.1 and δ_P2_ 22.5 (d, ^3^*J*_PP_ = 30.1 Hz), Ref [34] δ_P1_ 0.4 and δ_P2_ 21.9 (d, ^3^*J*_PP_ = 29.3 Hz); ^13^C NMR (CDCl_3_) δ 16.6 (s, C*C*H_3_), 53.4 and 53.6 (dd, *J*_1_ = 6.8 Hz, *J*_2_ = 3.8 Hz, 2 OCH_3_); 54.4 and 54.6 (dd, *J*_1_ = 6.3 Hz, *J*_2_ = 3.6 Hz, 2 OCH_3_), 69.1 (dd, *J*_1_ = 174.1 Hz, *J*_2_ = 6.9 Hz, CH); ^1^H NMR (CDCl_3_) δ 1.61 (dd, *J*_1_ = 16.7 Hz, *J*_2_ = 7.1 Hz, 3H, CCH_3_), 3.77–3.84 (m, 12H, OCH_3_), 4.63–4.91 (m, 1H, CH); [M + H]^+^ = 263; [M + Na]^+^_found_ = 285.0268; C_6_H_16_O_7_P_2_Na required 285.0269.

#### 3.4.2. Dimethyl 1-(Diethylphosphonoylethyl)phosphate (**3b-1**) and Diethyl 1-(Dimethylphosphonoylethyl)phosphate (**3b-2**)

Yield: 0.56 g (87%), major (83%): ^31^P NMR (CDCl_3_) δ_P1_ 1.0 and δ_P2_ 20.0 (^3^*J*_PP_ = 31.3 Hz); ^13^C NMR (CDCl_3_) δ 16.38 and 16.43 (d, *J* = 5.5 Hz, 2 CH_2_*C*H_3_), 16.6 (s, CCH_3_), 54.4 and 54.5 (d, *J* = 6.2 Hz, 2 OCH_3_), 63.0 and 63.1 (d, *J* = 6.5 Hz, 2 OCH_2_), 69.4 (dd, *J*_1_ = 174.5 Hz, *J*_2_ = 6.8 Hz, CH); ^1^H NMR (CDCl_3_) δ 1.32 (t, *J* = 7.0 Hz, 6H, CH_2_C*H_3_*), 1.54 (dd, *J*_1_ = 16.7 Hz, *J*_2_ = 7.0 Hz, 3H, CCH_3_), 3.75 and 3.77 (d, *J* = 11.5 Hz, 6H, OCH_3_), 4.13–4.20 (m, 4H, C*H_2_*CH_3_); 4.62–4.72 (m, 1H, CH); minor (17%): δ_P1_ −1.3 and δ_P2_ 22.6 (^3^*J*_PP_ = 31.0 Hz); ^13^C NMR (CDCl_3_) δ 16.0 (d, *J* = 6.8 Hz, 2 CH_2_*C*H_3_), 16.6 (s, C*C*H_3_), 53.4 and 53.6 (d, *J* = 6.5 Hz, 2 OCH_3_), 64.1 and 64.2 (d, *J* = 6.1 Hz, 2 OCH_2_), 68.8 (dd, *J*_1_ = 174.1 Hz, *J*_2_ = 7.0 Hz, CH); ^1^H NMR (CDCl_3_) δ 3.80 and 3.81 (d, *J* = 10.7 Hz, 6H, OCH_3_). The other signals were common with those of the major isomer; [M + H]^+^ = 291; [M + Na]^+^_found_ = 313.0581; C_8_H_20_O_7_P_2_Na required 313.0582.

#### 3.4.3. Dimethyl 1-(Dibutylphosphonoylethyl)phosphate (**3c-1**) and Dibutyl 1-(Dimethylphosphonoylethyl)phosphate (**3c-2**)

Yield: 0.53 g (70%), major (81%) ^31^P NMR (CDCl_3_) δ_P1_ 1.1 and δ_P2_ 20.0 (^3^*J*_PP_ = 31.6 Hz); ^13^C NMR (CDCl_3_) δ 13.5 (s, 2 CH_2_*C*H_3_), 16.7 (s, C*C*H_3_), 18.6 (s, 2 *C*H_2_CH_3_), 32.5 and 32.6 (d, *J* = 3.2 Hz, 2 OCH_2_*C*H_2_), 54.3–54.4 and 54.5–54.6 (m, 2 OCH_3_), 66.4–66.9 (m, 2 OCH_2_), 69.5 (dd, *J*_1_ = 174.6 Hz, *J*_2_ = 6.9 Hz, CH); ^1^H NMR (CDCl_3_) δ 0.96 (t, *J* = 7.9 Hz, 6H, CH_2_C*H*_3_), 1.40–1.47 (m, 4H, C*H*_2_CH_3_), 1.60 (dd, *J*_1_ = 16.5 Hz, *J*_2_ = 7.1 Hz, 3H, CCH_3_), 1.67–1.72 (m, 4H, OCH_2_C*H_2_*), 3.80 and 3.83 (d, *J* = 11.5 Hz, 6H, OCH_3_), 4.04–4.20 (m, 4H, OCH_2_), 4.66–4.78 (m, 1H, CH); minor (19%) ^31^P NMR (CDCl_3_) δ_P1_ –0.9 and δ_P2_ 22.8 (^3^*J*_PP_ = 31.4 Hz); ^13^C NMR (CDCl_3_) δ 68.8 (dd, *J*_1_ = 174.1 Hz, *J*_2_ = 7.1 Hz, CH). The other signals are common with those of the major isomer; ^1^H NMR (CDCl_3_) δ 3.85 and 3.86 (d, *J* = 10.5 Hz, 6H, OCH_3_). The other signals are common with those of the major isomer; [M + H]^+^ = 347; [M + Na]^+^_found_ = 369.1201; C_12_H_28_O_7_P_2_Na required 369.1208.

### 3.5. General Procedure for Diethyl (Diarylphosphinoyloxybenzyl)phosphonate and Diethyl (Diarylphosphinoylbenzyl)phosphate

1.5 mmol (0.36 g) of diethyl α-oxobenzylphosphonate was added slowly to a mixture of 1.5 mmol (bis(4-methylphenyl)phosphine oxide: 0.35 g, bis(3,5-dimethylphenyl)phosphine oxide: 0.40 g) and 0.60 mmol (0.060 mL) of diethylamine in diethyl ether (13 mL) at 0 °C on stirring. After an 8 h reaction time, the solvent was evaporated, and the crude product obtained was purified with column chromatography (using ethyl acetate as the eluent on silica gel).

#### 3.5.1. Diethyl (Diphenylphosphinoylbenzyl)phosphate (**5d-1**) and Diethyl (Diphenylphosphinoyloxybenzyl)phosphonate (**5d-2**)

Yield: 0.47 g (70%), major (60%): ^31^P NMR (CDCl_3_) δ_P1_ –1.5 and δ_P2_ 28.6 (^3^*J*_PP_ = 31.3 Hz); ^13^C NMR (CDCl_3_) δ 15.6 and 15.8 (d, *J* = 7.4 Hz, 2 CH_2_*C*H_3_), 63.8 and 63.9 (d, *J* = 6.0 Hz, 2 OCH_2_), 77.4 (dd, *J*_1_ = 85.7 Hz, *J*_2_ = 7.9 Hz, CH). The aromatic range was rather complex between δ 128.0–132.6; ^1^H NMR (CDCl_3_) δ 0.90 and 0.96 (t, *J* = 7.1 Hz, 6H, CH_2_C*H_3_*), 3.41–3.70 (m, 4H, OCH_2_), 6.06 (dd, *J*_1_ = 9.7 Hz, *J*_2_ = 4.4 Hz, 1H, CH), aromatic region: 7.15–7.98 (m, 15H, ArH); minor (40%): ^31^P NMR (CDCl_3_) δ_P1_ 17.2 and δ_P2_ 34.7 (^3^*J*_PP_ = 26.7 Hz); ^13^C NMR (CDCl_3_) δ 16.2 and 16.3 (d, *J* = 5.8 Hz, 2 CH_2_*C*H_3_), 63.3 and 63.5 (d, *J* = 6.9 Hz, 2 OCH_2_), 72.0 (dd, *J*_1_ = 172.6 Hz, *J*_2_ = 7.0 Hz, CH). The aromatic range was rather complex between δ 128.0–132.6; ^1^H NMR (CDCl_3_) δ 1.09 and 1.18 (t, *J* = 7.1 Hz, 6H, CH_2_C*H_3_*), 3.78–4.15 (m, 4H, OCH_2_), 5.63 (dd, *J*_1_ = 13.5 Hz, *J*_2_ = 11.2 Hz, 1H, CH), aromatic region: 7.15–7.98 (m, 15H, ArH); [M + H]^+^ = 445; [M + Na]^+^_found_ = 467.1154; C_23_H_26_O_5_P_2_Na required 467.1153.

#### 3.5.2. Diethyl 1-Bis((4-methylphenyl)phosphinoylbenzyl)phosphate (**5e-1**)

Yield: 0.40 g (65%), ^31^P NMR (CDCl_3_) δ_P1_ –1.3 and δ_P2_ 29.0 (^3^*J*_PP_ = 31.4 Hz); ^13^C NMR (CDCl_3_) δ 15.6 and 15.7 (d, *J* = 7.4 Hz, 2 CH_2_*C*H_3_), 21.6 (d, *J* = 9.8 Hz, 2 ArCH_3_), 63.8 and 63.9 (d, *J* = 5.9 Hz, 2 OCH_2_), 77.6 (dd, *J*_1_ = 85.3 Hz, *J*_2_ = 8.0 Hz, CH). The aromatic range was rather complex between δ 124.6–142.9; ^1^H NMR (CDCl_3_) δ 0.94 and 1.00 (t, *J* = 7.3 Hz, 6H, CH_2_C*H_3_*), 2.35 and 2.42 (s, 6H, ArCH_3_), 3.46–3.74 (m, OCH_2_), 6.03 (dd, *J*_1_ = 9.8 Hz, *J*_2_ = 4.5 Hz, 1H, CH), aromatic region: 7.18–7.33 (m, 9H, ArH), 7.55 and 7.83 (dd, *J*_1_ = 11.1 Hz *J*_2_ = 8.1 Hz, 4H, ArH_β_); [M + H]^+^ = 473; [M + Na]^+^_found_ = 495.1467; C_25_H_30_O_5_P_2_Na required 495.1466.

#### 3.5.3. Diethyl 1-Bis((3,5-dimethylphenyl)phosphinoylbenzyl)phosphate (**5f-1**)

Yield: 0.42 g (72%), ^31^P NMR (CDCl_3_) δ_P1_ −1.2 and δ_P2_ 29.1 (^3^*J*_PP_ = 30.9 Hz); ^13^C NMR (CDCl_3_) δ 15.6 and 15.8 (d, *J* = 7.5 Hz, 2 CH_2_*C*H_3_), 21.2 (d, *J* = 13.3 Hz, 4 ArCH_3_), 63.6 and 63.8 (d, *J* = 5.9 Hz, 2 OCH_2_), 77.4 (dd, *J*_1_ = 84.8 Hz, *J*_2_ = 8.0 Hz, CH). The aromatic range was rather complex between δ 128.0–138.2; ^1^H NMR (CDCl_3_) δ 0.95 and 1.04 (t, *J* = 7.4 Hz, 6H, CH_2_C*H*_3_), 2.27 and 2.40 (s, 12H, ArCH_3_), 3.48–3.76 (m, 4H, OCH_2_), 6.06 (dd, *J*_1_ = 9.7 Hz, *J*_2_ = 3.1 Hz, 1H, CH), aromatic region: 7.22–7.34 (m, 9H, ArH), 7.61 (d, *J* = 11.7 Hz, 2H, ArH_β_); [M + H]^+^ = 501; [M + Na]^+^_found_ = 523.1771; C_27_H_34_O_5_P_2_Na required: 523.1779.

For the ^31^P, ^13^C and ^1^H NMR spectra of the compounds prepared see Supplementary Materials.

### 3.6. Single Crystal X-ray Diffraction Studies

Single crystals of compound **2d**, **2e.0.5 C_3_H_6_O** and **6** suitable for X-ray diffraction were obtained by slow evaporation of the respective acetone solution. The crystals were introduced into perfluorinated oil and a suitable single crystal was carefully mounted on the top of a thin glass wire. Data collection was performed with an Oxford Xcalibur 3 diffractometer equipped with a Spellman generator (50 kV, 40 mA) and a Kappa CCD detector, operating with Mo-K_α_ radiation (λ = 0.71071 Ǻ).

Data collection and reduction were performed using CrysAlisPro software [35]. Absorption correction using the multiscan method [35] was applied. The structures were solved with SHELXS-97 [36], refined with SHELXL-97 [37] and finally checked using PLATON [38]. Details of the data collection and structure refinement are summarized in Table 6.

CCDC-2281416, CCDC-2281417 and CCDC-2281418 contain supplementary crystallographic data for compounds **2d**, **2e·0.5C_3_H_6_O** and **6**, respectively. These data can be obtained free of charge from The Cambridge Crystallographic Data Centre via www.ccdc.cam.ac.uk/data_request/cif (accessed on 13 July 2023).

### 3.7. In Vitro Cytotstasis Assays 

#### Cell Lines and Culture Conditions

The in vitro cytostatic effect of the compounds was studied on MDA-MB 231 human breast adenocarcinoma [39], A431 human epidermoid carcinoma [40], PC-3 human prostate adenocarcinoma [41], and Ebc-1 human lung squamous cell carcinoma [42] cell lines. Cells were cultured in a DMEM medium supplemented with 10% FBS, 2 mM L-glutamine, penicillin–streptomycin antibiotic mixture (50 IU/mL and 50 μg/mL, respectively), 1 mM sodium pyruvate and 1% non-essential amino acid mixture. The cell cultures were maintained at 37 °C in a humidified atmosphere with 5% CO_2_. The cells were grown to confluent state and then they were harvested by trypsinization and divided into 96-well tissue culture plates (initial cell number was of 5.0 × 10^3^ cells/well). Cells were allowed to attach for 24 h at 37 °C when the culturing medium was removed and they were treated with the compounds in 2, 10, 50, and 250 μM concentration in a serum-free medium. (The treating solutions contained 1.0 *v*/*v*% DMSO). Control cells were treated only with serum-free medium or with DMSO (c = 1.0 *v*/*v*%) under the same conditions. After overnight incubation, cells were washed twice with a serum-free medium, and then cultured for another 72 h in 10% serum-containing medium at 37 °C. An MTT-solution (at c = 0.37 mg/mL final concentration) was added to each well and incubated for 3 h. The cells were centrifuged for 5 min at 900 g, and then the supernatant was removed. The obtained formazan crystals were dissolved in DMSO (100 µL) and the optical density (OD) of the samples was measured with an ELISA Reader (iEMS Reader, Labsystems, Vantaa, Finland) at detecting wavelength = 540 and reference wavelength = 620 nm. OD620 values were subtracted from the OD540 values, and then cytostasis % was calculated from this corrected OD value by the following equation:

Cytostatic effect (%) = [1 − (OD_treated_/OD_control_)] × 100

where OD_treated_ and OD_control_ correspond to the optical densities of the treated and control wells, respectively. In each case, two independent experiments were carried out with 4 parallel measurements. Statistical data analysis was performed using Student’s *t*-test at a 95% confidence level. A 50% inhibitory concentration (IC_50_, expressed in micromolar units) was determined from the dose–response curves: cytostasis was plotted as a function of concentration on which a sigmoidal curve was fitted using Microcal™ Origin 2018 software [43,44].

## 4. Conclusions

The outcome of the reaction of α-oxophosphonates (ZC(O)P(O)(OR)_2_) and Y_2_P(O)H reagents depended on the nature of the Z substituent of the oxo-compound, the Y substituent of the P-reagent, and the amount of the diethylamine catalyst. In case of Z = Me, new hydroxy-methylenebisphosphonic derivatives with different P-functions were synthesized. Performing the reactions in the presence of an increased amount (40%) of the catalyst, or starting from an α-oxobenzylphosphnate, rearranged species comprising the >P(O)–O–CH–P(O)< motif were the products. The molecular dimensions mostly conformed to those expected for this kind of P-compound. The intermolecular connection pattern may be realized in centrosymmetric *H*-bridge dimers, but in one case a catameric chain structure was experienced. Solvent inclusion as well as the presence of disorder were also present in one of the crystals hampering deeper insight into the solid-state relations. Part of the compounds we synthesized showed significant in vitro cytotoxic activity on human tumor cell cultures of different tissue origin. The rearranged derivatives with aromatic units possessed considerable antiproliferative activity characterized by low IC_50_ values.

## Figures and Tables

**Table 1:** The reaction of dimethyl α-oxoethylphosphonate (**1**) with dialkyl phosphites or secondary phosphine oxides under different conditions.

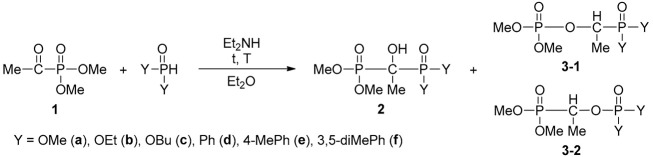								
Entry	Y	Catalyst (%)	T (°C)	t	Product Composition (%) ^[a][b]^			Yield (%)
					2	3-1	3-2	
1	MeO	5	0	8 h	100	–		68 (**2a**)
2	MeO	40	0	8 h	–	100		75 (**3a**)
3	EtO	5	0	8 h	100	–		80 (**2b**)
4	EtO	40	0	8 h	60	32	8	–
5	EtO	40	26	3 days	–	83	17	87 (**3b**)
6	BuO	5	0	8 h	100	–		66 (**2c**)
7	BuO	40	0	8 h	50	34	16	–
8	BuO	40	26	3 days	–	81	19	70 (**3c**)
9	Ph	40	0	8 h	100	–	–	64 (**2d**)
10	4-MePh	40	0	8 h	100	–	–	62 (**2e**)
11	3,5-diMePh	40	0	8 h	100	–	–	69 (**2f**)

^[a]^ On the basis of relative ^31^P NMR intensities. ^[b]^ The data set shaded in yellow refer to the best experiments.

**Table 2:** The reaction of diethyl α-oxobenzylphosphonate (**4**) with secondary phosphine oxides.

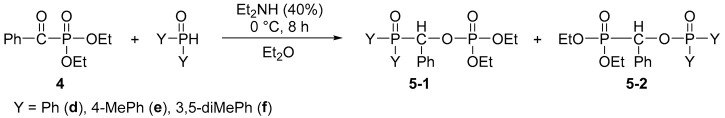				
Entry	Y	Product Composition (%) ^[a][b]^		Yield (%)
		5-1	5-2	
1	Ph	60	40	70 (**5d-1** + **5d-2**)
2	4-MePh	88	12	65 (**5e-1**)
3	3,5-diMePh	77	23	72 (**5f-1**)

^[a]^ On the basis of relative ^31^P NMR intensities. ^[b]^ The data set shaded in yellow refer to the best experiments.

**Table 3:** Selected bond lengths (Å) of compound **2d**, **2e.0.5C_3_H_6_O** and **6**.

2d		2e		6	
P1–O1	P1–O1	1.485 (1)	1.493 (1)	P1–O1	1.488 (1)
P1–C7	P1–C8	1.809 (2)	1.801 (2)	P1–C7	1.807 (1)
P1–C1	P1–C1	1.809 (2)	1.811 (2)	P1–C1	1.809 (1)
P1–C13	P1–C15	1.862 (2)	1.863 (2)	P1–C13	1.860 (1)
P2–O5	P2–O3	1.466 (1)	1.463 (1)	P2–O5	1.476 (1)
P2–O3	P2–O4A	1.524 (2)	1.572 (1)	P2–O3	1.568 (1)
P2–O4	P2–O5A	1.642 (3)	1.574 (1)	P2–O4	1.570 (1)
P2–C13	P2–C15	1.834 (2)	1.831 (2)	P2–C13	1.841 (1)
O3–C15	O4A–C17A	1.425 (5)	1.440 (2)	O3–C15	1.457 (1)
O4–C16	O5A–C18A	1.415 (7)	1.438 (2)	O4–C17	1.464 (1)
C7–C8	C1–C2	1.393 (2)	1.398 (2)	C1–C2	1.394 (1)
C7–C12	C1–C6	1.396 (2)	1.400 (2)	C1–C6	1.398 (1)
O2–C13	O2–C15	1.424 (2)	1.424 (2)	O2–C13	1.427 (1)

**Table 4:** The cytostatic effect of the P-compounds studied on human tumor cell cultures.

Compound	Cytostasis [%] at c = 50 µM			
	Cell Line			
	MDA-MB 231	PC-3	Ebc-1	A431
**2b**	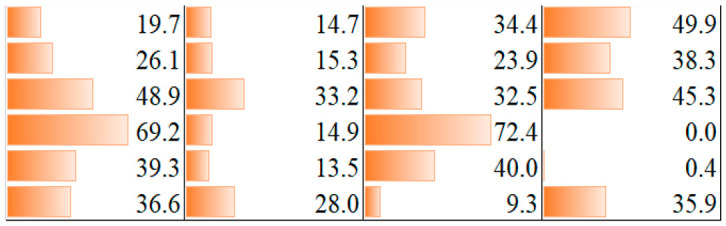			
**3b**				
**5d**				
**5e-1**				
**5f-1**				
**7**				

**Table 5:** 50% inhibitory concentration (IC_50_) values of the P-compounds studied on human tumor cell cultures.

Compound	IC_50_ (µM) ^a,c^			
	Cell Line			
	MDA_MB-231	PC-3	Ebc-1	A431
**2a**	>250	>250	>250	>250
**2b**	>250	>250	>250	>250
**2c**	>250	>250	>250	>250
**2d**	>250	>250	>250	>250
**2e**	>250	>250	>250	>250
**2f**	>250	>250	>250	>250
**3a**	>250	>250	>250	>250
**3b**	>250	>250	>250	>250
**3c**	>250	>250	>250	>250
**5d**	76.7	>250	99.5	40.4
**5e-1**	37.8	149.5	25.9	>250
**5f-1**	100.7	115.8	94.1	110.7
**6 ^b^**	n.d.			
**7**	115.0	>250	>250	>250

^a^ For comparison purposes for the above cell lines, reference compound Daunomycin had an IC_50_ value of 0.20, 4.0, 1.2, and 0.7 µM, respectively [32,33]. ^b^ Compound **6** precipitated in aqueous media. ^c^ The data shaded in yellow refer to the most efficient compounds.

**Table 6:** Details for X-ray data collection and structure refinement for compounds **2d**, **2e.0.5 C_3_H_6_O** and **6**.

	2d	2e.0.5C_3_H_6_O	6
Empirical formula	C_16_H_20_O_5_P_2_	C_18_H_24_O_5_P_2_.0.5C_3_H_6_O	C_18_H_24_O_5_P_2_
Formula mass	354.26	411.35	382.31
T [K]	123 (2)	123 (2)	123 (2)
Crystal size [mm]	0.20 × 0.02 × 0.02	0.35 × 0.20 × 0.10	0.25 × 0.20 × 0.15
Crystal description	colorless rod	colorless block	colorless block
Crystal system	monoclinic	monoclinic	triclinic
Space group	*P*21/*c*	*C*2/*c*	*P*21/*n*
a [Å]	9.1252 (3)	13.8562 (3)	8.6609 (2)
b [Å]	18.1309 (6)	10.4172 (2)	9.8169 (2)
c [Å]	10.1680 (4)	28.5479 (7)	22.1229 (5)
α [°]	90.0	90.0	90.0
β [°]	94.892 (3)	96.649 (2)	96.193 (2)
γ [°]	90.0	90.0	90.0
V [Å^3^]	1676.15 (10)	4092.97 (16)	1869.98 (7)
Z	4	8	4
ρ_calcd_. [g cm^−3^]	1.404	1.335	1.358
μ [mm^−1^]	0.281	0.242	0.258
*F* (000)	744	1744	808
Θ range [°]	2.24–25.24	2.45–25.24	2.27–25.24
Index ranges	−12 ≤ *h* ≤ 12	−17 ≤ *h* ≤ 17	−12 ≤ *h* ≤ 12

## Data Availability

The data presented in this study are available on request from the corresponding authors.

## Supplementary Materials

The following supporting information can be downloaded at: https://www.mdpi.com/article/10.3390/molecules28166037/s1, X-ray data for compounds **2d**, **2e** and **6**; ^31^P, ^13^C and ^1^H NMR spectra of the compounds prepared.
