# Supplementary material for: A Study of the Bisphosphonic Derivatives from the Pudovik Reaction of Dialkyl α-Oxophosphonates and >P(O)H Reagents: X-ray Structure and Bioactivity
Source: Molecules. 2023 Aug 12;28(16):6037. doi: 10.3390/molecules28166037 (PMC10459769; doi:10.3390/molecules28166037)
Supplement: Supplementary file 1 [file molecules-28-06037-s001.zip › molecules-2540891-supplementary.pdf]

Supplementary Materials

A Study on Bisphosphonic Derivatives Coming from the Pudovik Reaction of Dialkyl α-Oxophosphonates and >P(O)H Reagents: X-Ray Structure and Bioactivity

Zsuzsanna Szalai,[1] Boldizsár Tóth,[1] Rita Oláhné Szabó,[2,3] Szilvia Bősze,[2] László Drahos,[4] Konstantin Karaghiosoff,[5] Mátyás Czugler[1] and György Keglevich[1]

[1]*Department of Organic Chemistry and Technology, Faculty of Chemical Technology and Biotechnology, Budapest University of Technology and Economics, 1521 Budapest, Hungary*

[2]*Eötvös Loránd Research Network (ELKH), Research Group of Peptide Chemistry, Eötvös Loránd University, 1117 Budapest, Hungary*

[3]*Department of Genetics, Cell-and Immunobiology, Semmelweis University, Nagyvárad tér 4, 1089 Budapest,*

[4]*MS Proteomics Research Group, Research Centre for Natural Sciences, 1117 Budapest, Hungary*

[5]*Department Chemie, Ludwig-Maximilians-Universitat München, Butenandtstr. 5-13, D-81377 München, Germany*

Contents

1. Geometrical data for compounds 2d, 2e and 6 obtained from the X-ray measurements.... 2
2. Spectra of the compounds 2a-f, 3a, 3b-1 + 3b-2, 3c-1 + 3c-2, 5d-1 + 5d-2, 5e-1 and 5f-1 synthesized 5

1. Geometrical data for compounds 2d, 2e and 6 obtained from the X-ray measurements

SI Table S1. Selected bond lengths (Å) of compound **2d**, **2e** and **6**.

**2d**		**2e**		**6**	
**P1 – O1**	1.493(1)	**P1 – O1**	1.485(1)	**P1 – O1**	1.488(1)
**P1 – C7**	1.801(2)	**P1 – C1**	1.809(2)	**P1 – C7**	1.807(1)
**P1 – C1**	1.811(2)	**P1 – C8**	1.809(2)	**P1 – C1**	1.809(1)
**P1 – C13**	1.863(2)	**P1 – C15**	1.862(2)	**P1 – C13**	1.860(1)
**P2 – O5**	1.463(1)	**C1 – C2**	1.393(2)	**P2 – O5**	1.476(1)
**P2 – O3**	1.572(1)	**C1 – C6**	1.396(2)	**P2 – O3**	1.568(1)
**P2 – O4**	1.574(1)	**O4A – C17A**	1.425(5)	**P2 – O4**	1.570(1)
**P2 – C13**	1.831(2)	**O4A – P2**	1.524(2)	**P2 – C13**	1.841(1)
**O4 – C16**	1.438(2)	**P2 – O3**	1.466(1)	**O3 – C15**	1.457(1)
**O2 – C13**	1.424(2)	**P2 – O5A**	1.642(3)	**O4 – C17**	1.464(1)
**C7 – C8**	1.398(2)	**P2 – C15**	1.834(2)	**C1 – C2**	1.394(1)
**C7 – C12**	1.400(2)	**O2 – C15**	1.424(2)	**C1 – C6**	1.398(1)
**C12 – C11**	1.389(2)	**C2 – C3**	1.385(2)	**O2 – C13**	1.427(1)
**C5 – C4**	1.384(3)	**O5A – C18A**	1.415(7)	**C8 – C9**	1.395(2)
**C5 – C6**	1.391(2)	**C4 – C3**	1.394(3)	**C2 – C3**	1.390(2)
**C8 – C9**	1.390(2)	**C4 – C7**	1.512(2)	**C17 – C18**	1.490(2)
**C11 – C10**	1.386(3)	**C5 – C6**	1.391(2)	**C9 – C10**	1.385(2)
**C10 – C9**	1.384(3)	**O6 – C20**	1.176(4)	**C15 – C16**	1.495(2)
**O3 – C15**	1.440(2)	**C15 – C16**	1.528(2)	**C10 – C11**	1.389(2)
**C1 – C6**	1.390(2)	**C8 – C13**	1.389(2)	**C13 – C14**	1.536(1)
**C1 – C2**	1.403(2)	**C8 – C9**	1.397(2)	**C7 – C12**	1.396(2)
**C14 – C13**	1.535(2)	**C19 – C20**	1.482(3)	**C7 – C8**	1.397(2)
**C3 – C4**	1.385(3)	**C9 – C10**	1.390(2)	**C6 – C5**	1.392(2)
**C3 – C2**	1.385(2)	**C10 – C11**	1.391(2)	**C5 – C4**	1.392(2)
		**C11 – C12**	1.391(2)	**C4 – C3**	1.384(2)
		**C11 – C14**	1.511(2)	**C12 – C11**	1.387(2)
		**C12 – C13**	1.388(2)		
		**C4 – C5**	1.389(3)		

SI Table S2. Selected bond angles (°) of compound **2d**, **2e** and **6**.

**2d**		**2e** (i = 1-x, y, 0.5-z; acetone molecule)		**6**	
**O1 – P1 – C7**	112.0(1)	**O1 – P1 – C1**	112.2(1)	**O1 – P1 – C7**	112.1(1)
**O1 – P1 – C1**	111.9(1)	**O1 – P1 – C8**	111.3(1)	**O1 – P1 – C1**	112.3(1)
**C7 – P1 – C1**	107.1(1)	**C1 – P1 – C8**	107.4(1)	**C7 – P1 – C1**	105.3(1)
**O1 – P1 – C13**	111.4(1)	**O1 – P1 – C15**	111.7(1)	**O1 – P1 – C13**	111.2(1)
**C7 – P1 – C13**	109.5(1)	**C1 – P1 – C15**	108.2(1)	**C7 – P1 – C13**	104.6(1)
**C1 – P1 – C13**	104.6(1)	**C8 – P1 – C15**	105.8(1)	**C1 – P1 – C13**	111.0(1)
**O5 – P2 – O3**	116.5(1)	**C2 – C1 – C6**	118.7(2)	**O5 – P2 – O3**	115.9(1)
**O5 – P2 – O4**	114.1(1)	**C2 – C1 – P1**	124.3(1)	**O5 – P2 – O4**	109.2(1)
**O3 – P2 – O4**	102.3(1)	**C6 – C1 – P1**	117.0(1)	**O3 – P2 – O4**	107.7(1)
**O5 – P2 – C13**	115.4(1)	**C17A – O4A – P2**	121.8(4)	**O5 – P2 – C13**	111.1(1)
**O3 – P2 – C13**	101.7(1)	**O3 – P2 – O4A**	118.3(1)	**O3 – P2 – C13**	103.1(1)
**O4 – P2 – C13**	105.1(1)	**O3 – P2 – O5A**	111.3(1)	**O4 – P2 – C13**	109.6(1)
**C16 – O4 – P2**	122.4(1)	**O4A – P2 – O5A**	101.4(2)	**C15 – O3 – P2**	123.0(1)
**C8 – C7 – C12**	119.6(2)	**O3 – P2 – C15**	113.2(1)	**C17 – O4 – P2**	125.0(1)
**C8 – C7 – P1**	116.4(1)	**O4A – P2 – C15**	109.7(1)	**C2 – C1 – C6**	119.3(1)
**C12 – C7 – P1**	123.9(1)	**O5A – P2 – C15**	100.8(1)	**C2 – C1 – P1**	115.3(1)
**C15 – O3 – P2**	118.6(1)	**C3 – C2 – C1**	120.5(2)	**C6 – C1 – P1**	125.4(1)
**C6 – C1 – C2**	119.1(2)	**C18A – O5A – P2**	119.5(4)	**O2 – C13 – C14**	112.4(1)
**C6 – C1 – P1**	123.7(1)	**C5 – C4 – C3**	118.2(2)	**O2 – C13 – P2**	106.7(1)
**O2 – C13 – C14**	111.7(1)	**C5 – C4 – C7**	121.3(2)	**C4 – C3 – C2**	120.1(1)
**O2 – C13 – P2**	103.7(1)	**C3 – C4 – C7**	120.5(2)	**O4 – C17 – C18**	108.0(1)
**C14 – C13 – P2**	110.3(1)	**C2 – C3 – C4**	121.2(2)	**C10 – C9 – C8**	120.1(1)
**O2 – C13 – P1**	109.7(1)	**C4 – C5 – C6**	121.1(2)	**O3 – C15 – C16**	109.3(1)
**C14 – C13 – P1**	108.8(1)	**C5 – C6 – C1**	120.3(2)	**C9 – C10 – C11**	120.2(1)
**P2 – C13 – P1**	112.6(1)	**O2 – C15 – C16**	112.1(1)	**C12 – C11 – C10**	120.0(1)
**C9 – C8 – C7**	119.9(2)	**O2 – C15 – P2**	108.2(1)	**C14 – C13 – P1**	107.2(1)
**C10 – C11 – C12**	120.0(2)	**C16 – C15 – P2**	108.4(1)	**P2 – C13 – P1**	115.7(1)
**C9 – C10 – C11**	120.5(2)	**O2 – C15 – P1**	106.5(1)	**C12 – C7 – C8**	119.3(1)
**C1 – C6 – C5**	120.2(2)	**C16 – C15 – P1**	110.2(1)	**C12 – C7 – P1**	123.0(1)
**C5 – C4 – C3**	120.3(2)	**P2 – C15 – P1**	111.4(1)	**C8 – C7 – P1**	117.7(1)
**C10 – C9 – C8**	120.0(2)	**C13 – C8 – C9**	118.7(1)	**C5 – C6 – C1**	120.0(1)
**C10 – C9 – H9**	120.000	**C13 – C8 – P1**	114.8(1)	**C6 – C5 – C4**	120.3(1)
**C4 – C5 – C6**	120.1(2)	**C9 – C8 – P1**	126.5(1)	**C3 – C4 – C5**	119.9(1)
**C3 – C2 – C1**	120.5(2)	**O6 – C20 – C19**	121.8(2)	**C11 – C12 – C7**	120.4(1)
**C2 – C1 – P1**	117.1(1)	**O6 – C20 – C19**^{i}	121.8(2)	**C9 – C8 – C7**	120.0(1)
**C4 – C3 – C2**	119.8(2)	**C19 – C20 – C19**^{i}	116.4(3)	**C3 – C2 – C1**	120.5(1)
**C11 – C12 – C7**	119.9(2)	**C10 – C9 – C8**	120.3(2)	**C14 – C13 – P2**	107.9(1)
		**C9 – C10 – C11**	120.9(2)	**O2 – C13 – P1**	107.1(1)
		**C12 – C11 – C10**	118.7(2)		
		**C12 – C11 – C14**	119.9(2)		
		**C10 – C11 – C14**	121.4(2)		
		**C13 – C12 – C11**	120.5(2)		
		**C12 – C13 – C8**	120.9(2)		

SI Table S3. Selected torsion angles (°) of compound **2d**, **2e** and **6**.

**2d**		**2e**		**6**	
**O5 – P2 – O4 – C16**	-27.4(2)	**O1 – P1 – C1 – C2**	174.2(1)	**O5 – P2 – O3 – C15**	-32.3(1)
**O3 – P2 – O4 – C16**	-154.1(2)	**C8 – P1 – C1 – C2**	51.6(2)	**O4 – P2 – O3 – C15**	90.3(1)
**C13 – P2 – O4 – C16**	100.1(2)	**C15 – P1 – C1 – C2**	-62.2(2)	**C13 – P2 – O3 – C15**	-153.9(1)
**O1 – P1 – C7 – C8**	-11.0(2)	**O1 – P1 – C1 – C6**	-3.9(2)	**O5 – P2 – O4 – C17**	145.3(1)
**C1 – P1 – C7 – C8**	112.1(1)	**C8 – P1 – C1 – C6**	-126.5(1)	**O3 – P2 – O4 – C17**	18.7(1)
**C13 – P1 – C7 – C8**	-135.1(1)	**C15 – P1 – C1 – C6**	119.7(1)	**C13 – P2 – O4 – C17**	-92.8(1)
**O1 – P1 – C7 – C12**	172.4(1)	**C17A – O4A – P2 – O3**	-25.6(5)	**O1 – P1 – C1 – C2**	-19.3(1)
**C1 – P1 – C7 – C12**	-64.6(2)	**C17A – O4A – P2 – O5A**	-147.6(5)	**C7 – P1 – C1 – C2**	103.0(1)
**C13 – P1 – C7 – C12**	48.3(2)	**C17A – O4A – P2 – C15**	106.4(4)	**C13 – P1 – C1 – C2**	-144.4(1)
**O5 – P2 – O3 – C15**	-56.5(2)	**C6 – C1 – C2 – C3**	-1.1(2)	**O1 – P1 – C1 – C6**	163.5(1)
**O4 – P2 – O3 – C15**	68.7(2)	**P1 – C1 – C2 – C3**	-179.2(1)	**C7 – P1 – C1 – C6**	-74.3(1)
**C13 – P2 – O3 – C15**	177.1(2)	**O3 – P2 – O5A – C18A**	-64.2(5)	**C13 – P1 – C1 – C6**	38.4(1)
**O1 – P1 – C1 – C6**	144.9(2)	**O4A – P2 – O5A – C18A**	62.5(5)	**O5 – P2 – C13 – O2**	-52.0(1)
**C7 – P1 – C1 – C6**	21.9(2)	**C15 – P2 – O5A – C18A**	175.4(5)	**O3 – P2 – C13 – O2**	72.8(1)
**C13 – P1 – C1 – C6**	-94.3(2)	**C1 – C2 – C3 – C4**	1.2(3)	**O4 – P2 – C13 – O2**	-172.8(1)
**O1 – P1 – C1 – C2**	-35.0(2)	**C5 – C4 – C3 – C2**	-0.5(3)	**O5 – P2 – C13 – C14**	69.0(1)
**C7 – P1 – C1 – C2**	-158.1(1)	**C7 – C4 – C3 – C2**	179.0(2)	**O3 – P2 – C13 – C14**	-166.3(1)
**C13 – P1 – C1 – C2**	85.8(1)	**C3 – C4 – C5 – C6**	-0.2(3)	**O4 – P2 – C13 – C14**	-51.8(1)
**C8 – C7 – C12 – C11**	-2.5(2)	**C7 – C4 – C5 – C6**	-179.7(2)	**O5 – P2 – C13 – P1**	-171.0(1)
**P1 – C7 – C12 – C11**	174.0(1)	**C4 – C5 – C6 – C1**	0.3(3)	**O3 – P2 – C13 – P1**	-46.3(1)
**C4 – C3 – C2 – C1**	-0.7(3)	**C2 – C1 – C6 – C5**	0.4(2)	**O4 – P2 – C13 – P1**	68.2(1)
**C6 – C1 – C2 – C3**	0.9(3)	**P1 – C1 – C6 – C5**	178.6(1)	**O1 – P1 – C13 – O2**	179.8(1)
**P1 – C1 – C2 – C3**	-179.2(1)	**O3 – P2 – C15 – O2**	44.3(1)	**C7 – P1 – C13 – O2**	58.6(1)
**O5 – P2 – C13 – O2**	56.1(1)	**O4A – P2 – C15 – O2**	-90.4(2)	**C1 – P1 – C13 – O2**	-54.5(1)
**O3 – P2 – C13 – O2**	-176.8(1)	**O5A – P2 – C15 – O2**	163.3(2)	**O1 – P1 – C13 – C14**	59.0(1)
**O4 – P2 – C13 – O2**	-70.5(1)	**O4B – P2 – C15 – O2**	-63.0(3)	**C7 – P1 – C13 – C14**	-62.2(1)
**O5 – P2 – C13 – C14**	175.9(1)	**O5B – P2 – C15 – C16**	68.6(5)	**C1 – P1 – C13 – C14**	-175.3(1)
**O3 – P2 – C13 – C14**	-57.0(1)	**C9 – C8 – C13 – C12**	0.5(2)	**O1 – P1 – C13 – P2**	-61.4(1)
**O4 – P2 – C13 – C14**	49.3(1)	**P1 – C8 – C13 – C12**	-180.0(1)	**C7 – P1 – C13 – P2**	177.4(1)
**O5 – P2 – C13 – P1**	-62.4(1)	**O3 – P2 – C15 – C16**	-77.5(1)	**C1 – P1 – C13 – P2**	64.3(1)
**O3 – P2 – C13 – P1**	64.7(1)	**O4A – P2 – C15 – C16**	147.9(2)	**O1 – P1 – C7 – C12**	164.6(1)
**O4 – P2 – C13 – P1**	171.0(1)	**O5A – P2 – C15 – C16**	41.5(2)	**C1 – P1 – C7 – C12**	42.3(1)
**O1 – P1 – C13 – O2**	-179.5(1)	**O3 – P2 – C15 – P1**	161.0(1)	**C13 – P1 – C7 – C12**	-74.8(1)
**C7 – P1 – C13 – O2**	-55.1(1)	**O4A – P2 – C15 – P1**	26.4(2)	**O1 – P1 – C7 – C8**	-13.7(1)
**C1 – P1 – C13 – O2**	59.4(1)	**O5A – P2 – C15 – P1**	-80.0(2)	**C1 – P1 – C7 – C8**	-136.0(1)
**O1 – P1 – C13 – C14**	58.0(1)	**O1 – P1 – C15 – O2**	163.7(1)	**C13 – P1 – C7 – C8**	106.9(1)
**C7 – P1 – C13 – C14**	-177.6(1)	**C1 – P1 – C15 – O2**	39.8(1)	**C2 – C1 – C6 – C5**	0.5(2)
**C1 – P1 – C13 – C14**	-63.1(1)	**C8 – P1 – C15 – O2**	-75.1(1)	**P1 – C1 – C6 – C5**	177.6(1)
**O1 – P1 – C13 – P2**	-64.6(1)	**O1 – P1 – C15 – C16**	-74.5(1)	**C1 – C6 – C5 – C4**	0.4(2)
**C7 – P1 – C13 – P2**	59.9(1)	**C1 – P1 – C15 – C16**	161.6(1)	**C6 – C5 – C4 – C3**	-1.0(2)
**C1 – P1 – C13 – P2**	174.3(1)	**C8 – P1 – C15 – C16**	46.7(1)	**C8 – C7 – C12 – C11**	-0.8(2)
**C12 – C7 – C8 – C9**	1.6(2)	**O1 – P1 – C15 – P2**	45.9(1)	**P1 – C7 – C12 – C11**	-179.1(1)
**P1 – C7 – C8 – C9**	-175.1(1)	**C1 – P1 – C15 – P2**	-78.0(1)	**C12 – C7 – C8 – C9**	0.7(2)
**C7 – C12 – C11 – C10**	1.3(3)	**C8 – P1 – C15 – P2**	167.1(1)	**P1 – C7 – C8 – C9**	179.1(1)
**C12 – C11 – C10 – C9**	0.8(3)	**O1 – P1 – C8 – C13**	-7.4(2)	**C6 – C1 – C2 – C3**	-0.8(2)
**C2 – C1 – C6 – C5**	-0.6(3)	**C1 – P1 – C8 – C13**	115.7(1)	**P1 – C1 – C2 – C3**	-178.2(1)
**P1 – C1 – C6 – C5**	179.5(1)	**C15 – P1 – C8 – C13**	-128.9(1)	**C5 – C4 – C3 – C2**	0.6(2)
**C4 – C5 – C6 – C1**	0.1(3)	**O1 – P1 – C8 – C9**	172.1(2)	**C1 – C2 – C3 – C4**	0.3(2)
**C6 – C5 – C4 – C3**	0.1(3)	**C1 – P1 – C8 – C9**	-64.7(2)	**P2 – O4 – C17 – C18**	172.8(1)
**C2 – C3 – C4 – C5**	0.2(3)	**C15 – P1 – C8 – C9**	50.7(2)	**C7 – C8 – C9 – C10**	-0.1(2)
**C11 – C10 – C9 – C8**	-1.6(3)	**C13 – C8 – C9 – C10**	-0.4(3)	**P2 – O3 – C15 – C16**	108.6(1)
**C7 – C8 – C9 – C10**	0.4(3)	**P1 – C8 – C9 – C10**	-179.9(1)	**C8 – C9 – C10 – C11**	-0.4(2)
		**C8 – C9 – C10 – C11**	-0.5(3)	**C7 – C12 – C11 – C10**	0.4(2)
		**C9 – C10 – C11 – C12**	1.2(3)	**C9 – C10 – C11 – C12**	0.2(2)
		**C9 – C10 – C11 – C14**	-178.2(2)		
		**C10 – C11 – C12 – C13**	-1.1(3)		
		**C14 – C11 – C12 – C13**	178.3(2)		
		**C11 – C12 – C13 – C8**	0.3(3)		

2. Spectra of the compounds 2a-f, 3a, 3b-1 + 3b-2, 3c-1 + 3c-2, 5d-1 + 5d-2, 5e-1 and 5f-1 synthesized

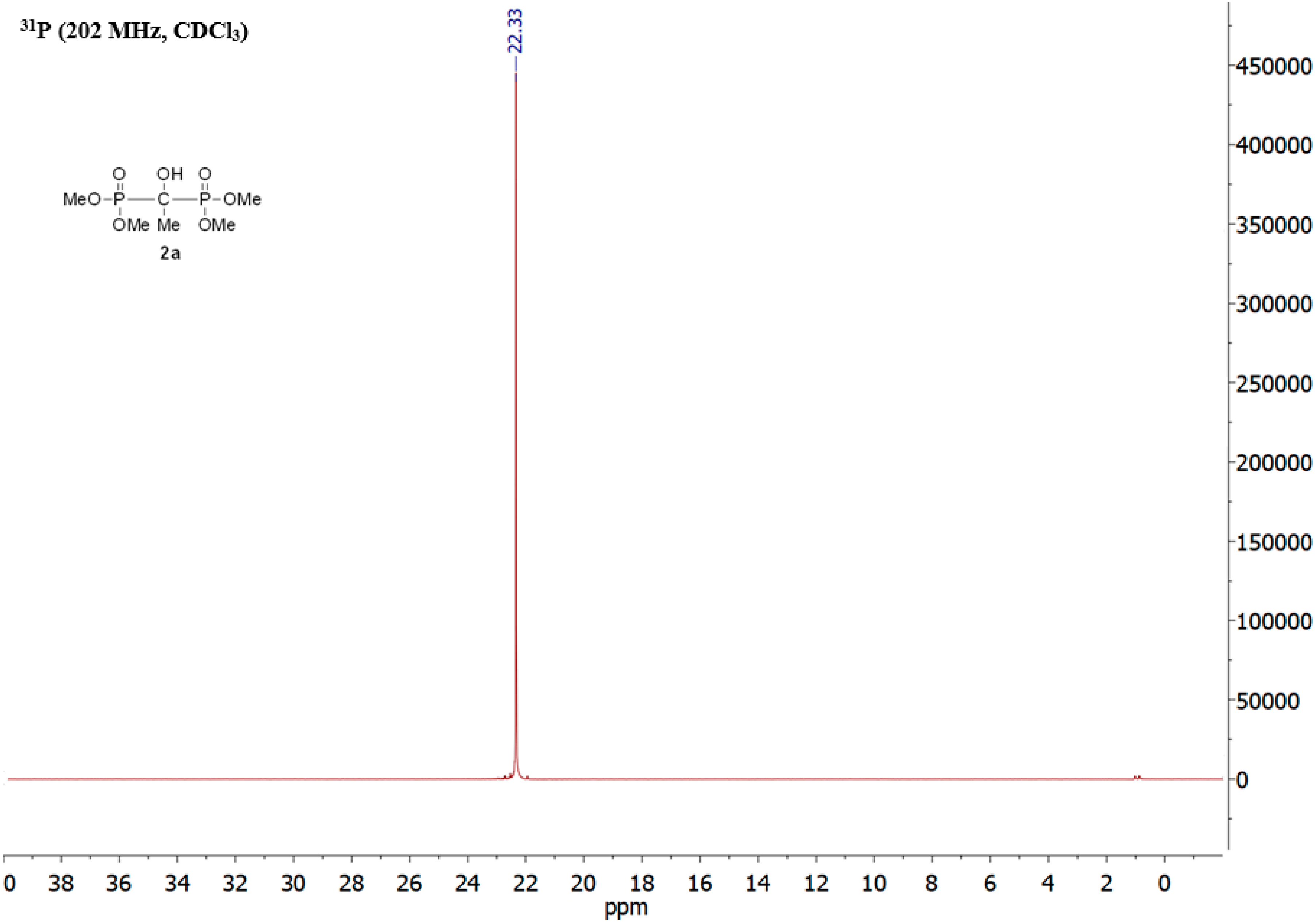

31P (202 MHz, CDCl3)
22.33
MeO
OMe
OH
Me
2a
450000
400000
350000
300000
250000
200000
150000
100000
50000
0
0 38 36 34 32 30 28 26 24 22 20 18 16 14 12 10 8 6 4 2 0
ppm

^{13}C (126 MHz, CDCl$_3$)

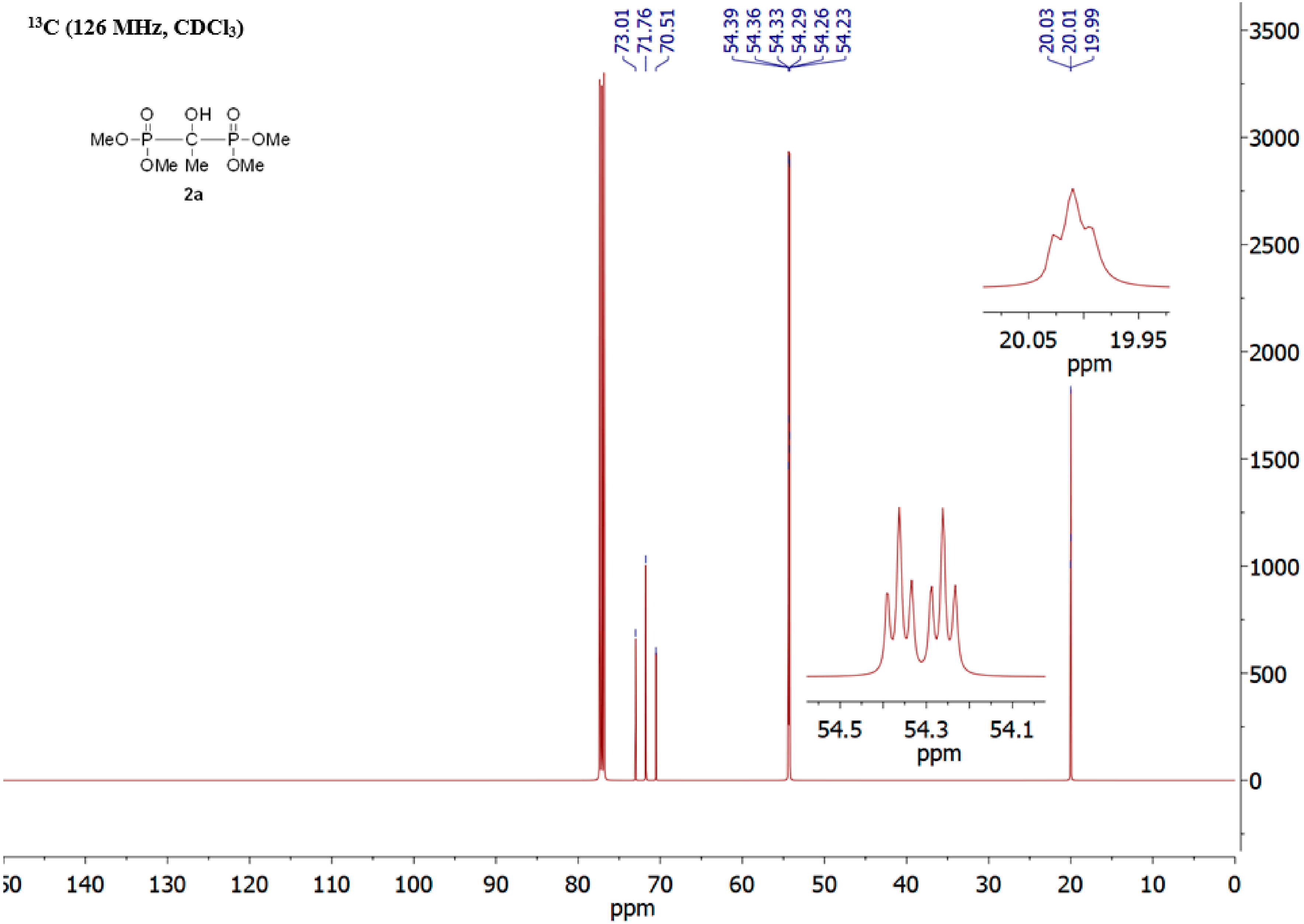

^{1}H (500 MHz, CDCl3)

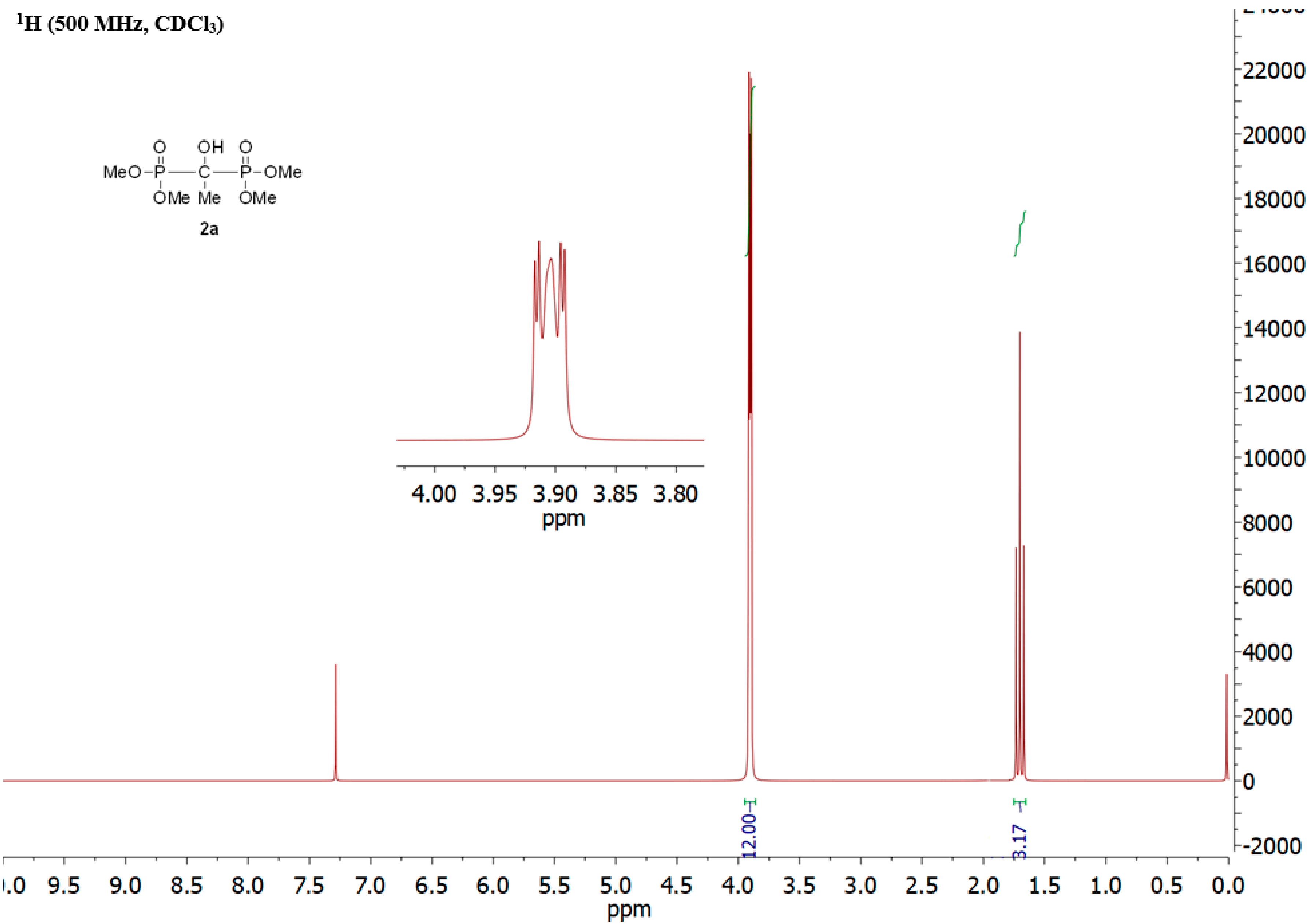

^{31}P (202 MHz, $CDCl_3$)

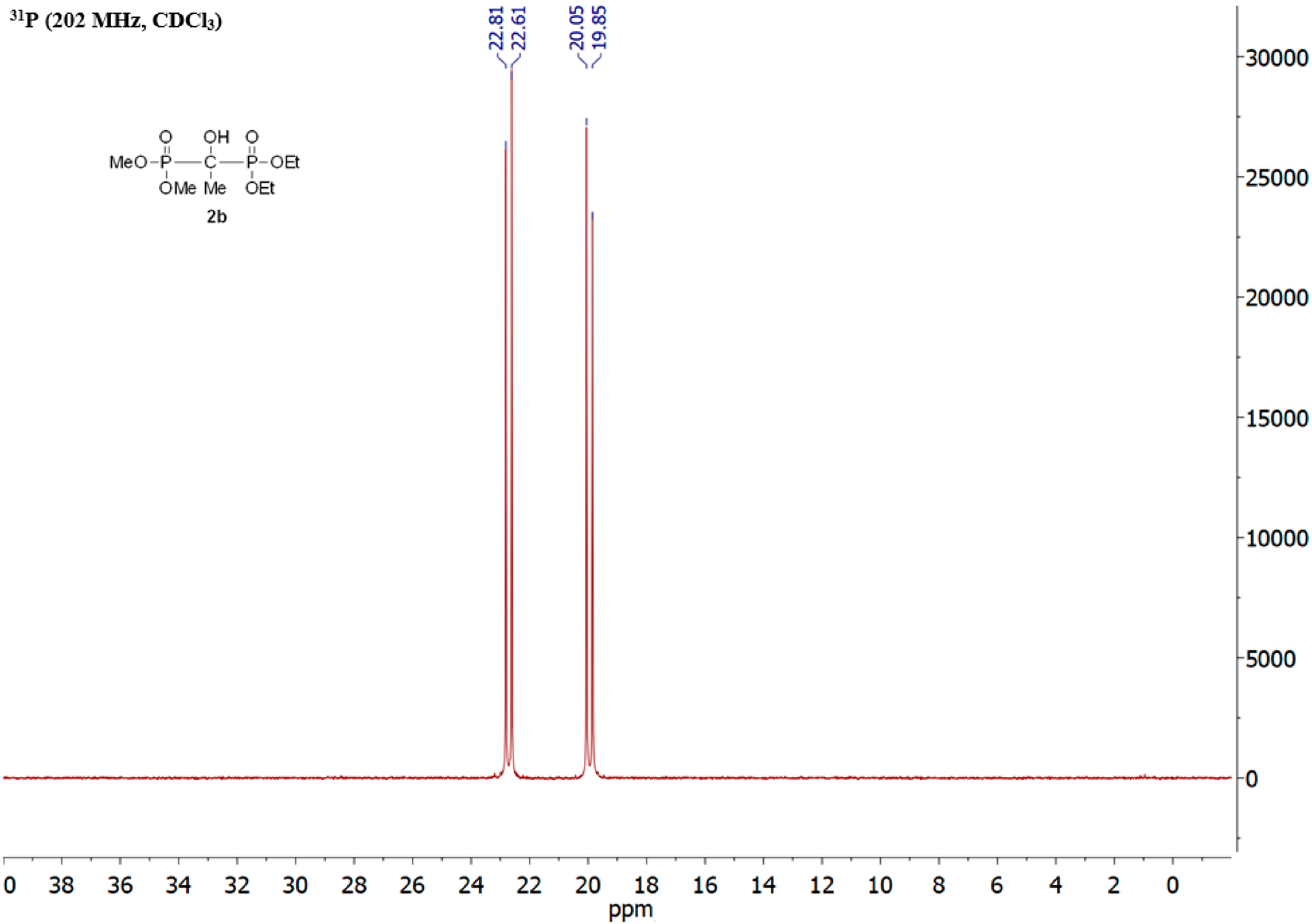

^{13}C (75 MHz, CDCl3)

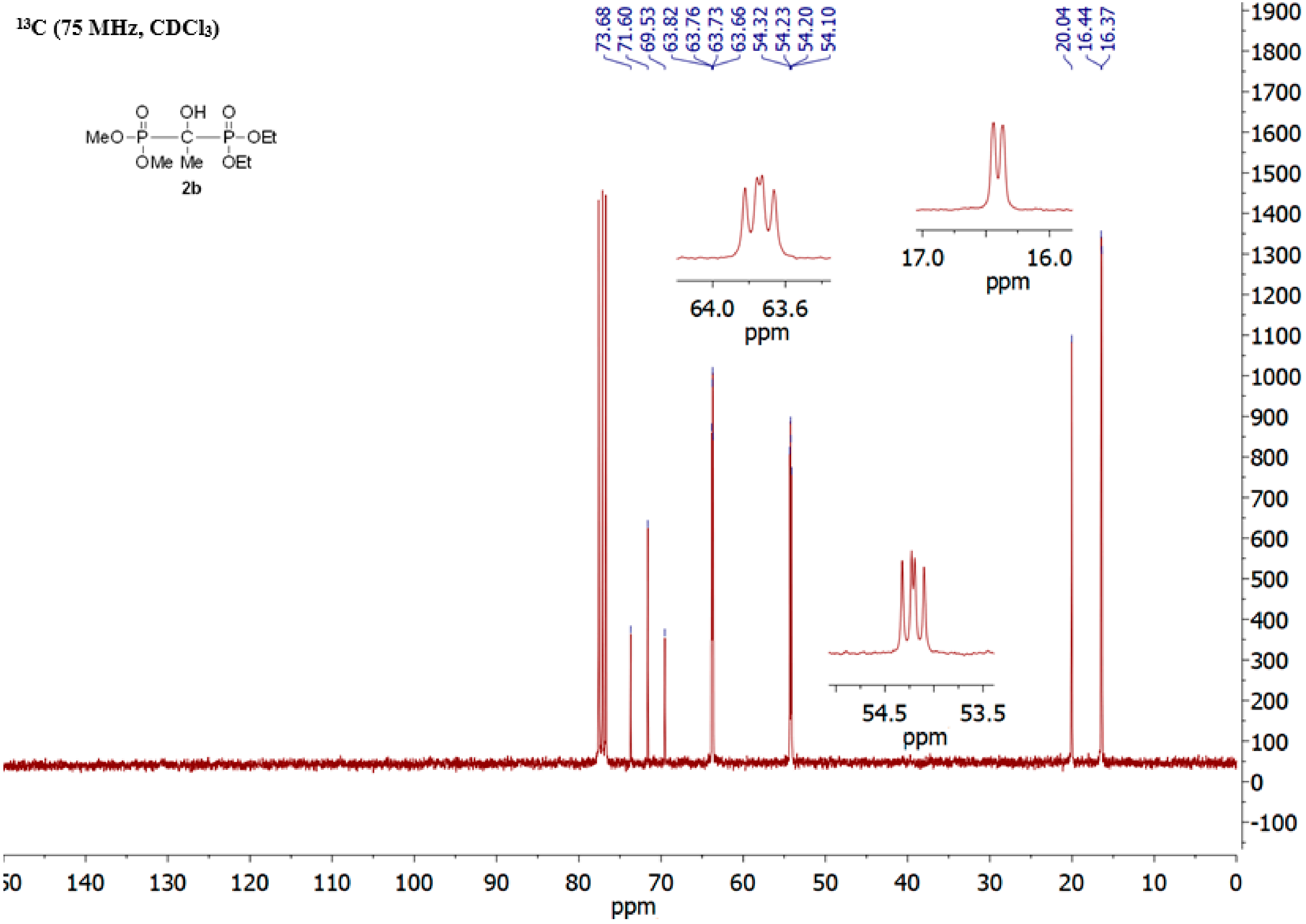

^{1}H (300 MHz, $CDCl_3$)

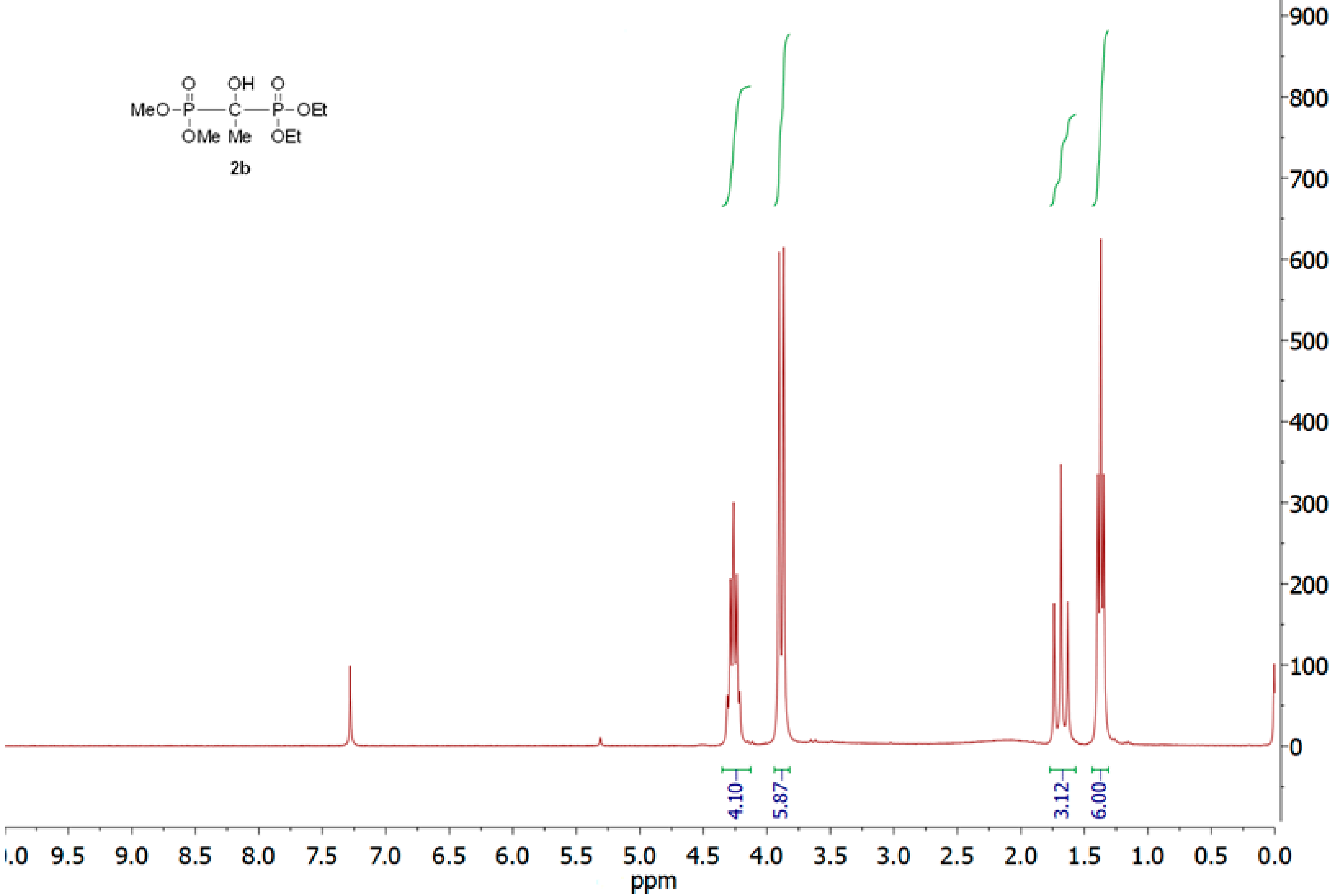

2b

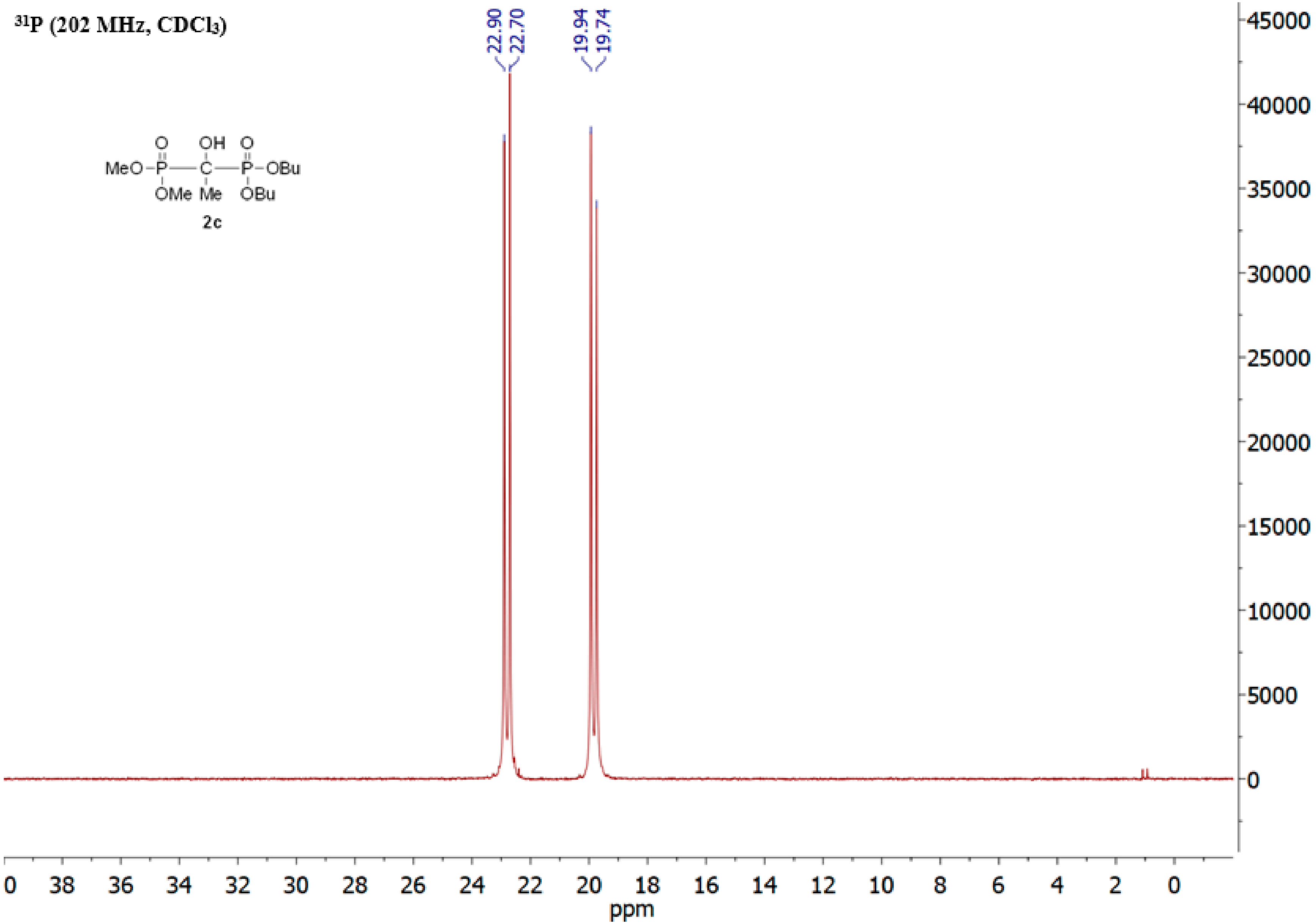

³¹P (202 MHz, CDCl₃)
22.90
22.70
19.94
19.74
MeO
OMe
OH
Me
OBu
OBu
2c
45000
40000
35000
30000
25000
20000
15000
10000
5000
0
0 38 36 34 32 30 28 26 24 22 20 18 16 14 12 10 8 6 4 2 0
ppm

^{13}C (126 MHz, $CDCl_3$)

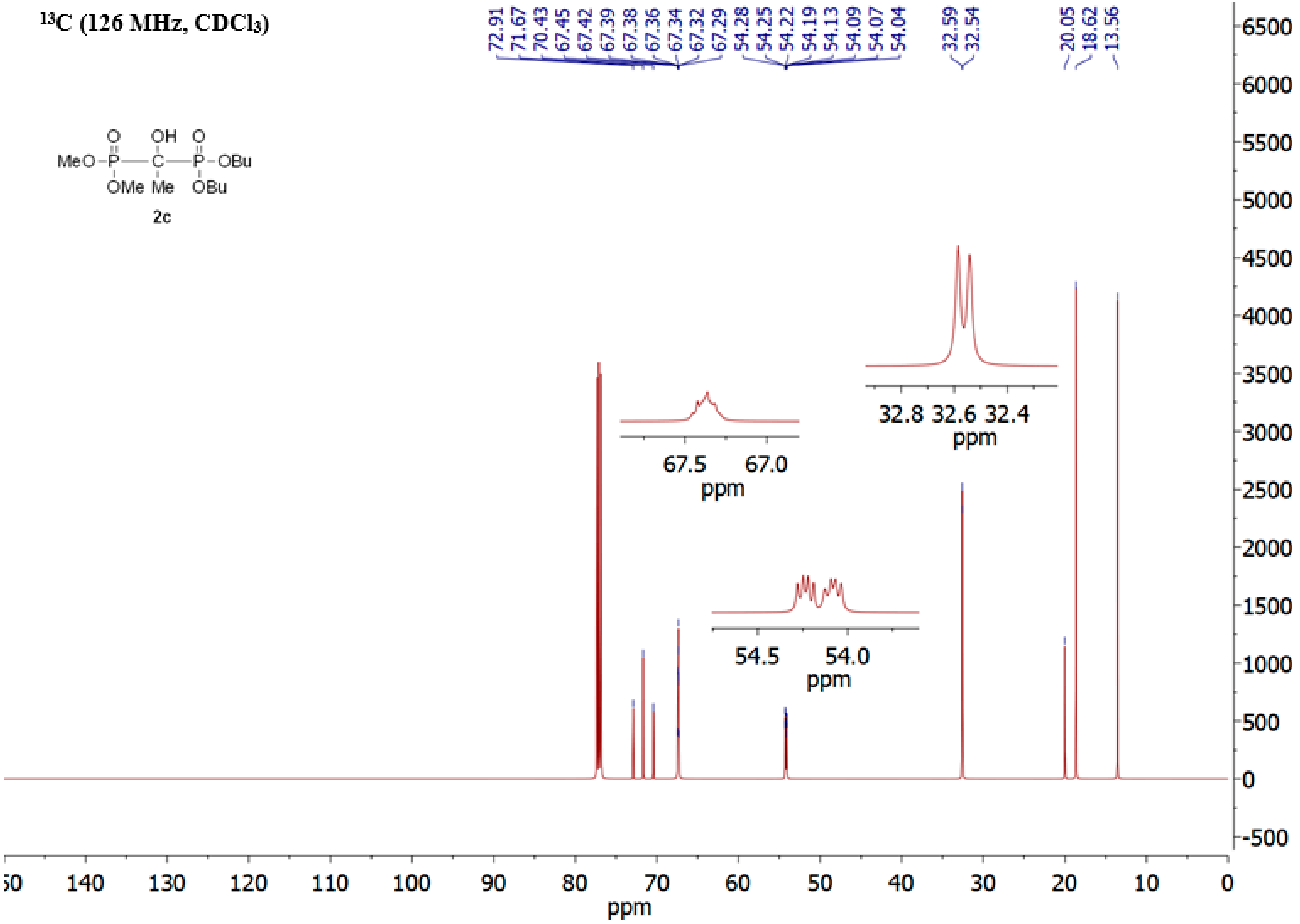

^{1}H (300 MHz, $CDCl_3$)

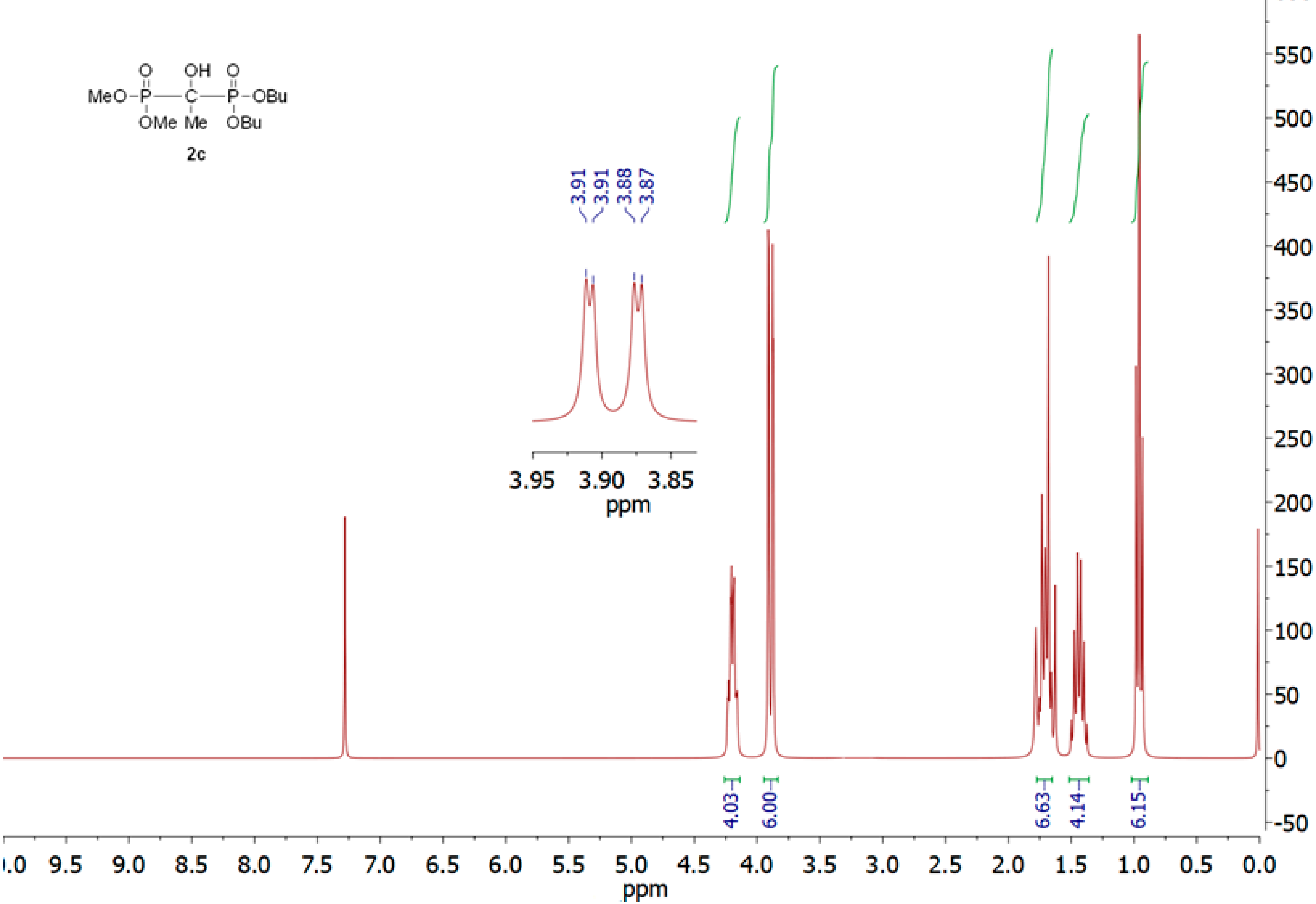

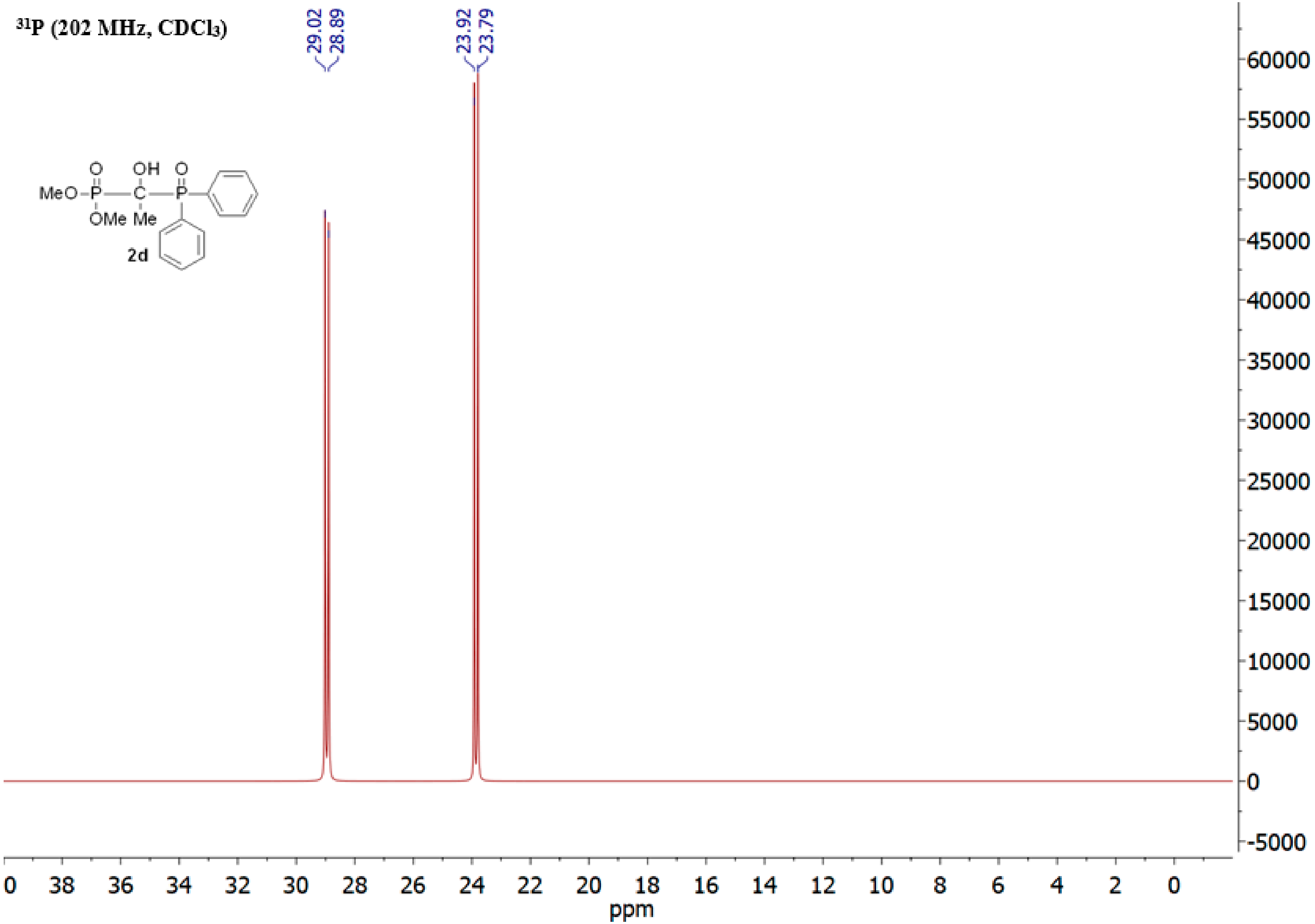

31P (202 MHz, CDCl3)
29.02
28.89
23.92
23.79
MeO
OMe
OH
Me
2d
60000
55000
50000
45000
40000
35000
30000
25000
20000
15000
10000
5000
0
-5000
0
38
36
34
32
30
28
26
24
22
20
18
16
14
12
10
8
6
4
2
0
ppm

^{13}C (126 MHz, $CDCl_3$)

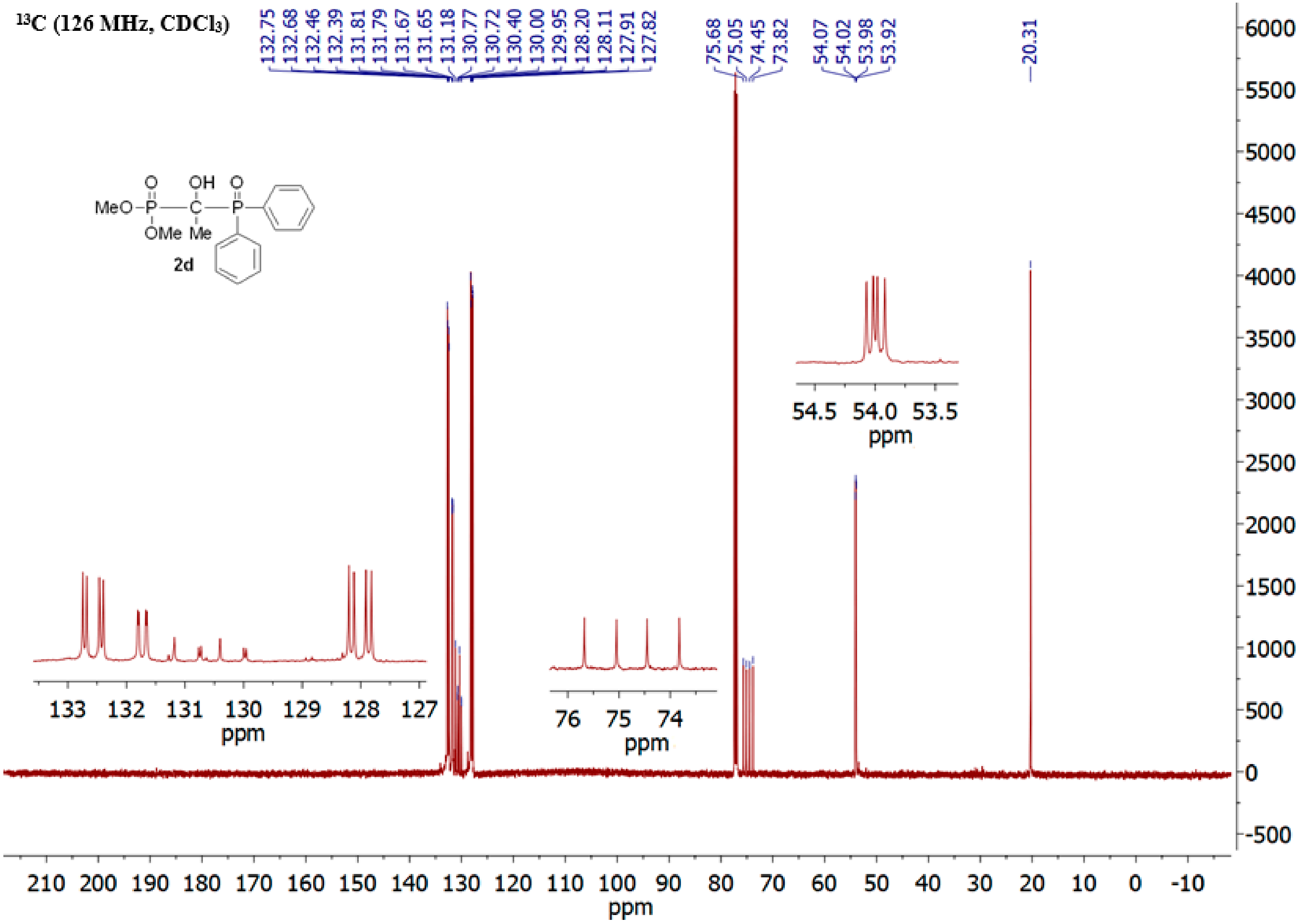

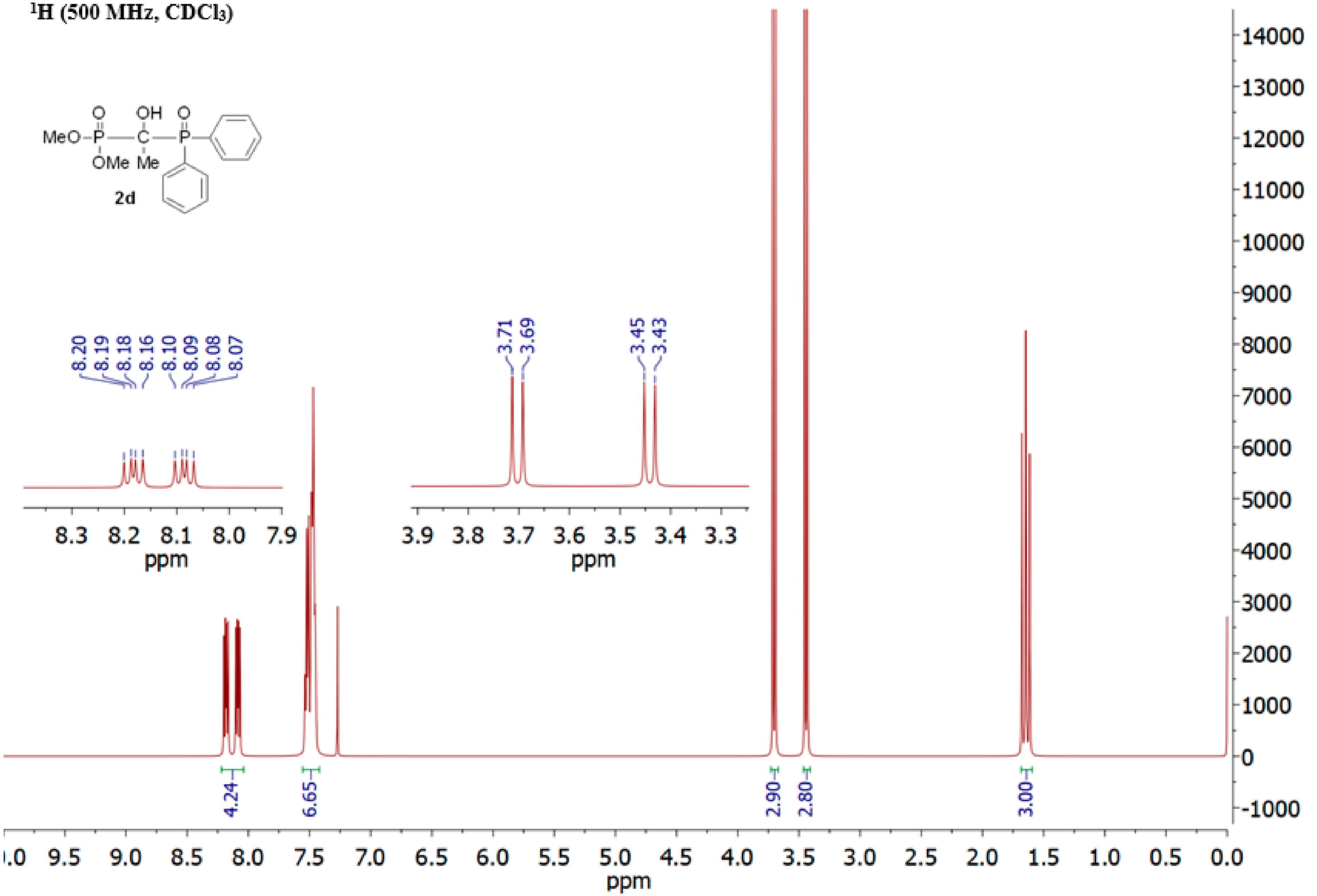

1H (500 MHz, CDCl3)
MeO P OMe C OH Me P O O
2d
8.20 8.19 8.18 8.16 8.10 8.09 8.08 8.07
8.3 8.2 8.1 8.0 7.9
ppm
3.71 3.69 3.45 3.43
3.9 3.8 3.7 3.6 3.5 3.4 3.3
ppm
4.24 6.65 2.90 2.80 3.00
14000 13000 12000 11000 10000 9000 8000 7000 6000 5000 4000 3000 2000 1000 0 -1000
9.5 9.0 8.5 8.0 7.5 7.0 6.5 6.0 5.5 5.0 4.5 4.0 3.5 3.0 2.5 2.0 1.5 1.0 0.5 0.0
ppm

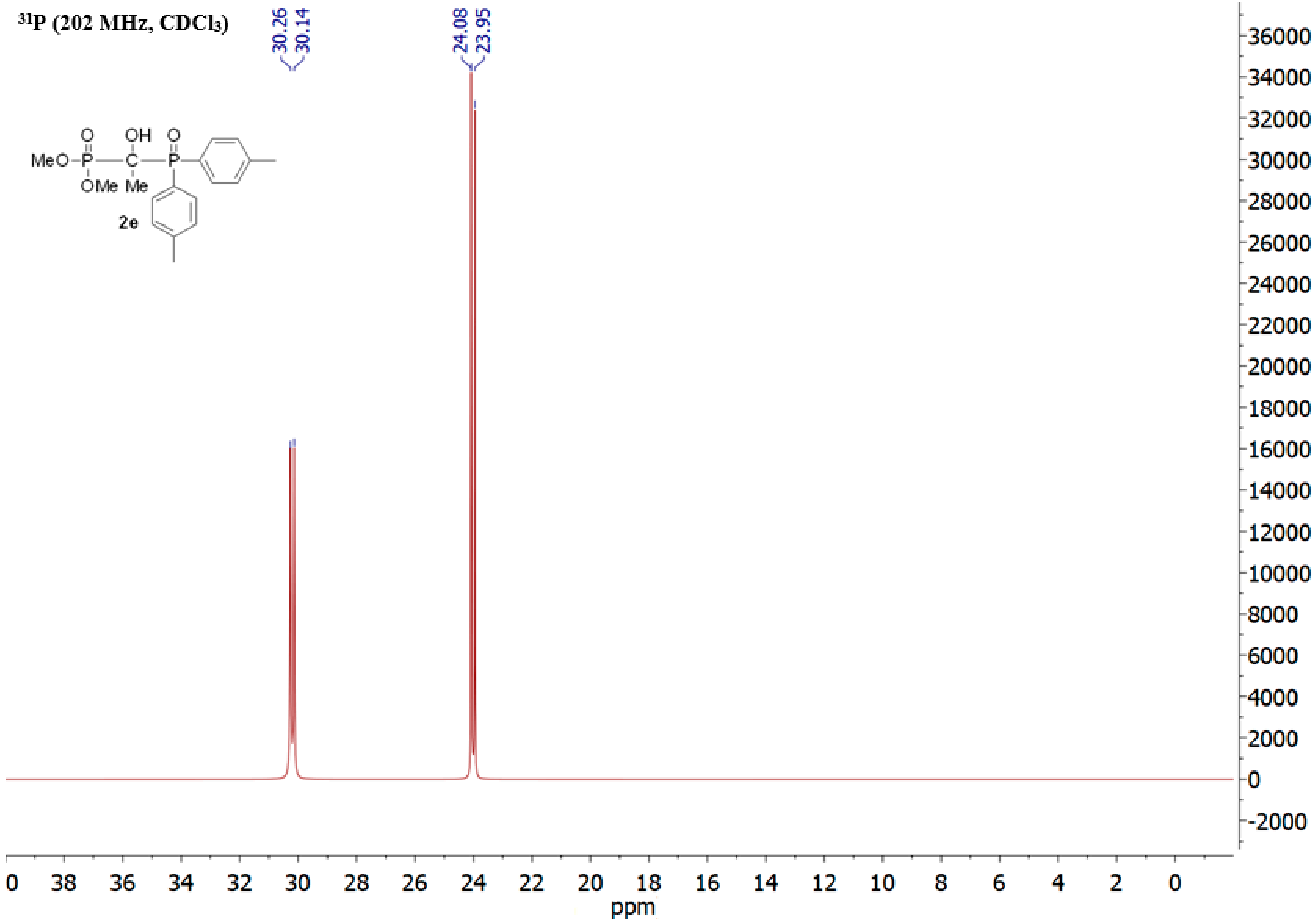

31P (202 MHz, CDCl3)
30.26
30.14
24.08
23.95
MeO
OMe
OH
Me
2e
ppm

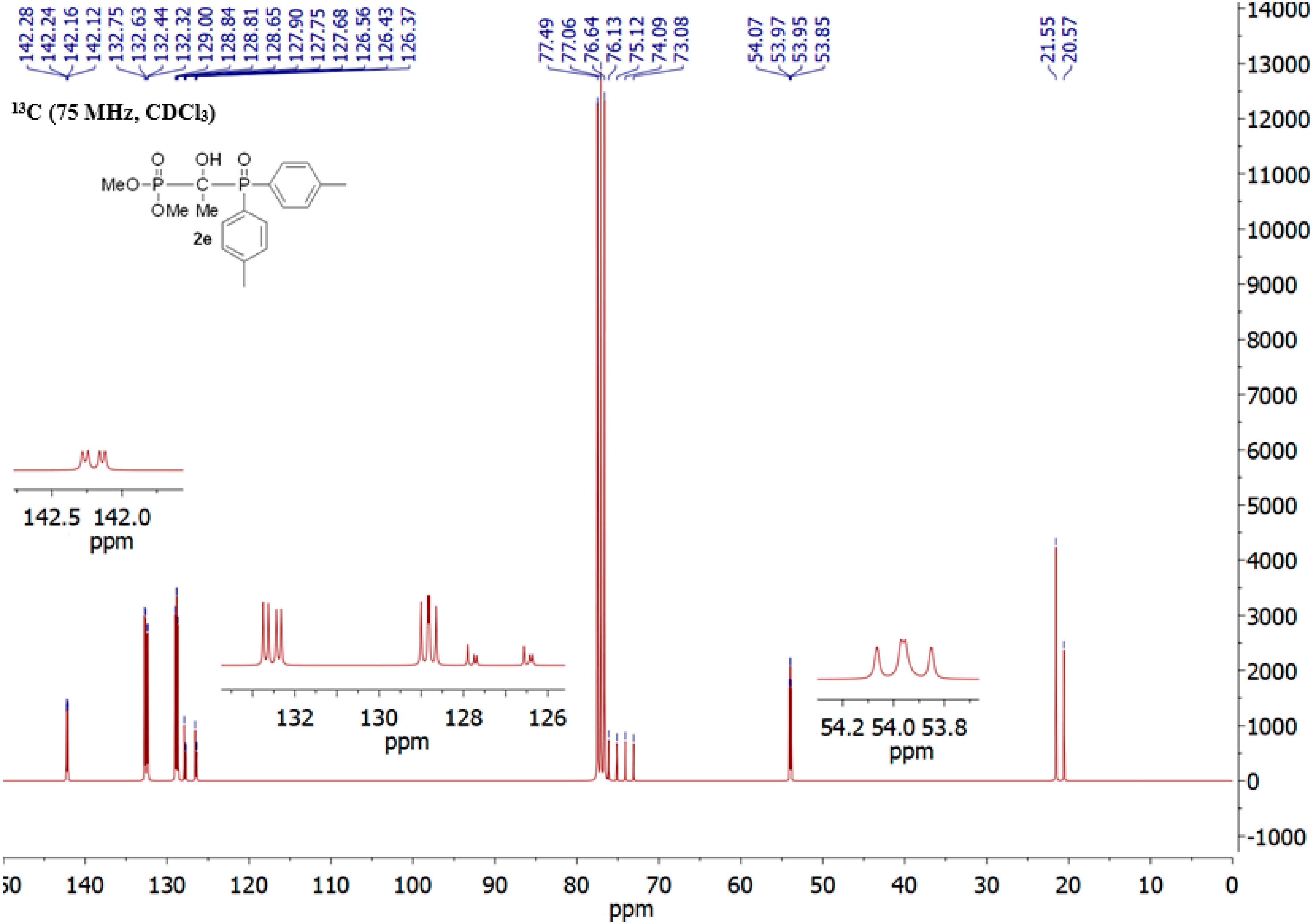

142.28
142.24
142.16
142.12
132.75
132.63
132.44
132.32
129.00
128.84
128.81
128.65
127.90
127.75
127.68
126.56
126.43
126.37
77.49
77.06
76.64
76.13
75.12
74.09
73.08
54.07
53.97
53.95
53.85
21.55
20.57
^{13}C (75 MHz, CDCl$_3$)
MeO
OMe
OH
Me
2e
142.5 142.0
ppm
132 130 128 126
ppm
54.2 54.0 53.8
ppm
ppm

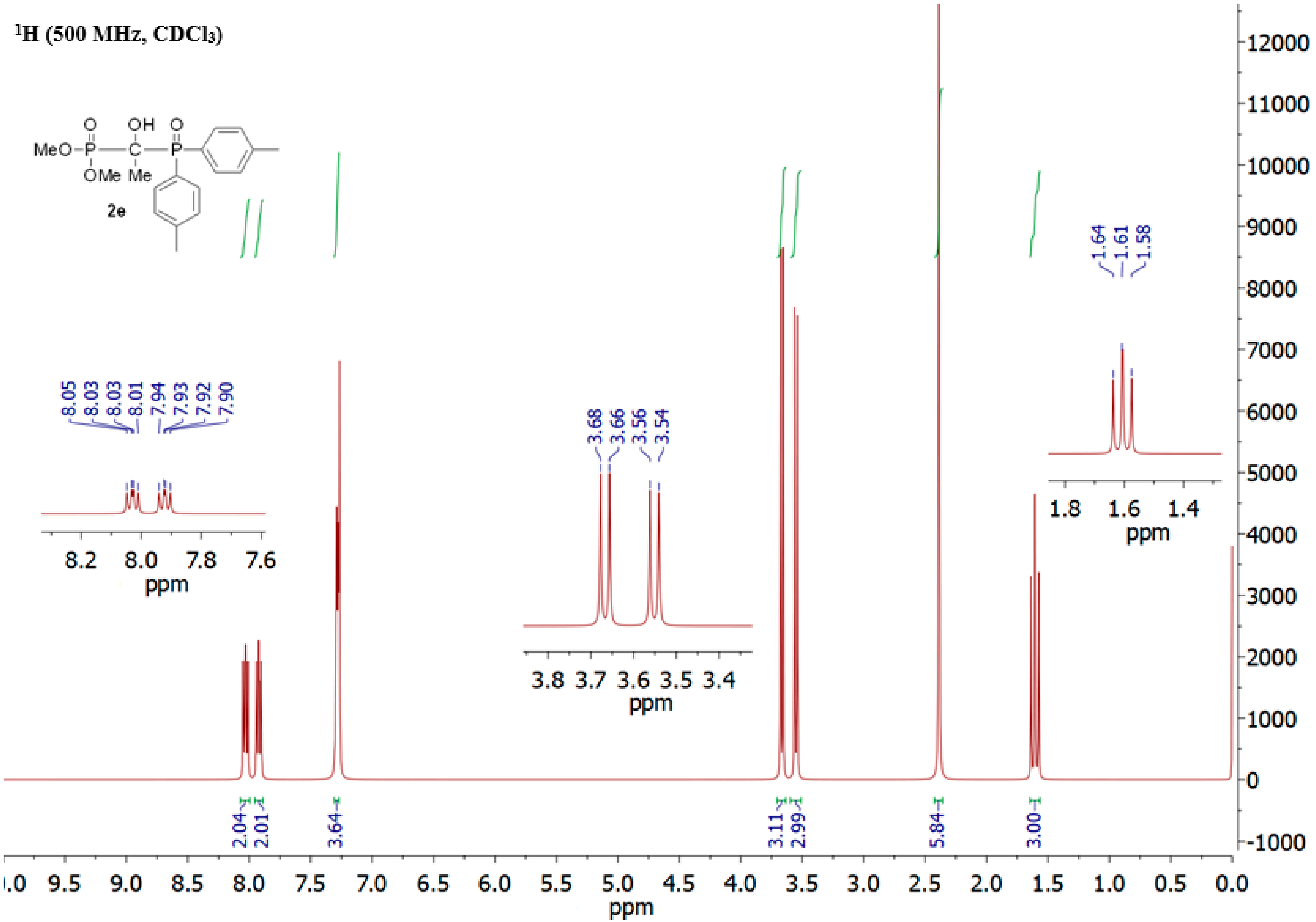

1H (500 MHz, CDCl3)
MeO
OMe
Me
OH
O
P
C
2e
8.05
8.03
8.03
8.01
7.94
7.93
7.92
7.90
3.68
3.66
3.56
3.54
1.64
1.61
1.58
2.04
2.01
3.64
3.11
2.99
5.84
3.00
ppm

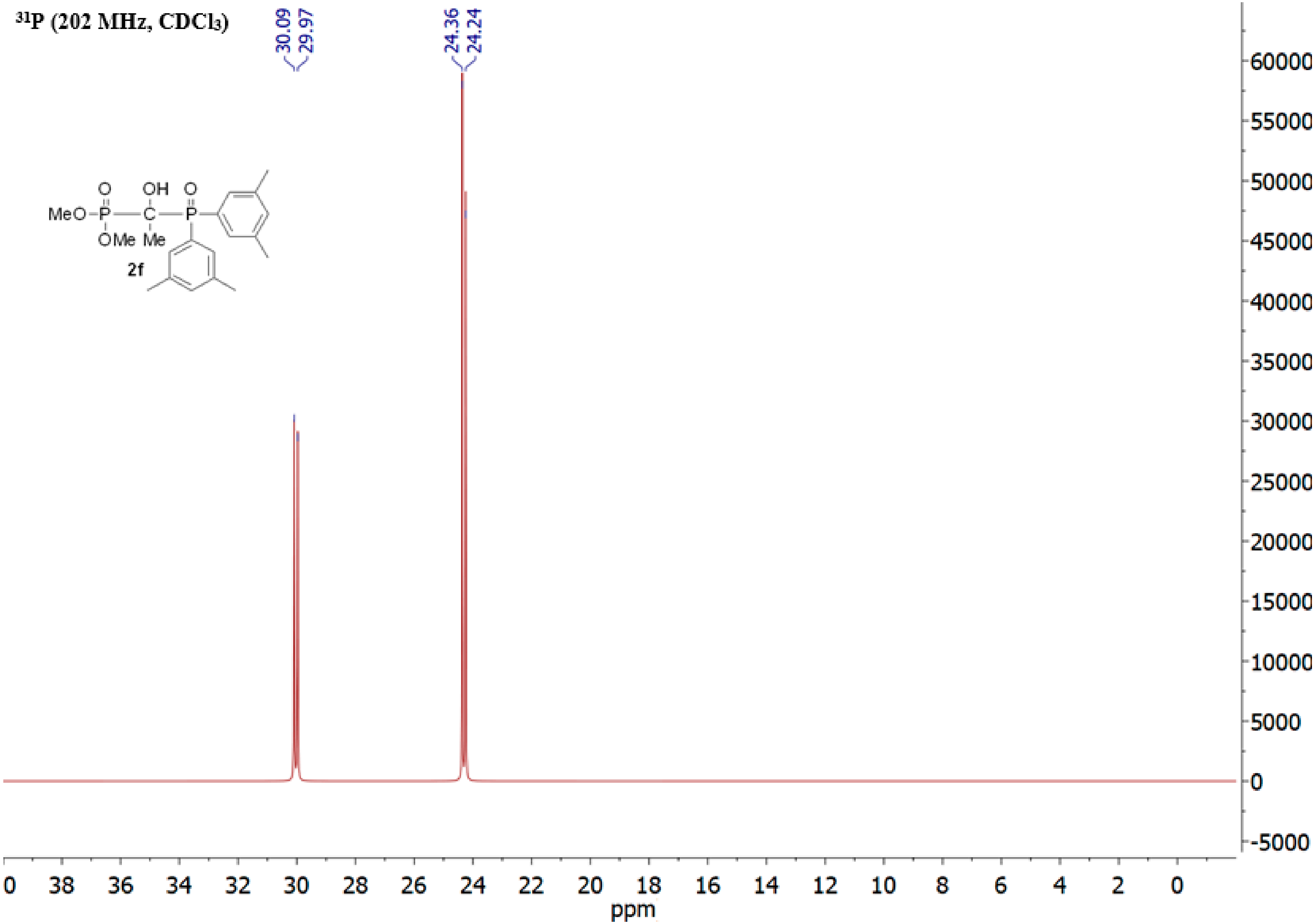

^{31}P (202 MHz, CDCl$_3$)
30.09
29.97
24.36
24.24
MeO
OMe
OH
Me
2f
60000
55000
50000
45000
40000
35000
30000
25000
20000
15000
10000
5000
0
-5000
0 38 36 34 32 30 28 26 24 22 20 18 16 14 12 10 8 6 4 2 0
ppm

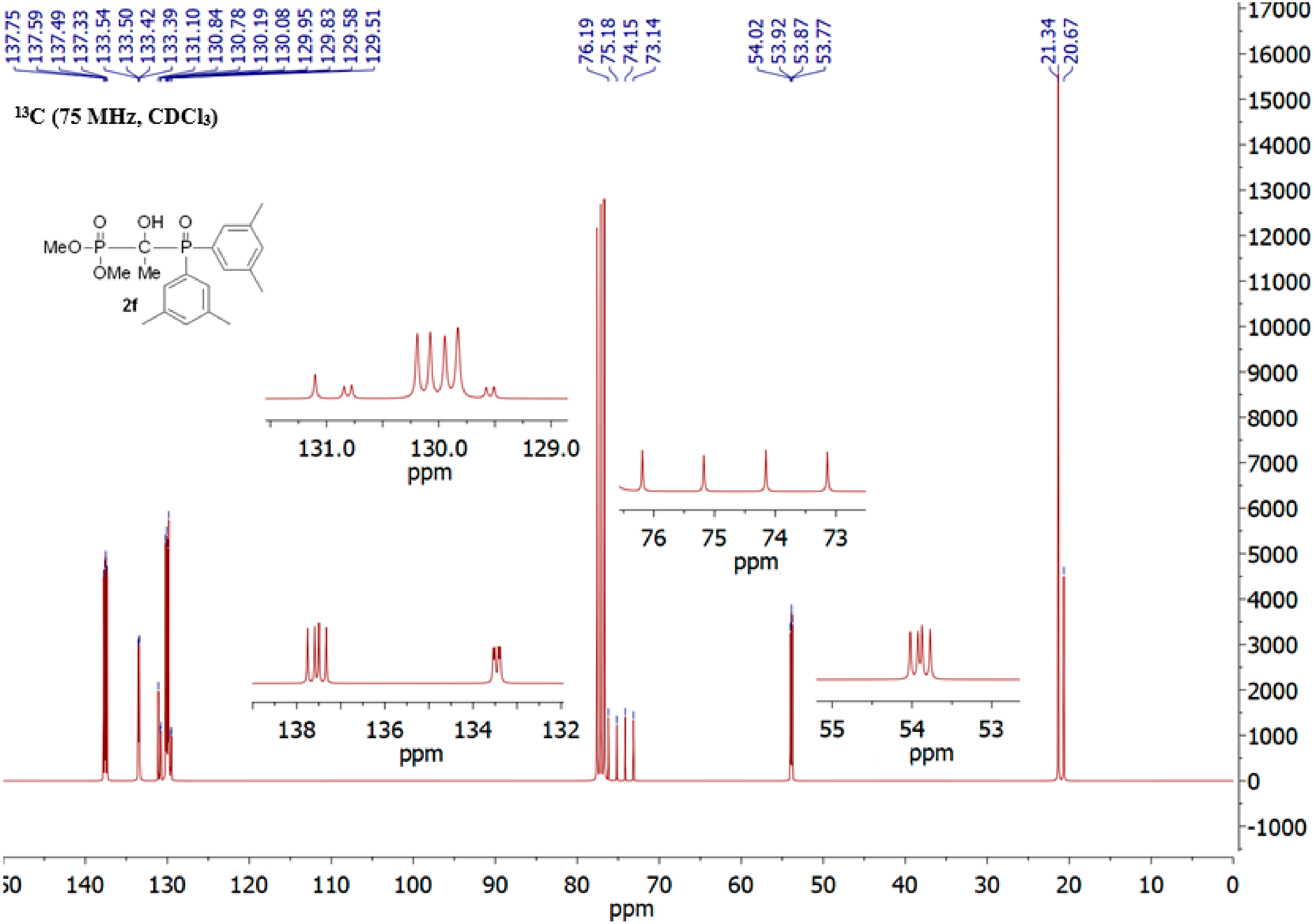
137.75
137.59
137.49
137.33
133.54
133.50
133.42
133.39
131.10
130.84
130.78
130.19
130.08
129.95
129.83
129.58
129.51
76.19
75.18
74.15
73.14
54.02
53.92
53.87
53.77
21.34
20.67
13C (75 MHz, CDCl3)
MeO
OMe
OH
Me
O
P
C
2f
ppm

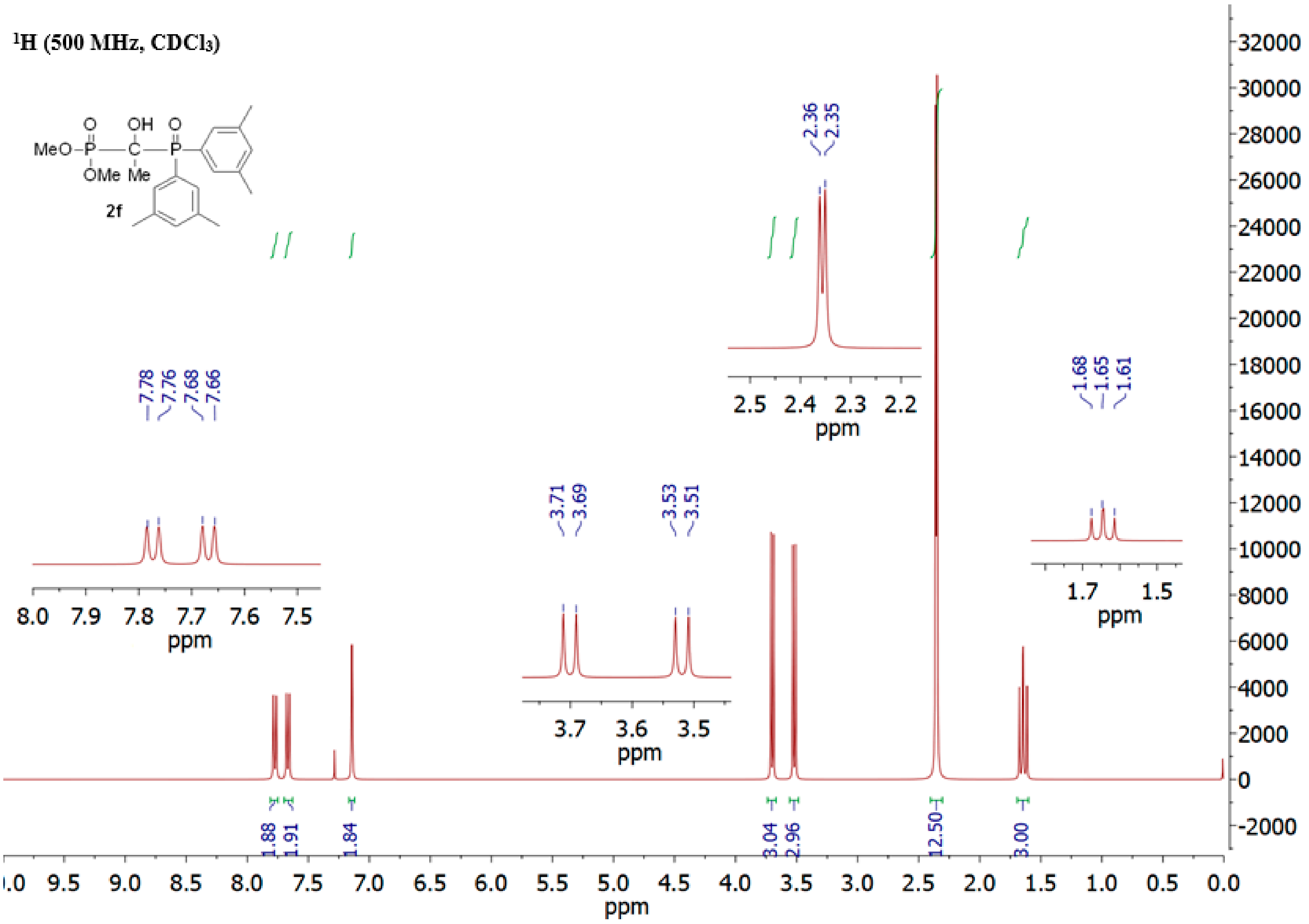

1H (500 MHz, CDCl3)
MeO
OMe
OH
Me
2f
7.78
7.76
7.68
7.66
3.71
3.69
3.53
3.51
2.36
2.35
1.68
1.65
1.61
1.88
1.91
1.84
3.04
2.96
12.50
3.00
ppm

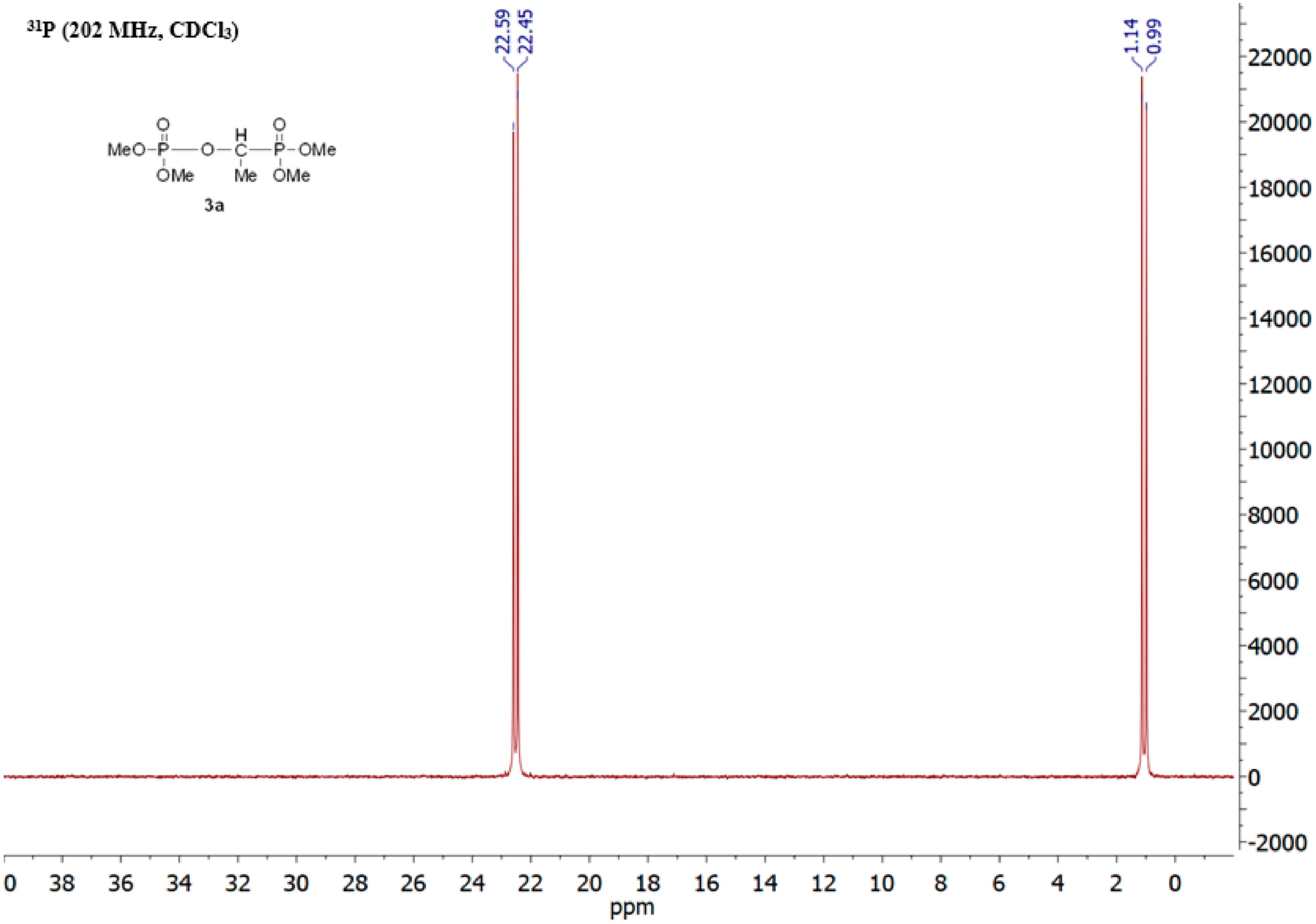

31P (202 MHz, CDCl3)
3a
22.59
22.45
1.14
0.99
ppm

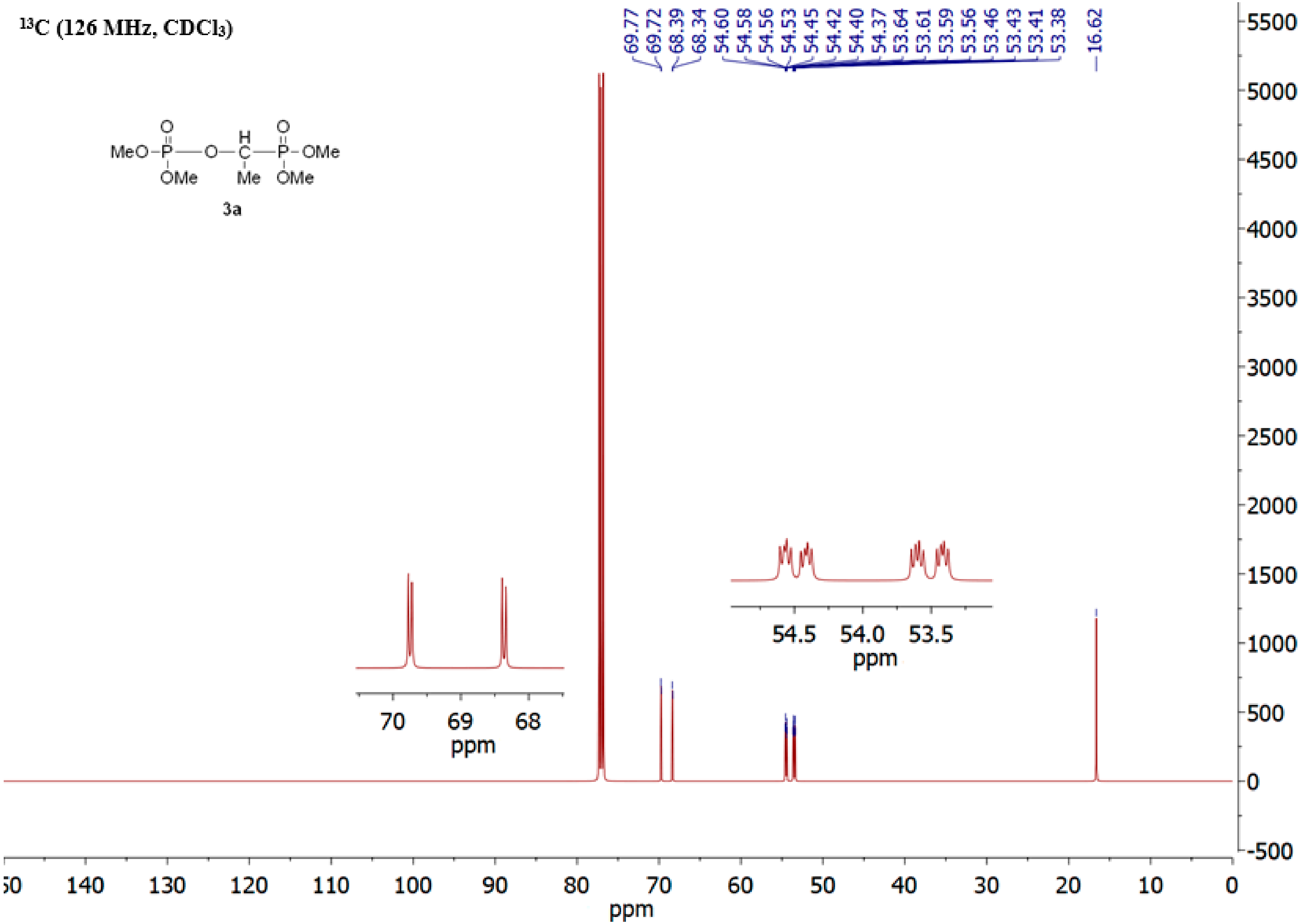

13C (126 MHz, CDCl3)
69.77
69.72
68.39
68.34
54.60
54.58
54.56
54.53
54.45
54.42
54.40
54.37
53.64
53.61
53.59
53.56
53.46
53.43
53.41
53.38
16.62
ppm
MeO
OMe
Me

3a

^{1}H (500 MHz, $CDCl_3$)

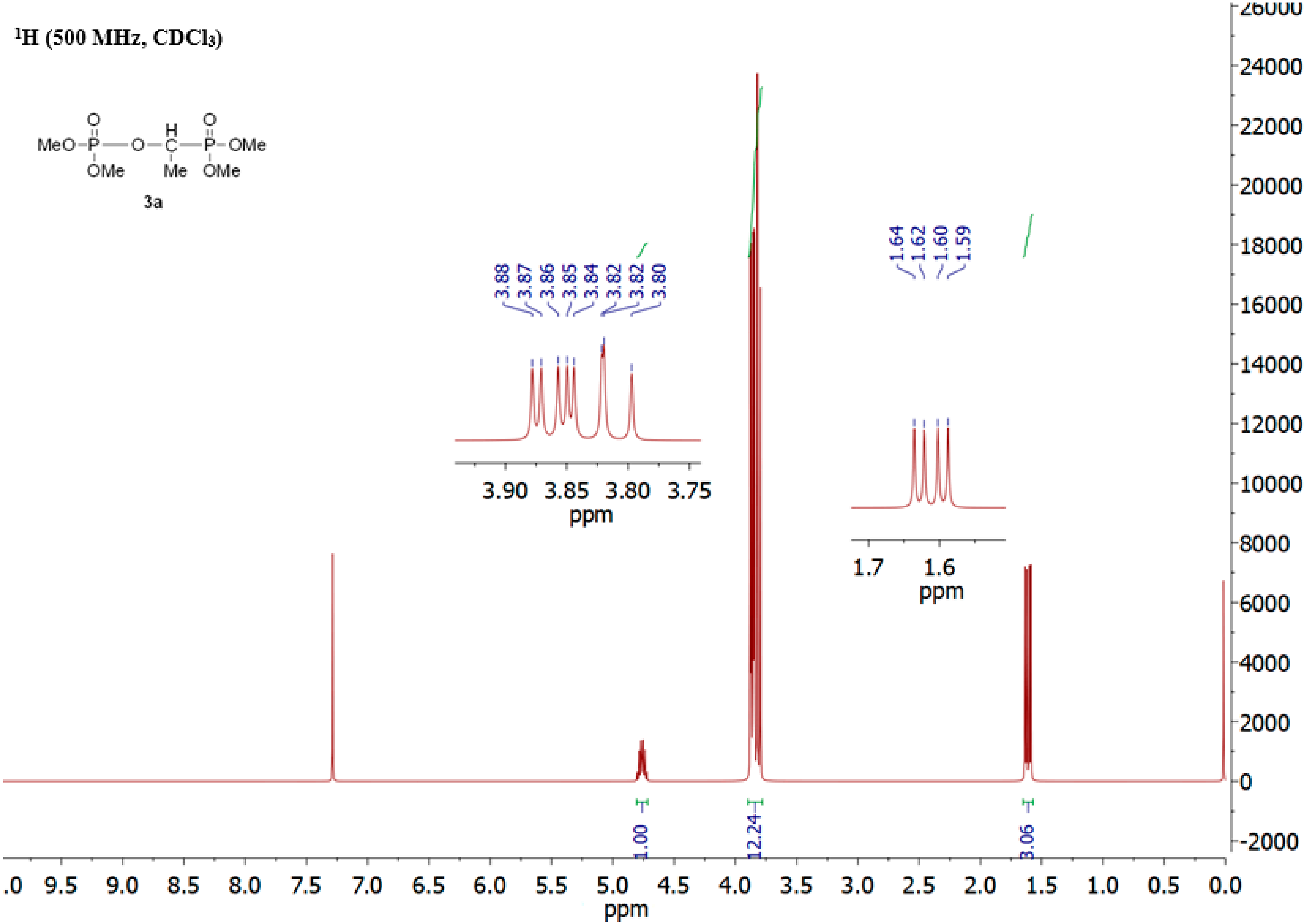

3a

^{31}P (202 MHz, $CDCl_3$)

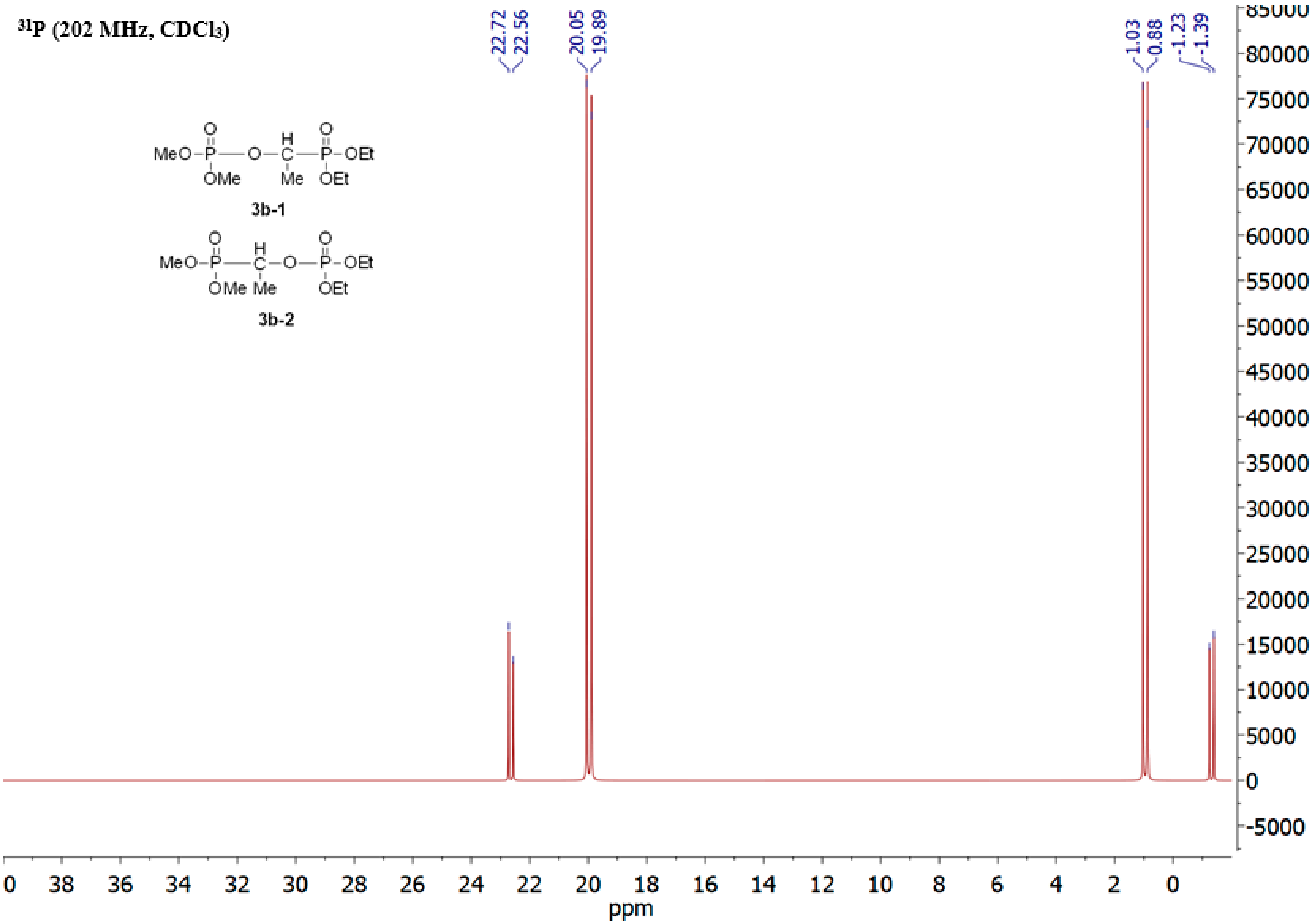

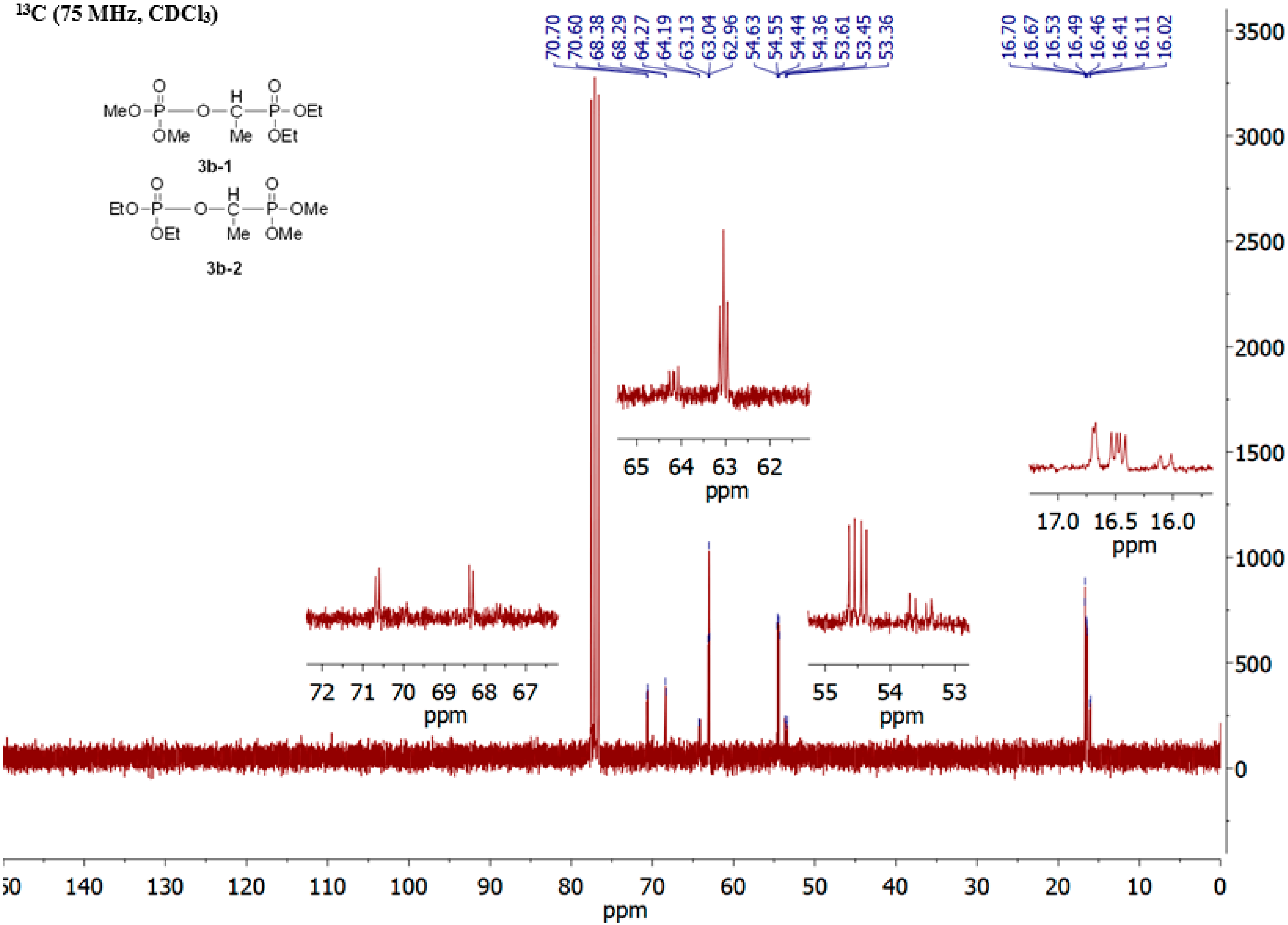
13C (75 MHz, CDCl3)
70.70
70.60
68.38
68.29
64.27
64.19
63.13
63.04
62.96
54.63
54.55
54.44
54.36
53.61
53.45
53.36
16.70
16.67
16.53
16.49
16.46
16.41
16.11
16.02
MeO
OMe
Me
OEt
OEt
3b-1
EtO
OEt
Me
OMe
OMe
3b-2
ppm

^{1}H (500 MHz, CDCl$_3$)

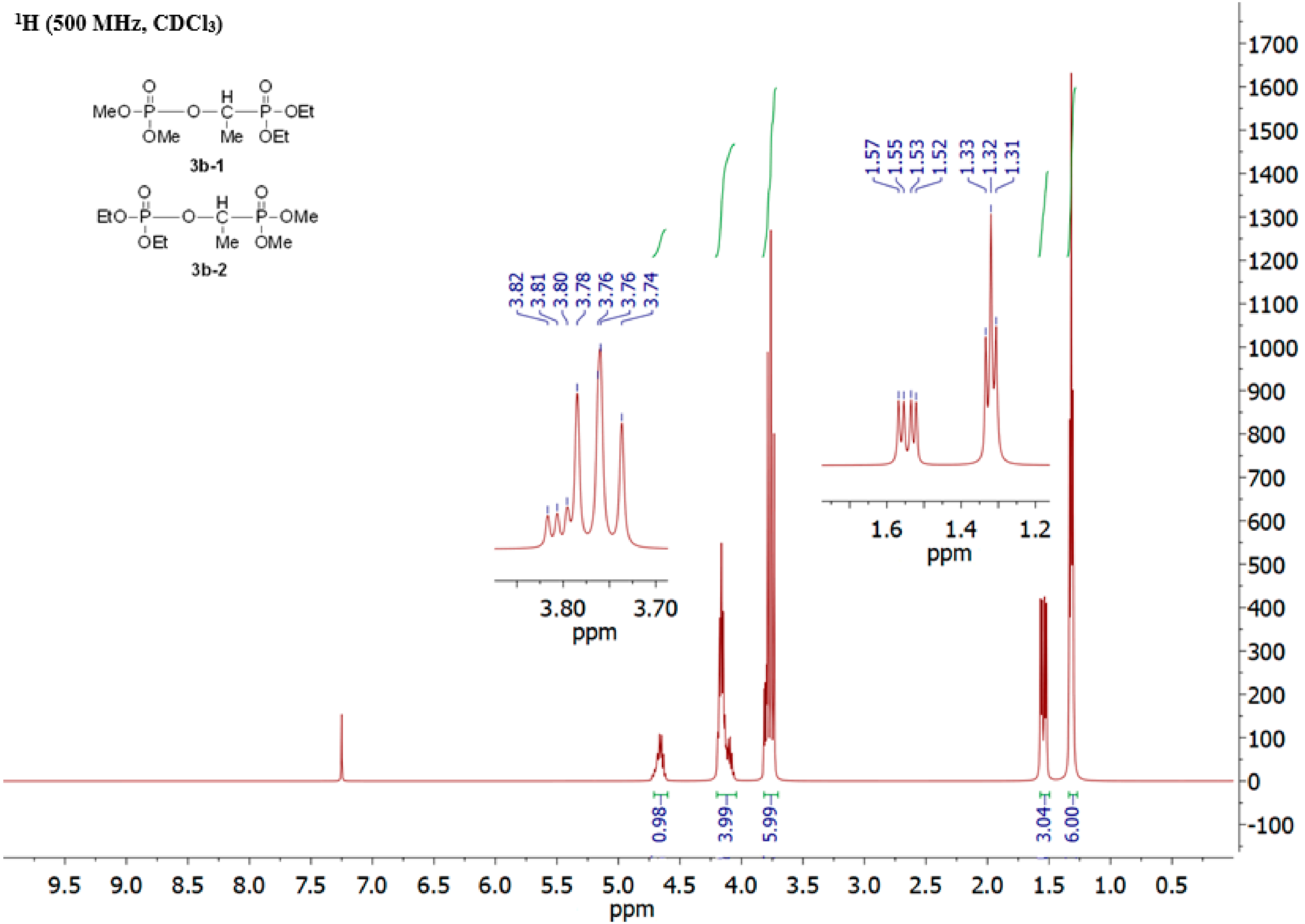

^{31}P (202 MHz, CDCl3)

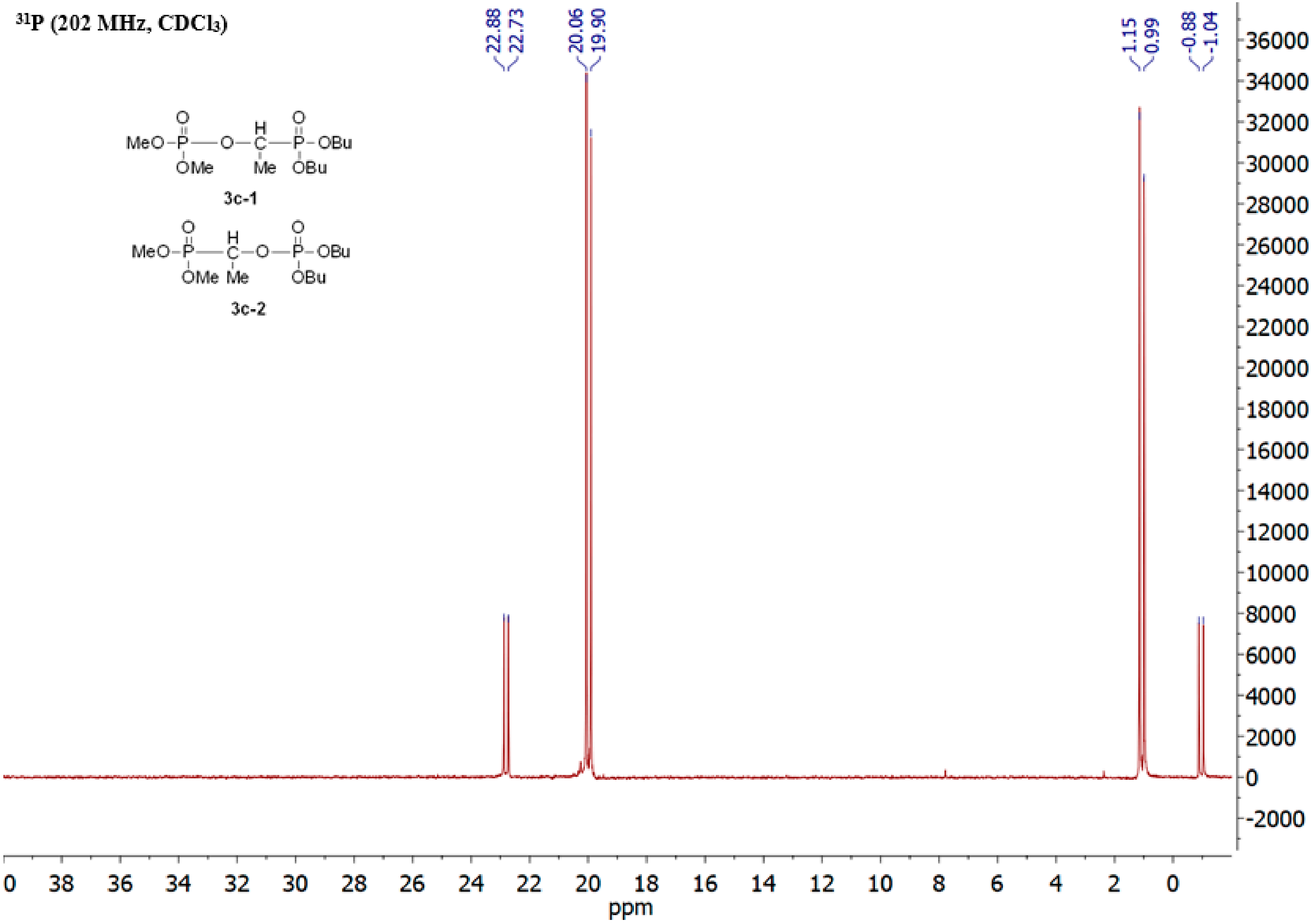

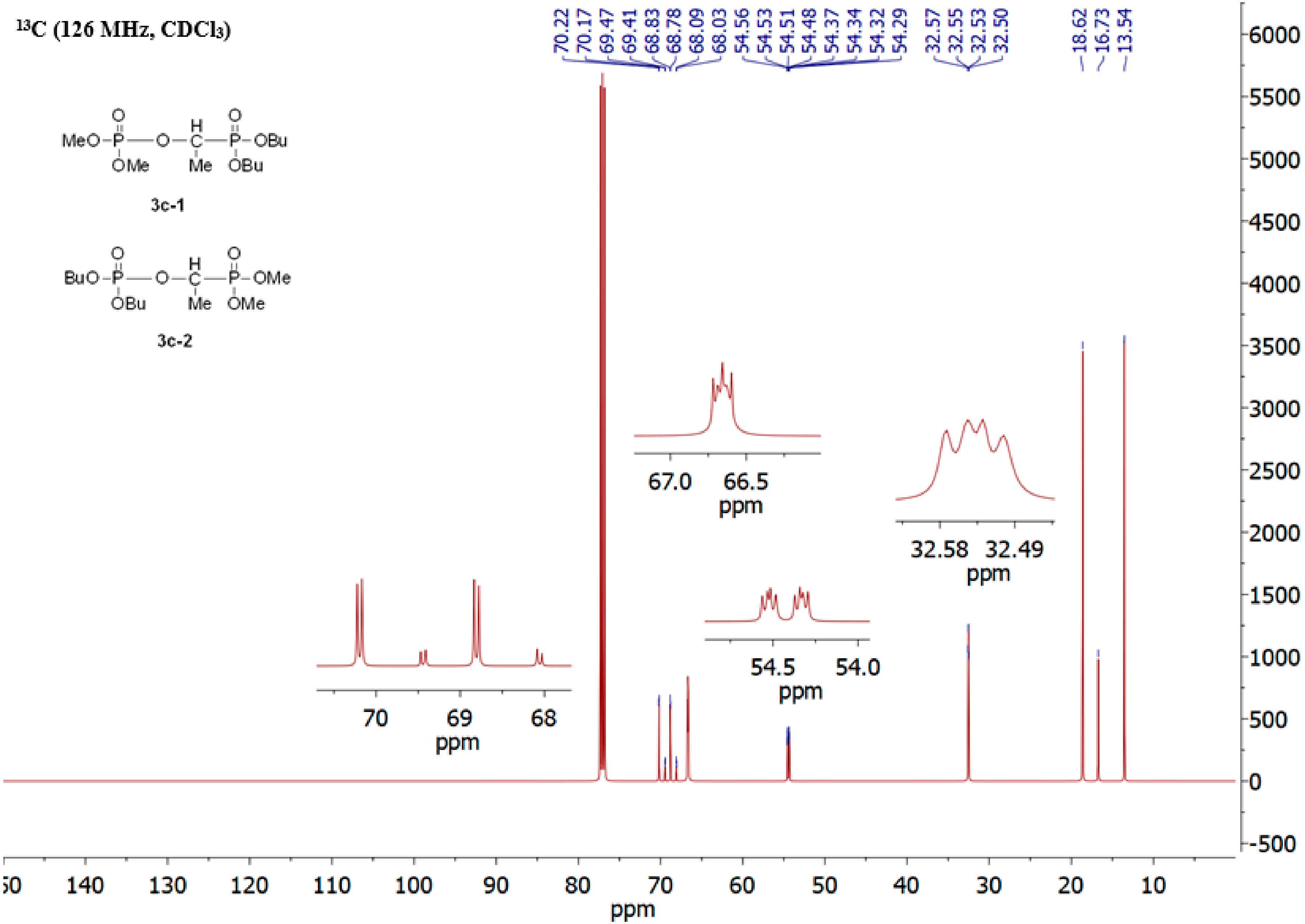

^{13}C (126 MHz, CDCl$_3$)
3c-1
3c-2
70.22
70.17
69.47
69.41
68.83
68.78
68.09
68.03
54.56
54.53
54.51
54.48
54.37
54.34
54.32
54.29
32.57
32.55
32.53
32.50
18.62
16.73
13.54
ppm

^{1}H (500 MHz, $CDCl_3$)

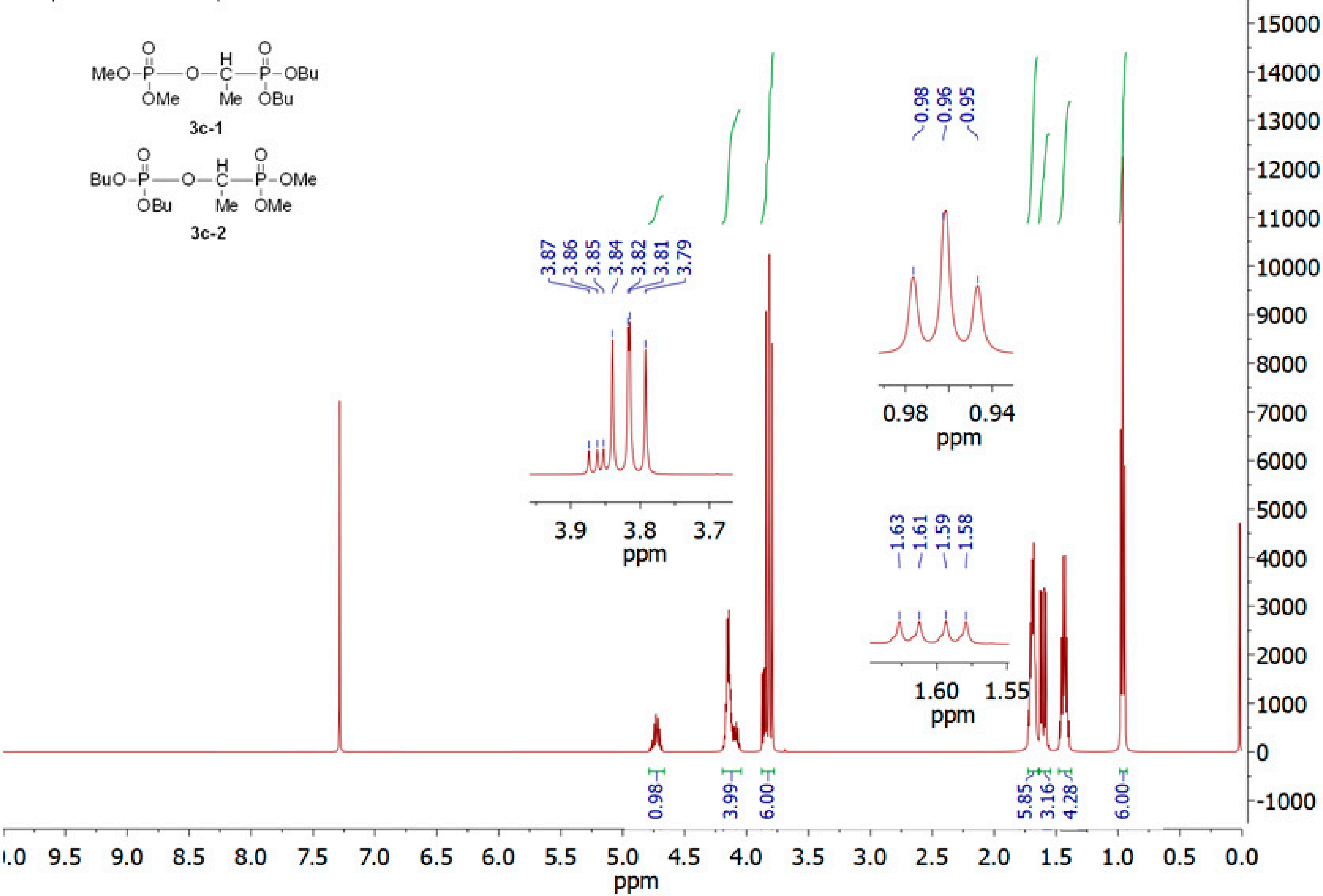

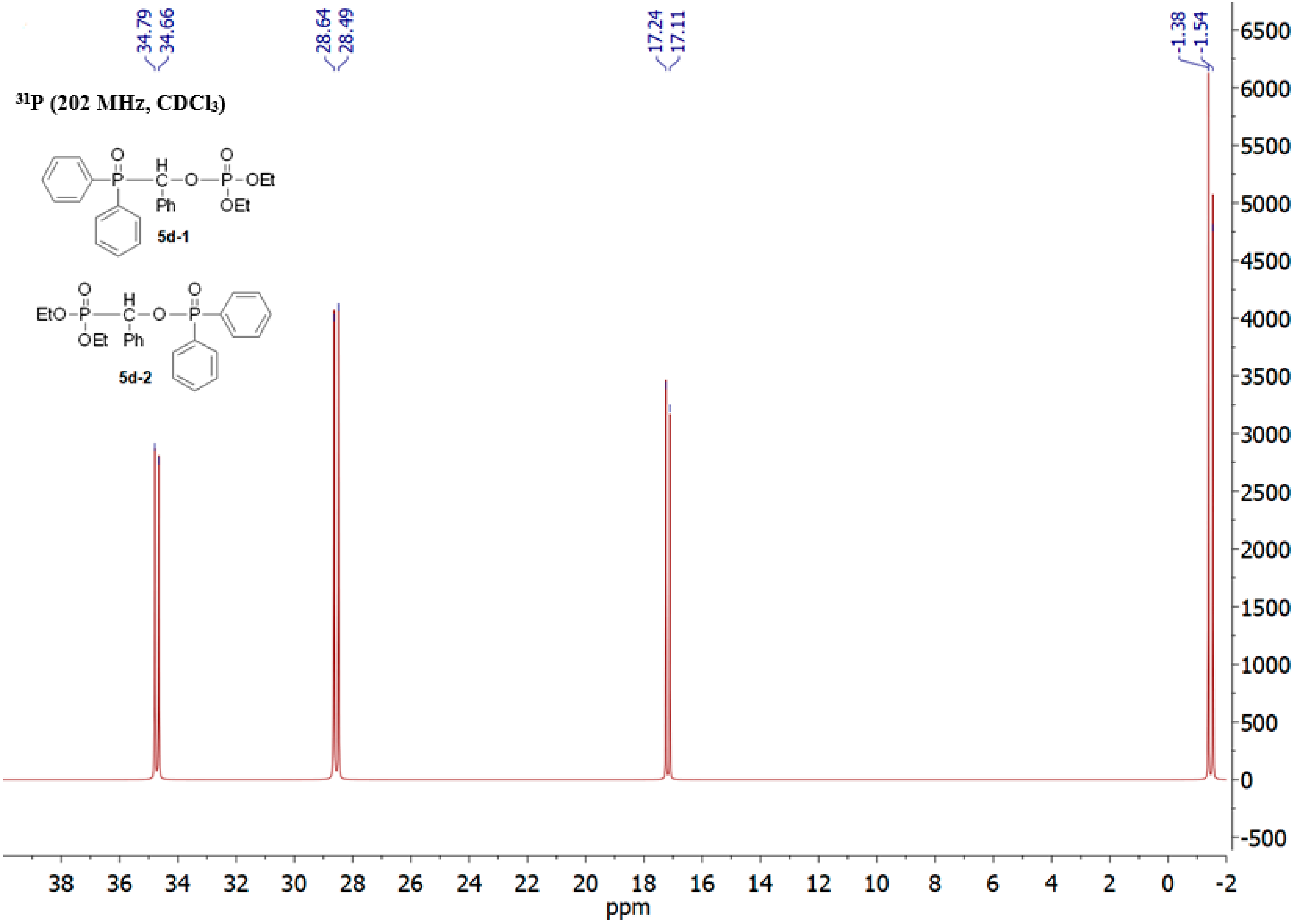

34.79
34.66
28.64
28.49
17.24
17.11
-1.38
-1.54
31P (202 MHz, CDCl3)
5d-1
5d-2
ppm

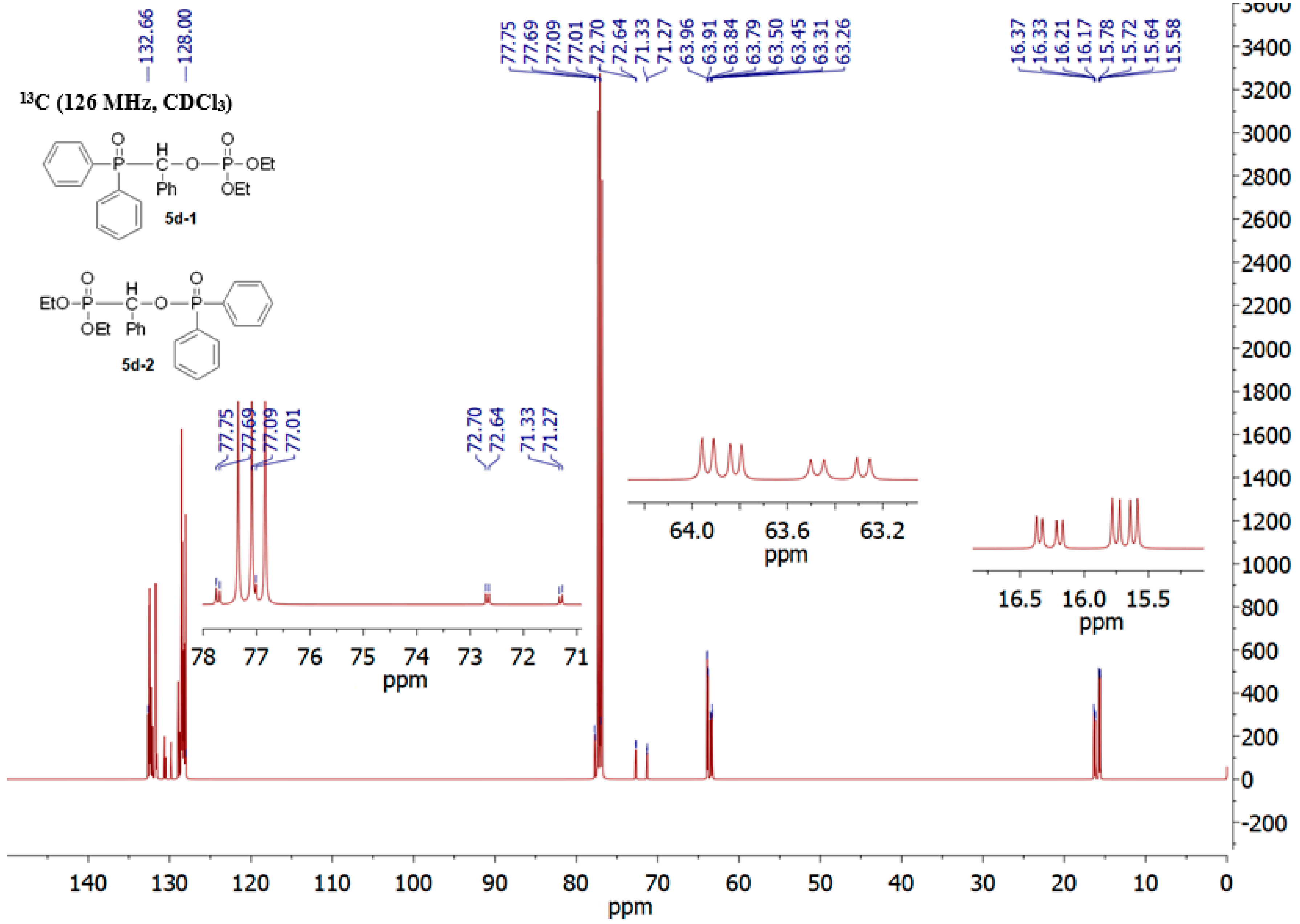

132.66
128.00
13C (126 MHz, CDCl3)
5d-1
5d-2
77.75
77.69
77.09
77.01
72.70
72.64
71.33
71.27
63.96
63.91
63.84
63.79
63.50
63.45
63.31
63.26
16.37
16.33
16.21
16.17
15.78
15.72
15.64
15.58
ppm

^{1}H (500 MHz, CDCl$_3$)

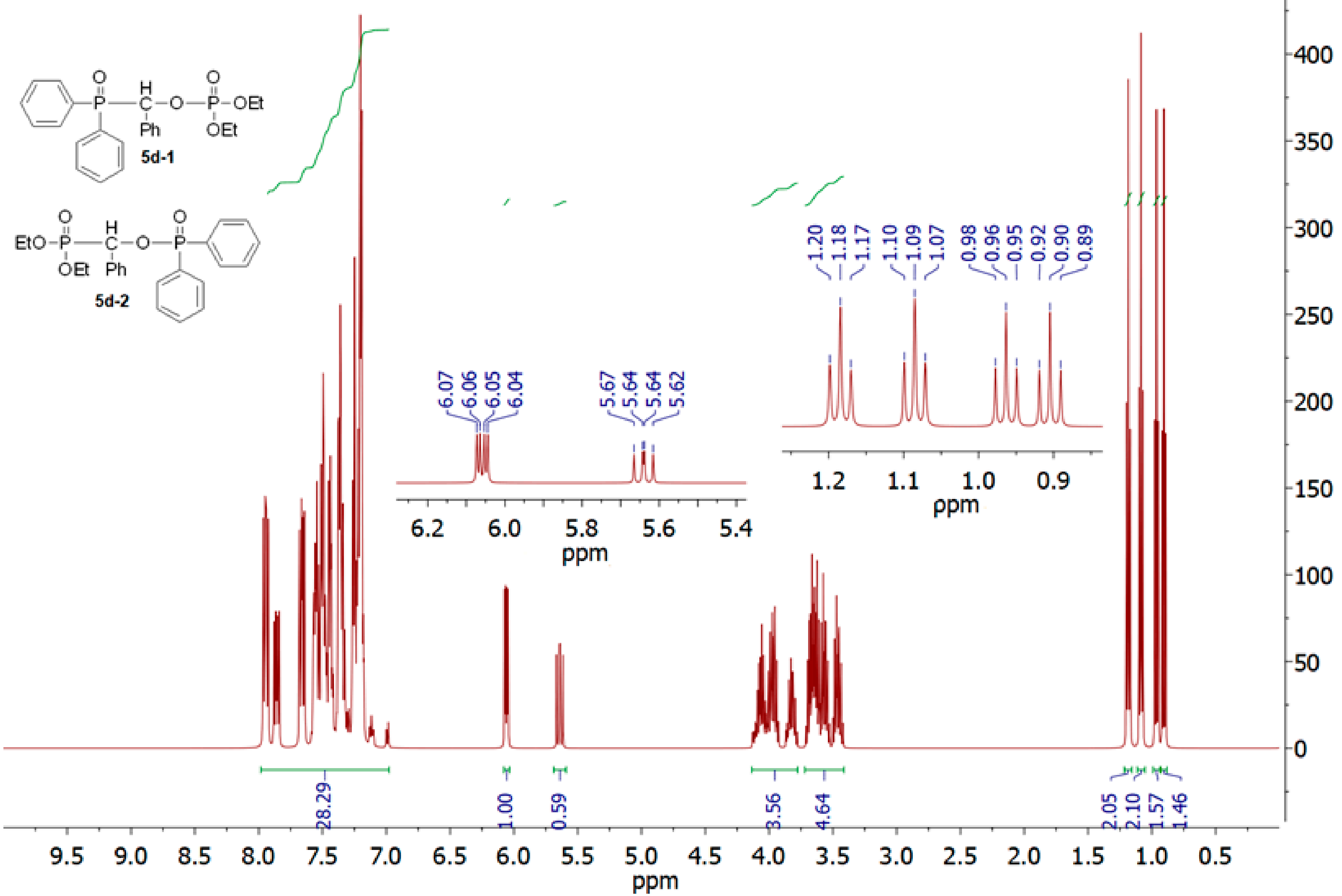

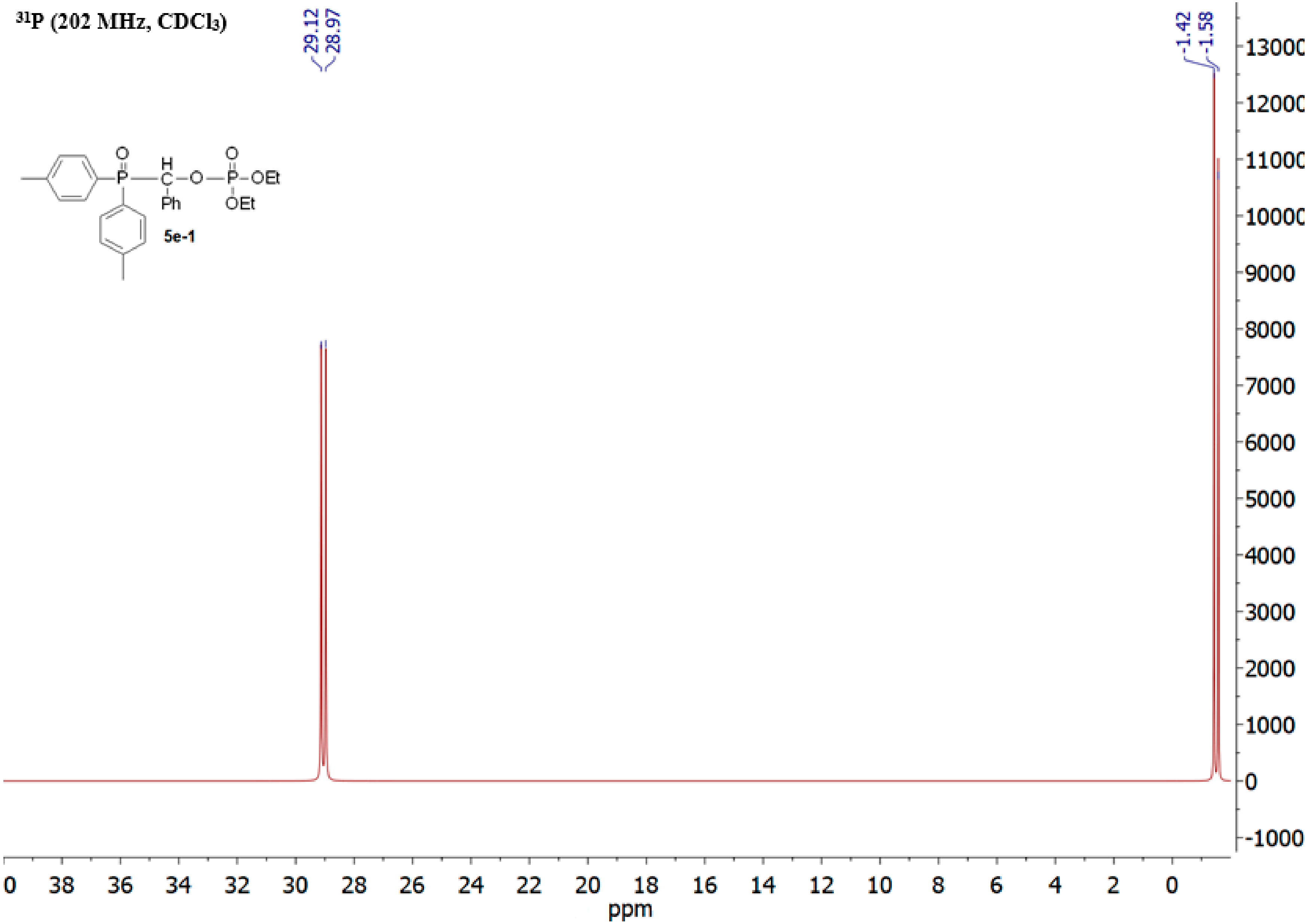

31P (202 MHz, CDCl3)
29.12
28.97
-1.42
-1.58
O
P
H
C
O
O
P
OEt
Ph
OEt
5e-1
13000
12000
11000
10000
9000
8000
7000
6000
5000
4000
3000
2000
1000
0
-1000
0
38
36
34
32
30
28
26
24
22
20
18
16
14
12
10
8
6
4
2
0
ppm

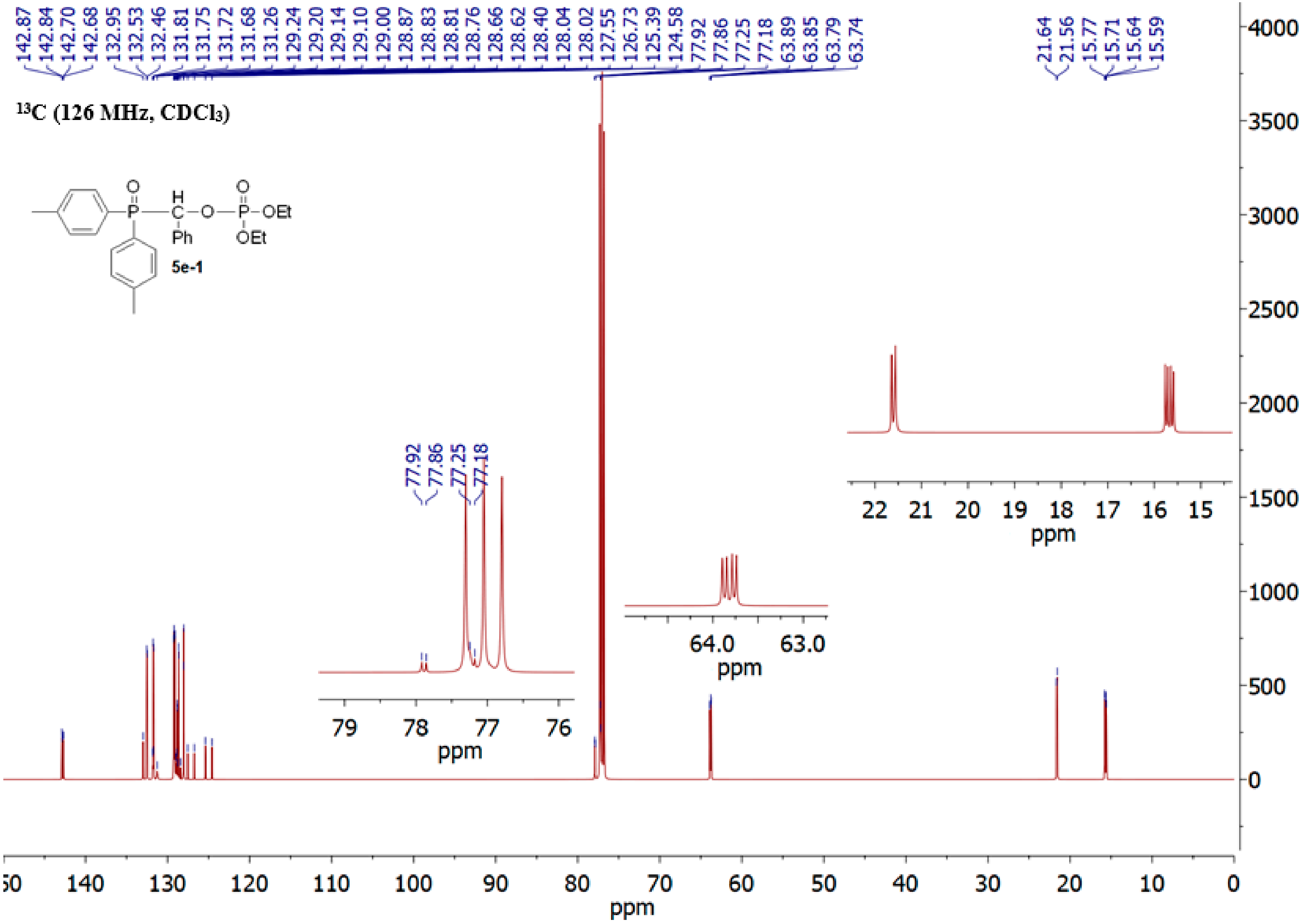
142.87
142.84
142.70
142.68
132.95
132.53
132.46
131.81
131.75
131.72
131.68
131.26
129.24
129.20
129.14
129.10
129.00
128.87
128.83
128.81
128.76
128.66
128.62
128.40
128.04
128.02
127.55
126.73
125.39
124.58
77.92
77.86
77.25
77.18
63.89
63.85
63.79
63.74
21.64
21.56
15.77
15.71
15.64
15.59
13C (126 MHz, CDCl3)
OEt
Ph
OEt
5e-1
ppm

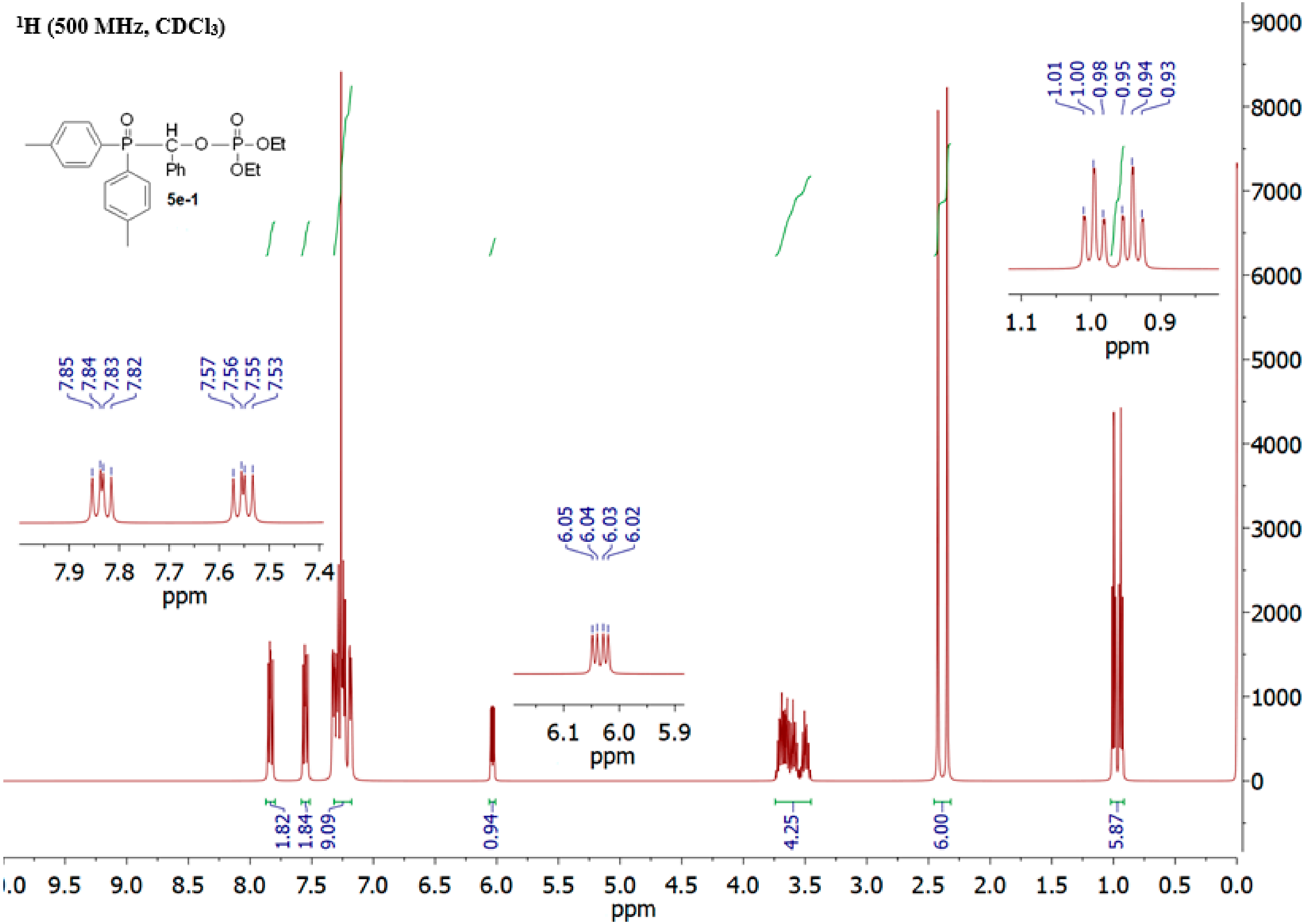

^{1}H (500 MHz, CDCl3)
5e-1
7.85
7.84
7.83
7.82
7.57
7.56
7.55
7.53
6.05
6.04
6.03
6.02
1.01
1.00
0.98
0.95
0.94
0.93
1.82
1.84
9.09
0.94
4.25
6.00
5.87
ppm

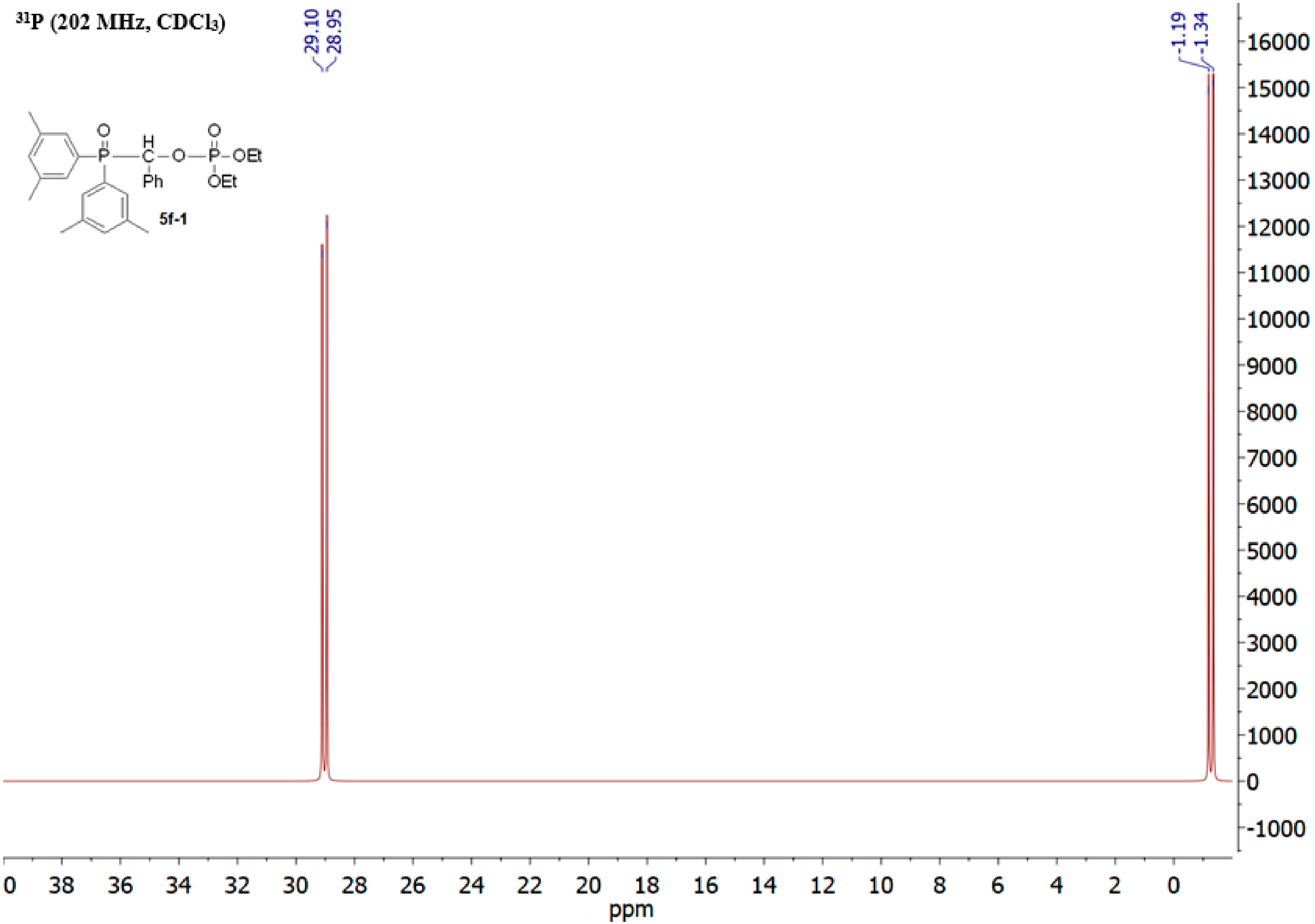

31P (202 MHz, CDCl3)
29.10
28.95
-1.19
-1.34
O
P
H
C
O
P
OEt
Ph
OEt
5f-1
16000
15000
14000
13000
12000
11000
10000
9000
8000
7000
6000
5000
4000
3000
2000
1000
0
-1000
0
38
36
34
32
30
28
26
24
22
20
18
16
14
12
10
8
6
4
2
0
ppm

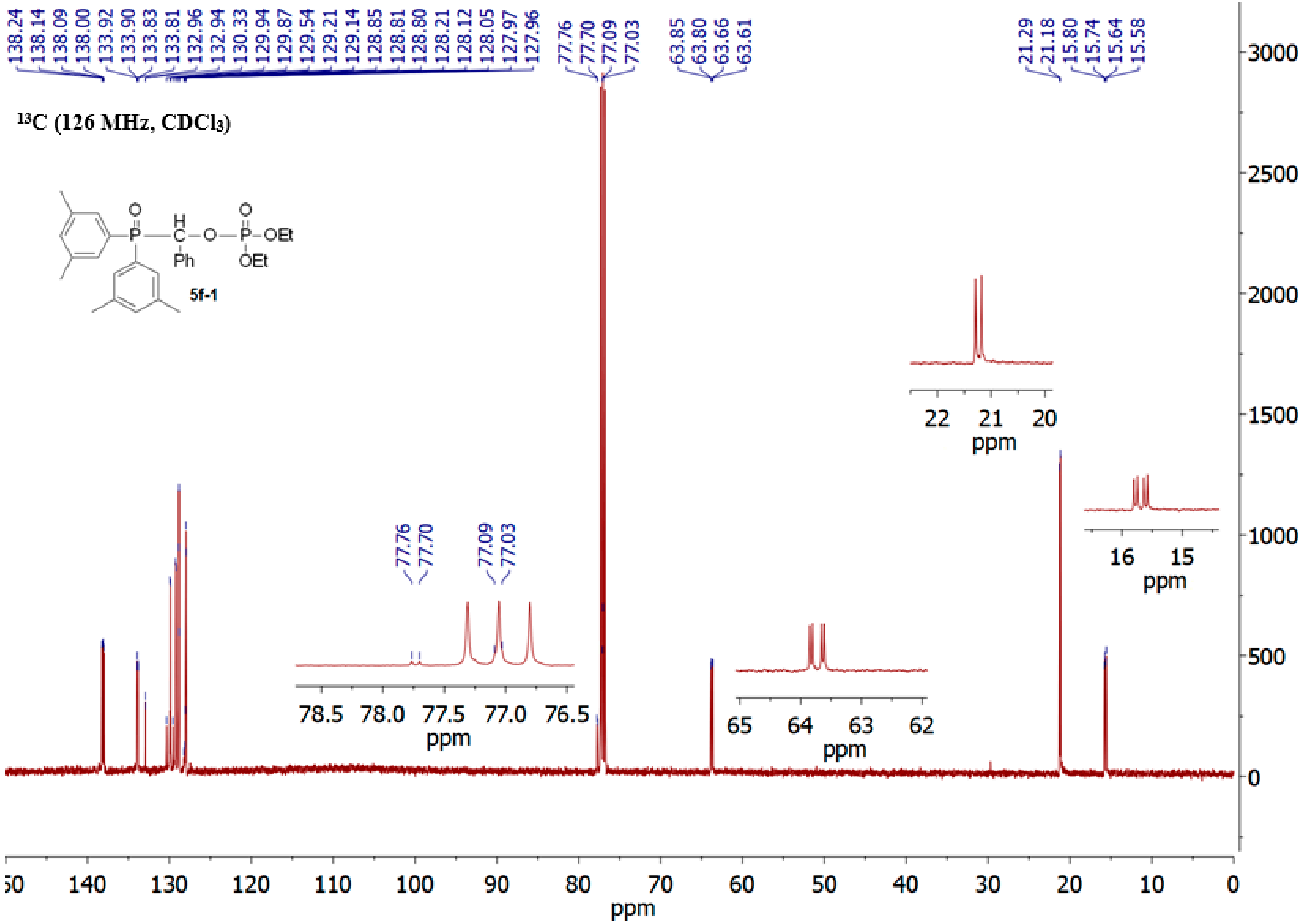
138.24
138.14
138.09
138.00
133.92
133.90
133.83
133.81
132.96
132.94
130.33
129.94
129.87
129.54
129.21
129.14
128.85
128.81
128.80
128.21
128.12
128.05
127.97
127.96
77.76
77.70
77.09
77.03
63.85
63.80
63.66
63.61
21.29
21.18
15.80
15.74
15.64
15.58
13C (126 MHz, CDCl3)
OEt
OEt
Ph
5f-1
ppm

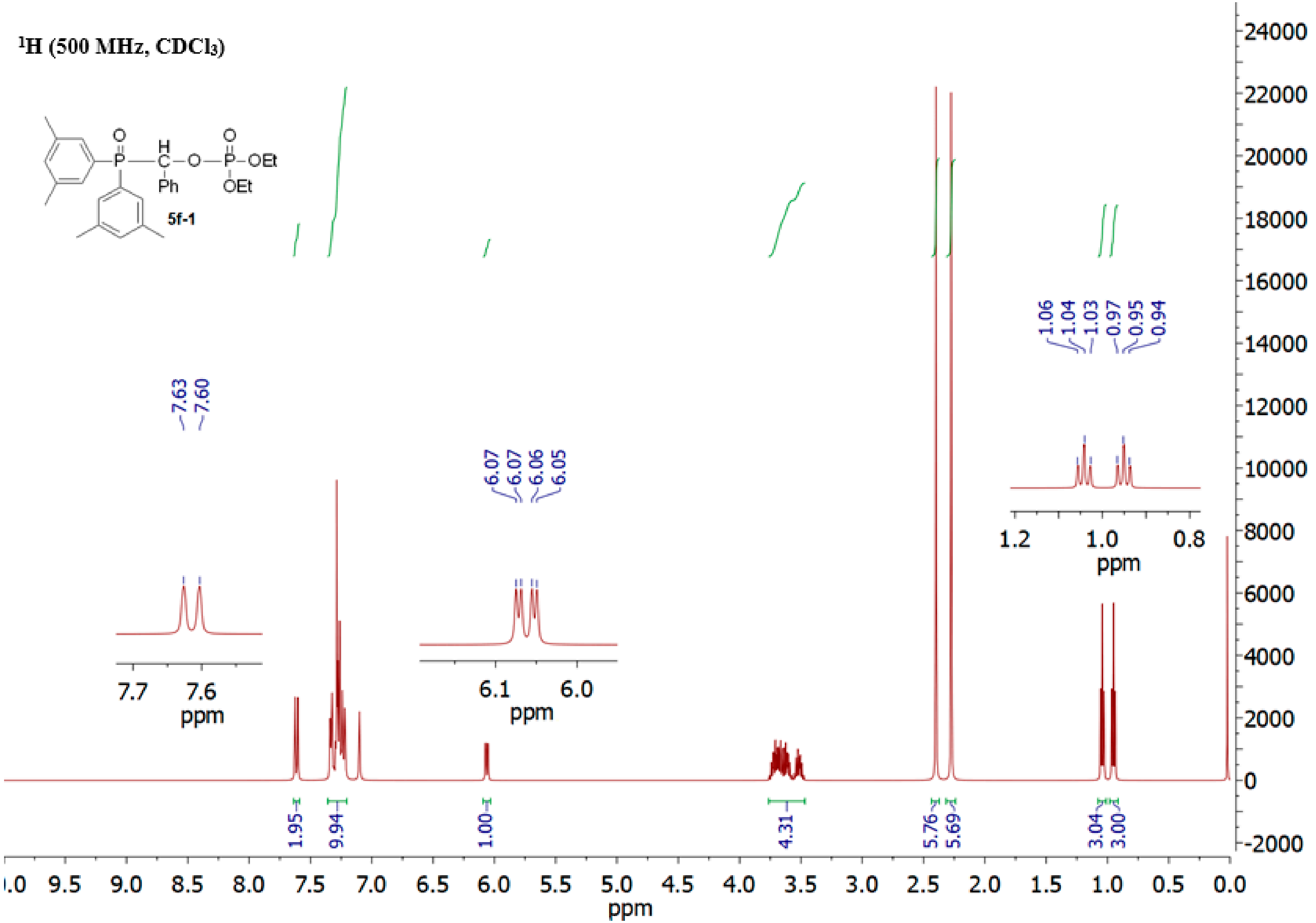

¹H (500 MHz, CDCl₃)
OEt
OEt
Ph
5f-1
7.63
7.60
6.07
6.07
6.06
6.05
1.06
1.04
1.03
0.97
0.95
0.94
1.95
9.94
1.00
4.31
5.76
5.69
3.04
3.00
ppm
